# Perpendicular crossing chains enable high mobility in a noncrystalline conjugated polymer

**DOI:** 10.1073/pnas.2403879121

**Published:** 2024-09-03

**Authors:** Jack F. Coker, Stefania Moro, Anders S. Gertsen, Xingyuan Shi, Drew Pearce, Martin P. van der Schelling, Yucheng Xu, Weimin Zhang, Jens W. Andreasen, Chad R. Snyder, Lee J. Richter, Matthew J. Bird, Iain McCulloch, Giovanni Costantini, Jarvist M. Frost, Jenny Nelson

**Affiliations:** ^a^Department of Physics, Imperial College London, London SW7 2AZ, United Kingdom; ^b^School of Chemistry, University of Birmingham, Birmingham B15 2TT, United Kingdom; ^c^Department of Energy Conversion and Storage, Technical University of Denmark, Kongens Lyngby 2800, Denmark; ^d^Department of Materials Science and Engineering, Delft University of Technology, Delft 2628 CD, The Netherlands; ^e^Department of Physics, Cavendish Laboratory, University of Cambridge, Cambridge CB3 0HE, United Kingdom; ^f^King Abdullah University of Science and Technology Solar Center, Division of Physical Sciences and Engineering, King Abdullah University of Science and Technology, Thuwal 23955, Kingdom of Saudi Arabia; ^g^Material Science and Engineering Division, National Institute of Standards and Technology, Gaithersburg, MD 20899; ^h^Chemistry Division, Brookhaven National Laboratory, Upton, NY 11973; ^i^Department of Chemistry, University of Oxford, Oxford OX1 3TA, United Kingdom; ^j^Department of Chemistry, Imperial College London, London W12 0BZ, United Kingdom

**Keywords:** organic electronics, conjugated polymers, microstructure, charge transport

## Abstract

The nature of interchain π-system contacts, and their relationship to hole transport, are elucidated for the high-mobility, noncrystalline conjugated polymer C16-IDTBT by the application of scanning tunneling microscopy, molecular dynamics, and quantum chemical calculations. The microstructure is shown to favor an unusual packing motif in which paired chains cross-over one another at near-perpendicular angles. By linking to mesoscale microstructural features, revealed by coarse-grained molecular dynamics and previous studies, and performing simulations of charge transport, it is demonstrated that the high mobility of C16-IDTBT can be explained by the promotion of a highly interconnected transport network, stemming from the adoption of perpendicular contacts at the nanoscale, in combination with fast intrachain transport.

## Significance

Conjugated polymers are attractive semiconductor materials because of their processability, chemical tunability, flexibility, and biocompatibility, but are limited by poor charge-carrier mobilities. Surprisingly, some of the best-performing polymers are noncrystalline, which makes it challenging to pinpoint the microstructural origin of their high mobilities. Here, we show that the high mobility of one such polymer results neither from structural order nor from strong coupling, but from an extensive interconnected transport network, itself enabled by a combination of chain rigidity and a perpendicular chain packing motif. These results show how bulk electronic properties can be induced through chemical design that promotes certain structural features. Such insights can accelerate the rational design of flexible and robust electronic materials.

**Fig. 1.**
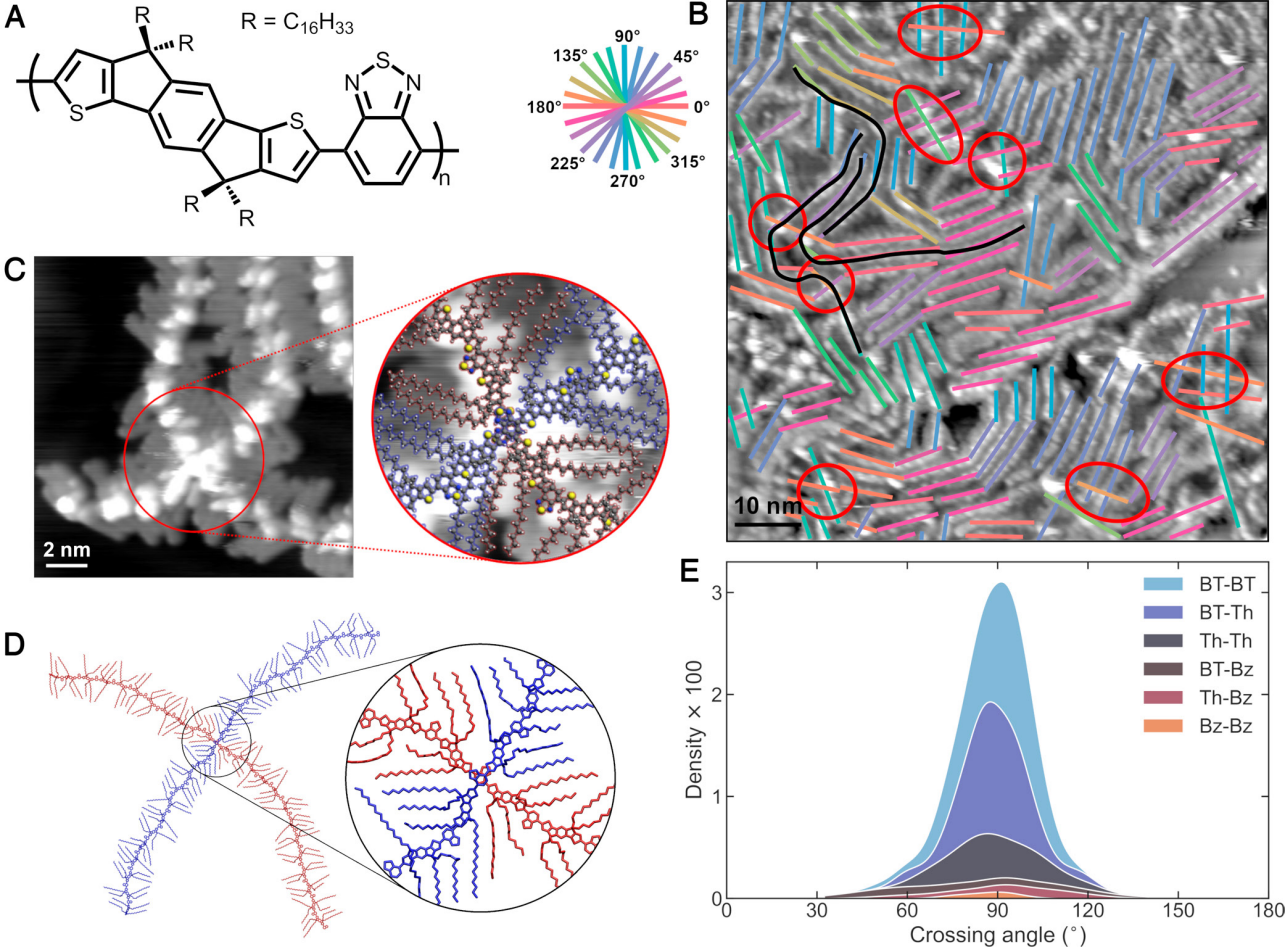
Arrangement of C16-IDTBT chains constrained to surfaces. (*A*) Chemical structure of C16-IDTBT. (*B*) STM image showing chains deposited on an atomically flat gold substrate. Overlaid colored lines indicate the local orientation of backbones. Their clustering reveals the existence of domains of locally parallel-orientated chains. Black lines highlight examples of chains bending and participating in multiple domains. Examples of interchain backbone contact are circled in red. (*C*) High-resolution STM image showing a crossing point between two polymers. The *Inset* shows a zoomed-in image with a geometry-optimized molecular model overlaid. Red represents the *Bottom* polymer, and blue the *Top* one. (*D*) An example MD structure resulting from simulation of C16-IDTBT chains deposited on substrate. The chains are colored red (*Bottom*) and blue (*Top*) to help distinguish them. (*E*) Stacked distributions of chain crossing angles from MD simulations. Crossing angles cluster around 90°. Each color represents contacts between particular types of moiety on the two backbones. Areas of colored regions represent occurrence of different moiety contacts, with larger areas indicating more contacts of this type.

Conjugated polymers (CPs) can offer intrinsic advantages of synthetic tunability, flexibility, and low-cost fabrication to semiconductor devices. A material that has attracted significant interest due to its ability to reach experimental hole mobilities as high as 3.6 cm^2^ V^−1^ s^−1^ in field-effect transistor (FET) devices, despite lacking signs of long-range structural order, is the donor-acceptor polymer C16-IDTBT (1–3) (indacenodithiophene-co-benzothiadiazole). The chemical structure of C16-IDTBT is shown in Fig. 1*A*. Near-amorphous polymers, such as C16-IDTBT, have been highlighted as particularly suitable for wearable electronics applications due to the resilience of their electronic properties to degradation under mechanical strain (4–8). However, increased charge mobilities are required to achieve improvements in device performance.

Electronic transport in CPs occurs via two pathways: intrachain motion along chain backbones, and interchain transfer at locations of backbone contact. Solid-state microstructure is a key determinant of mobility, affecting both pathways (9–12). The backbone of C16-IDTBT is colinear and planar, indicating support for rapid intrachain charge motion (13). This is partly based on considerations of the chemical structure. The bonds linking the IDT and BT units are collinear, and torsional rotation is restricted by a relatively high energy barrier (13, 14). Simulations of intrachain transport indicate support for on-chain mobilities of ~3 cm^2^ V^−1^ s^−1^ at room temperature (15). This is larger than most experimental measurements of bulk mobility, suggesting that the transfer of charges between backbones acts as a limiting bottleneck to transport. This view is supported by evidence that mobility increases with chain length, only leveling off for ultrahigh molar mass samples of 500 kg mol^−1^ and beyond (5).

Interchain charge transfer is largely dependent on the polymer film assembly structure. Diffraction experiments show a lack of long-range order that would indicate the presence of crystallites in C16-IDTBT (1, 2). However, recent works have pointed toward other signs of unconventional ordering at the mesoscale. Domains of aligned backbones have been observed via low-does high-resolution transmission electron microscopy (HRTEM) (16) and atomic force microscopy (AFM) (17). On its own, packing within these domains is unlikely to be responsible for the efficient transfer of charges between chains, since adjacent backbones are separated by a relatively large spacing of 1.6 nm (17). Importantly however, analysis of the HRTEM measurements also showed a degree of ordering between domains, with backbones preferentially crossing at 20° and 90° in regions of domain overlap (16).

A very recent work proposed a microstructural organization for C16-IDTBT involving a mesh-like arrangement of parallel and perpendicular chains contacting via BT units (18), with similarities to cross-hatched crystallite structures described in some semicrystalline materials (19, 20). Although distinct from the disordered microstructure of C16-IDTBT, these 2D crystallite structures demonstrate the benefits to transport that can be gained by moving away from a traditional arrangement of parallel packed backbones in CPs: Takacs et al. propose that the higher connectivity between chains in such microstructures provide additional interchain contact sites and isotropy of transport pathways, allowing charges to navigate around blockages and traps (19). The benefits of a highly connected transport network have been demonstrated by graph network analyses applied to small molecule systems (21–23), and analyses of tie chains in semicrystalline polymer systems (11, 24–26).

Although we know comparatively less about the nanoscale picture in C16-IDTBT—i.e., the local scales most relevant to interchain contact and charge transfer rates (27, 28)—it is generally believed that backbone contacts involve the BT unit of the monomer. This was suggested by Thomas et al. based on the fact that the attachment locations of the four bulky side chains are likely to sterically inhibit contact between IDT units (29, 30). This suggestion of BT contact is also present in Makki et al.’s proposed structure (18). Solid-state NMR measurements of another BT containing copolymer, suggested that BT–BT contacts dominate interchain interactions (31).

Whereas the microstructure determines transport, the chemical structure of the CP chain itself is the main determinant of the microstructure. One of the main avenues to control the packing arrangement of polymers is via modifications of the side chains. The effect of side chain choice on transport in IDTBT has been demonstrated previously, with the highest mobility displayed by the variant with the longest side chains (32).

The side chain attachment frequency may be altered by modifying the IDTBT backbone. As summarized by Wadsworth et al., attempts have been made to enhance the frequency of interchain contacts by symmetrically extending the donor unit, giving rise to a family of polymers inspired by the IDTBT archetype (33, 34). Lengthening the donor unit is designed to maintain the planarity of the monomer while increasing the spacing between side chain attachment points, allowing more room for backbones to make contact. This strategy appears to have been successful in the case of dithiopheneindenofluorene-co-benzothiadiazole (TIFBT). Molecular dynamics (MD) and kinetic Monte Carlo (KMC) transport simulations indicate that TIFBT forms a higher density of short contacts than IDTBT, thus explaining the higher mobilities exhibited (30, 35). This is a promising result, demonstrating that enhancement of contact occurrence is an avenue for improving transport in noncrystalline polymers.

The purpose of this study is to develop a coherent understanding of the microstructure of C16-IDTBT, and its impact on charge transport. We aim to address the following question: What aspect of the chemical structure of C16-IDTBT is responsible for the high hole mobilities it exhibits?

To answer this, we apply a combination of experimental and computation techniques that afford us a nanoscale view of interchain contacts. These include scanning tunneling microscopy (STM) imaging and atomistic MD simulations. We link our findings at the nanoscale with larger-scale behavior by comparison with coarse-grained (CG) MD models of solid-state microstructure. These techniques are complemented by quantum chemical calculations used to evaluate the different interactions occurring between chains in contact and explore the relationship of contact geometry with energy and electronic coupling. Finally, we employ KMC simulations to assess the relationship between the predicted microstructure of C16-IDTBT and its transport behavior.

## 1. Results

### 1.1. Single Chain Behavior.

To characterize the conformation of individual polymer chains and to help validate our MD approach, we carried out small-angle neutron scattering (SANS) on C16-IDTBT polymer samples of different molar masses and concentrations in solution. Analyses of the scattering data with a flexible cylinder model suggest that the persistence length *P* of the polymer lies in the range 6.8 nm < *P* < 39 nm, confirming the rigid structure of the backbone (see *SI Appendix*, section S1 for more details). To complement these measurements, we also assessed *P* using MD simulations of chains in chloroform, finding a value of 27.0 ± 6.7 nm (see *SI Appendix*, section S5 for more details). These high values of *P* are supported by prior grazing incidence X-ray diffraction (GIXD) measurements (2), wherein the Scherrer coherence length for the (001) (c-axis) reflection for C16-IDTBT was determined to be 22 nm, which could likely be assumed to be a lower bound on *P*.

In addition, we carried out pulse radiolysis time-resolved microwave conductivity (PR-TRMC) measurements on C16-IDTBT chains dispersed in solution to estimate the mobility for holes confined to the backbone of single chains, *μ_intra_*. We were able to determine a lower limit of *μ_intra_* ≥ 1 cm^2^ V^−1^ s^^−1^^ (see *SI Appendix*, section S2 for more details).

### 1.2. Contact between Chains Deposited onto Substrate.

#### 1.2.1. STM.

Monolayer films of C16-IDTBT were prepared on atomically flat Au(111) substrate by vacuum-electrospray deposition (ESD). This was followed up in situ by ultrahigh vacuum STM measurements, producing images such as Fig. 1*B*. Individual chains can be clearly resolved, with backbones appearing as strands dotted with bright spots (*SI Appendix*, Fig. S4). Side chains are visible as darker features that interdigitate in the space between the backbones (*SI Appendix*, Fig. S6). By considering the attachment points of the side chains, first, the IDT units and then the BT units can be identified (*SI Appendix*, section S3) (36). As with all CPs deposited in this manner, the backbones adopt a uniformly face-on orientation to the metallic substrate (36–40). For C16-IDTBT, this is also largely the case for thin films with thicknesses up to several tens of nm (2), suggesting the STM images provide a good representation of the initial stages of thin film formation. Images such as Fig. 1*B*, show domains of aligned polymer chains, marked by colored line segments, as well as several instances of interchain contact, marked with red circles.

Because the side chains prevent backbones from making contact edgewise, instances of backbone contact occur at locations where chains are arranged one on top of the other. Importantly, we observe that, at all contact locations, chains cross-over one another at large angles, close to perpendicular. Moreover, the STM images show that, although the relative orientation of crossing polymers can vary significantly away from points of intersection, the polymer backbones tend to bend as they approach the crossing point itself, to align in a perpendicular fashion (*SI Appendix*, section S3.2). When carefully fitted with geometry-optimized molecular models (*SI Appendix*, section S3.1), high-resolution STM images also show that observed points of backbone contact appear to involve the BT units of both chains (*Inset* in Fig. 1*C*). Around these contact points, the closest side chains tend to splay out in a neat, dovetailed pattern. Somewhat akin to interdigitation, this pattern is likely the result of side chains optimizing intermolecular interactions within the constrained geometry of the crossing formation. This predominance of high-angle chain crossings is consistent with the observation of preferential domain crossing angles of ~90° by Cendra et al. (16). STM thus provides additional information to the HRTEM analysis thanks to its higher spatial resolution.

The spacing between parallel backbones in the domains observed in STM is 2.7 ± 0.2 nm, wider than the ~1.6 nm periodicity observed using AFM in the top layers of thin films (17). This discrepancy likely arises because substrate interactions encourage side chains to lie flat in the monolayer, where they organize themselves in an interdigitated fashion. In thin films, the side chains have a higher degree of freedom and can extend above and below the backbones, allowing parallel backbones to pack more closely. However, the backbone conformations are expected to be similar for face-on thin films and surface-adsorbed monolayers, as is confirmed by the direct comparison between HRTEM images of ~40 nm thick C16-IDTBT films and STM images of ESD monolayers. In both HRTEM (16) and STM measurements, the observed domains of similarly aligned polymers show a lateral extension of ~10 nm (highlighted by the colored line segments in Fig. 1*B*), somewhat shorter than the persistence length observed for chains in solution (Section 1.1). The STM data show that polymers are typically longer than the extension of the domains (see black lines in Fig. 1*B*), implying that individual backbones tend to run parallel for only part of their full length before bending. This is consistent with the idea that adjacent domains observed in the HRTEM analysis may sometimes comprise the same groups of chains bending as they pass from one domain into another, especially when the angle between the orientation of backbones in adjacent domains is small (such as the peak at 20° in the distribution of domain overlap angles in ref. 16).

#### 1.2.2. MD.

To complement the STM experiments, we carried out atomistic MD simulations of pairs of chains deposited onto an Au(111) substrate. Simulation involved two 12mer chains, initiated one above the other, both cofacial to the substrate, with the upper chain rotated and shifted in-plane by a random amount with respect to the lower chain. Both chains were given a small initial velocity downward toward the substrate. 200 systems were initialized and simulated, allowing us to gather statistics pertaining to contact formations in 2D. An example of a contact structure predicted by MD can be seen in Fig. 1*D*. We observe excellent agreement with the structures captured by STM, as revealed by a comparison between Fig. 1 *C* and *D*.

For the modeled systems, we defined the backbone crossing angle as the cosine between the normalized vectors joining the centers of the six-membered rings either side of the ring closest to the other chain (*SI Appendix*, Fig. S16 and section S6.2). Fig. 1*E* shows the distributions of crossing angles adopted by the simulated chain pairs. They are clustered tightly around the perpendicular, with 95% of simulated structures having a crossing angle in the 90° ± 30° range. In addition, side chains are often seen to adopt the same dovetailed pattern observed by STM, as may be seen in Fig. 1*D*. Partial agreement with the STM results is also found when considering the moieties in contact between the two backbones; 77% of contacts involve the BT unit of at least one chain, with BT–BT contact being most common (37%), followed by BT–Th (33%) (where Th = thiophene rings on either edge of the IDT unit).

In all MD systems, the chain backbones closest to the substrate adopted a fully face-on orientation. However, unlike in the experimental case, we found that the upper backbone would sometimes twist fully or partially into an edge-on orientation as it crossed over the lower chain. Further exploration of this discrepancy is provided in *SI Appendix*, section S6.3.

In addition to comparison with experiments, we also utilized MD to assess the impact of side chain length on contacts. 20 systems each, with side chains C1, C4, C8, and C12, were initialized and simulated in the same manner as for the C16 case. We observed that the clustering of crossing angles around the perpendicular was dependent on side chain length. As shown in *SI Appendix*, Fig. S17, medium-to-long side chains (C8, C12, C16) tended to promote a much tighter distribution of angles compared to short side chains (C1, C4). Alongside the observed dovetail pattern of side chains closest to the contact point, this demonstrates how side chains influence the geometry of contacts.

### 1.3. Contact between Chains in Thin Films.

We used CG MD to build model systems designed to reproduce the microstructure of thin films, following a procedure first described in refs. 41, and 42. This procedure starts from an initial configuration of CP chains immersed in solvent and replicates the drying of the film over several steps. Three models produced in this way are compared in the following sections. The first is composed of C16-IDTBT 24mer chains, the second of C16-IDTBT 12mer chains, and the third of regioregular poly-3-hexilthiophene (P3HT) 48mer chains. A fourth model, composed of poly[(5,6-difluoro-2,1,3-benzothiadiazol-4,7-diyl)-alt-(3,3‴-di(2-octyldodecyl)-2,2′;5′,2″;5″,2‴-quaterthiophen-5,5‴-diyl)] (PffBT4T-2OD) was also created, with results shown in *SI Appendix*. Further details of these systems are provided in *SI Appendix*, Table S3 and section S7. The C16-IDTBT 12mers and P3HT 48mers have approximately equal contour lengths, allowing for straightforward comparison between their microstructures (Section 1.3.1) and transport properties (Section 1.4). Using the procedure described in ref. 43, these films were backmapped to recover atomistic detail.

To facilitate analysis of interchain short contacts, we searched through the backmapped models to identify instances where π-system atoms on different chains were separated by 8 Å or less, to create a list of pairs of contacting chains. From each chain pair, we then identified the single, or multiple, contact locations shared between the two chains. These locations were used to generate a series of contact structures by slicing out short segments of the two chains, centered on the contact location. Segment lengths of approximately 3.2 nm were used for all CPs, corresponding to two repeat units for IDTBT and PffBT4T-2OD, and eight repeat units for P3HT. Further details about the identification and extraction of contacts may be found in *SI Appendix*, section S7.3.

**Fig. 2.**
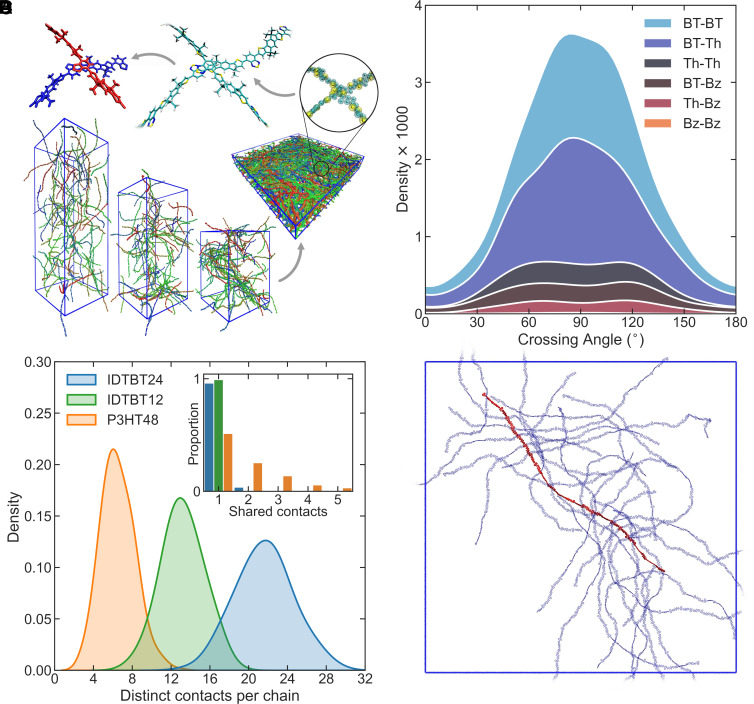
Arrangement of C16-IDTBT chains in the solid state. (*A*) Counterclockwise from *Bottom*: Snapshots of MD procedure replicating drying of a thin film. An arrow points toward the finished dry film. Chains are given randomized colors, and solvent molecules and side chains are hidden, for clarity. A zoomed-in view of a pair of coarse-grained chains is shown. An arrow points from there to a view of the same chains after backmapping. Another arrow shows the extraction of 2mer segments closest to contact point. Side chains are shortened to methyl groups as part of the final step but are hidden or shortened across all steps for clarity. (*B*) Stacked distributions of crossing angles for all interchain close contacts extracted from the C16-IDTBT 24mer system. Colors represent which moieties are in closest contact between the two backbones. Areas of colored regions represent occurrence of different moieties at the closest point of contact, with larger areas indicating more contacts of this type. (*C*) Distributions of the number of contacts to distinct chains made by each chain belonging to the different CP systems. The *Inset* shows how many contacts tend to be shared between chain pairs with at least one contact. (*D*) A visual depiction of the interconnectivity of the C16-IDTBT 24mer system. A single chain backbone is shown in red, with all chain backbones in close contact with this primary chain in semitransparent blue. The simulation bounding box is shown as a blue square.

Fig. 2*A* gives a schematic overview of the steps described in this section, including sequential snapshots of the drying film (with solvent molecules hidden), backmapping to recover atomistic detail, identification of chain pairs, and extraction of contact structures.

#### 1.3.1. Microstructure analysis.

For the analysis described in this subsection, we considered only close contacts. We define close contacts as those with a minimum interchain separation of less than 6 Å between the center-of-geometry of rings making up the backbones. These contacts are the most likely to support large electronic coupling and are thus most relevant to charge transport. Fig. 2*B* shows the distribution of crossing angles between backbones at contact locations in the C16-IDTBT 24mer system. In agreement with the 2D case, there is a clear preference for chains to cross at near-perpendicular angles. 62% of close contacts featured a crossing angle in the 90° ± 30° range. P3HT and PffBT4T-2OD show a preference for more parallel contacts (*SI Appendix*, Fig. S21*B*). Only 10% of P3HT and 9% of PffBT4T-OD close contacts feature crossing angles in this range.

Within the solid-state microstructure, we observe that C16-IDTBT chains tend to form contacts with an unusually high number of neighboring chains. Fig. 2*C* shows the distributions of contacts to distinct chains for the two C16-IDTBT systems and the P3HT system. We describe this property of contacting many neighbors as the interconnectedness of the transport network. We use the average number of close contacts with distinct chains divided by the contour length of chains to define a value expressing the interconnectivity of the system. In other words, this value gives the average number of distinct contacts per unit of backbone length. For the C16-IDTBT 24mer system this value is 0.56 nm^−1^, in the 12mer system it is 0.67 nm^−1^, and in the P3HT system it is 0.34 nm^−1^. For the C16-IDTBT systems, this corresponds to approximately one contact to a distinct chain for every monomer length. Fig. 2*D* shows the extent of this interconnectedness in the C16-IDTBT 24mer system as it applies to one example chain. This high level of interconnectedness is achieved despite the far larger and more numerous side chains attached to the C16-IDTBT polymer compared to P3HT, which one would naively expect to sterically block contact between backbones.

We note that the P3HT 48mer and C16-IDTBT 12mer systems show similar numbers of total (as opposed to distinct) contacts per chain (*SI Appendix*, Fig. S22). In P3HT, multiple contacts tend to be shared between chains due to their parallel packing arrangement, whereas in C16-IDTBT, each contact is with a new and distinct chain. This can be seen by the *Inset* in Fig. 2*C*, which displays the number of contacts shared between chains in contact.

In these two aspects (preference for near-perpendicular contacts, and large numbers of different close neighbors per chain), C16-IDTBT appears to be unique compared to other CP systems examined in a similar manner. We posit that the interconnectedness of the C16-IDTBT microstructure arises because of a combination of a stiff backbone and the promotion of perpendicular contacts. When in a crossing arrangement, many neighboring chains can be positioned alongside each other, all making close contact with the same primary chain. This is in contrast with CP systems in which parallel contact arrangements are favored, where each neighboring chain can sterically block a larger number of other potential neighbors from access to the primary chain.

Like the 2D MD simulations, Fig. 2*B* demonstrates the preference for contacts involving BT units in our model system. In the 3D case, BT–Th contacts occur most often (42%), followed by BT–BT (35%). In physical systems, as in our STM images, we expect BT–BT to be the dominant contact type. We also note that, in our modeled system, there are no instances of domains of locally aligned parallel chain segments. This contrasts with observations based on AFM and HRTEM experiments, in which such domains do sometimes appear (16, 17). We understand this discrepancy to arise due to the short timescales achievable by MD, even when adopting CG forcefields. Although crystalline-like regions of locally parallel chain segments do appear in systems of more flexible chains (e.g., P3HT) in our approach, we expect that the rigidity of the IDTBT backbone means that such arrangements cannot be formed on the timescales accessible to MD. However, we believe that arrangements of parallel chain segments are not highly relevant to the transport properties of C16-IDTBT, since it is at perpendicular contacts, and not within parallel-aligned domains, that close backbone contact occurs.

#### 1.3.2. Interactions at contacts.

**Fig. 3.**
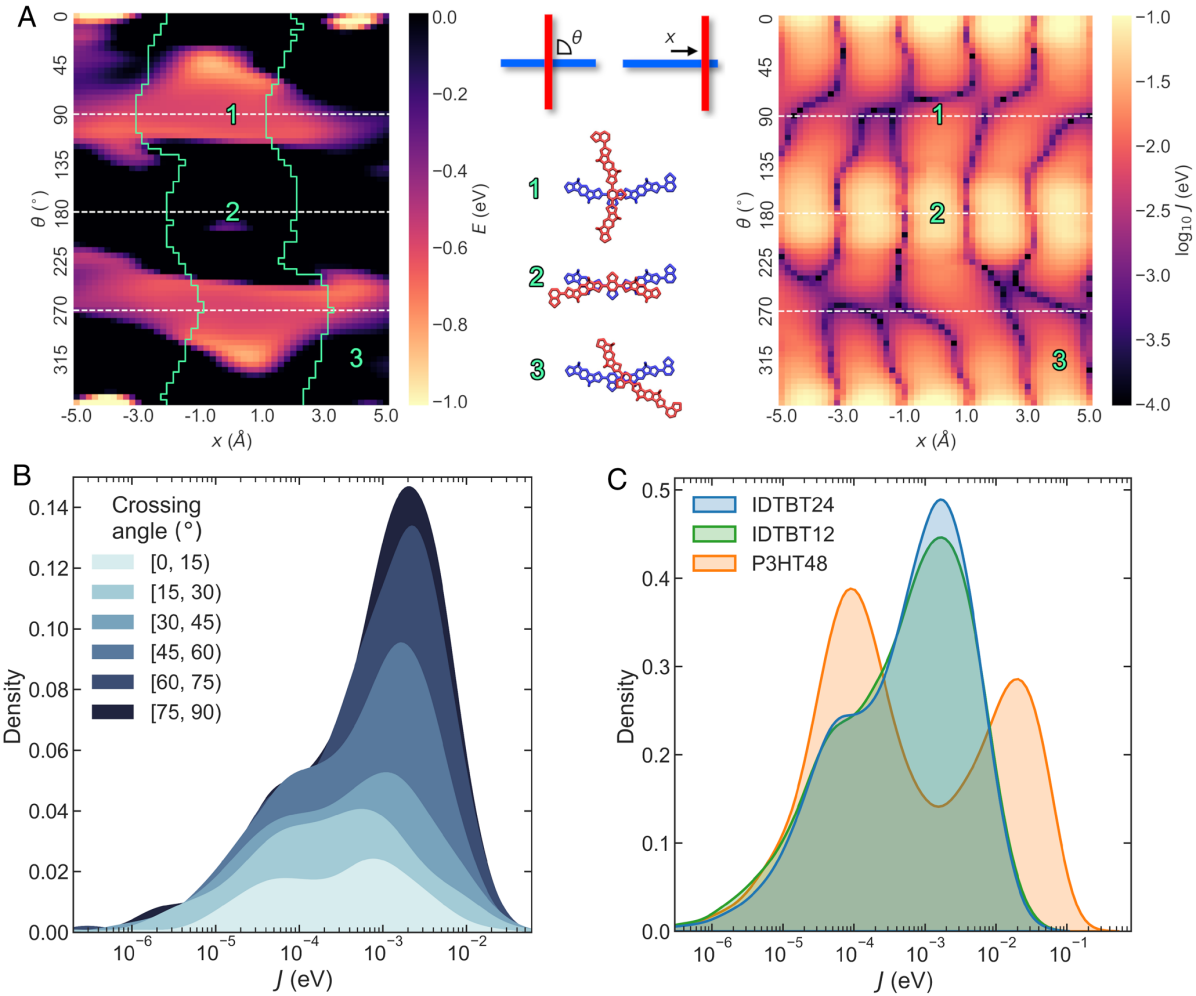
Interactions between chains. (*A*) Heatmaps displaying the enthalpic interaction energies, E, (*Left*) and hole transfer integrals, J, (*Right*) between a pair of IDTBT 2mer segments. Each pixel on the heatmaps represents a different arrangement, with the upper chain being shifted by x in the backbone direction of the lower chain and rotated by θ around its central BT unit. An explanatory diagram is shown between the heatmaps. Below are shown example arrangements, corresponding to marked locations on the heatmaps. White dashed lines represent parallel and perpendicular arrangements. The green lines on the energy heatmap mark the boundary between BT–BT contact (inside the green lines) and BT–Th contact (outside the green lines). Note that small areas where other moieties are in closest contact have been neglected. A detailed map of contact moieties can be seen in *SI Appendix*, Fig. S26. (*B*) Distribution of J for contacts extracted from the C16-IDTBT 24mer thin film model. Distributions are binned according to the crossing angle between backbones. (*C*) Distributions of J for contacts extracted from C16-IDTBT 24mer, C16-IDTBT 12mer, and P3HT 48mer models.

The plots in Fig. 3*A* show the enthalpic interaction energy and electronic coupling (*J*) between a pair of IDTBT 2mer segments placed in various face-on arrangements with a fixed separation distance of 4 Å. The large areas of negative interaction energy shown in the left-hand heatmap of Fig. 3*A* demonstrate that it is energetically favorable for backbones to arrange themselves with crossing angles near to the perpendicular, thus explaining the occurrence of these arrangements in our experimental and modeled systems. For most crossing angles outside of the stable regions around 90° and 270°, interaction energies rapidly rise above zero, which we surmise as being due to clashes between the sidegroups of the two chains. There are small regions of negative interaction energies corresponding to more parallel arrangements e.g., close to the top-right and bottom-left corners. However, we consider these to be entropically disfavored due to the small range of geometries they cover, hence explaining why parallel-arranged contacts are uncommon in the MD models and physical systems. To sample different configurations equivalently, interaction energies are calculated without performing additional optimization of the pair of chain segments after placement. We consider these conformations to be most representative of chain arrangements that occur during initial contact formation.

The overlaid green lines on the interaction energy heatmap mark the boundary between BT–BT and BT–Th contacts. The areas of negative interaction energy are broad, covering regions both within and outside these boundaries. This is also the same for *SI Appendix*, Fig. S27, which shows a similar heatmap constructed using the MD forcefield applied in our atomistic MD simulations. This suggests that BT–BT and BT–Th contacts are energetically favorable, thus explaining their appearance in our MD models.

The right-hand heatmap of Fig. 3*A* demonstrates how *J* varies with contact arrangement when backbones are held at a fixed separation distance. Here, we see that the short length of backbone contact associated with perpendicular arrangements results in overall lower *J* values compared to parallel arrangements, as evidenced by the brighter horizontal bands at 0°, 180°, and 360° compared to 90° and 270°. Interestingly however, this trend does not reappear when we consider contact arrangements extracted from the thin film model, as shown by Fig. 3*B*. For these more realistic contacts, we instead observe a subtle positive correlation between larger crossing angles and higher *J*. This occurs because backbones tend to pack more tightly at perpendicular contacts, with a modal separation between the centers of π-conjugated rings of 3.8 Å for contacts with crossing angles between 90° ± 30°, compared to 5.1 Å for crossing angles outside of this range. In P3HT, the reverse trend is seen, with the strongest coupling values achieved by contacts in parallel arrangements (*SI Appendix*, Fig. S25*B*).

Fig. 3*C* shows how the overall distribution of *J* in C16-IDTBT compares against P3HT. We can identify two peaks in each of the distributions, which correspond to 1st and 2nd nearest neighbor (NN) contacts, at larger and smaller *J* values, respectively. The increased height of the 2nd NN peak for P3HT, compared to the slight bump in the C16-IDTBT distributions, reveals the presence of more repeated packing structures in P3HT i.e., its more semicrystalline microstructure.

Despite the unusual trend of *J* increasing with crossing angle, *J* values are generally modest in C16-IDTBT, with a modal value of 2 meV for 1st NNs. By comparison, 1st NNs in P3HT are coupled with a modal value of 19 meV. By considering the distribution of crossing angles for P3HT shown in *SI Appendix*, Fig. S21*B*, and the coupling heatmap for P3HT shown in *SI Appendix*, Fig. S28, we can deduce that the larger *J* values achieved by P3HT occur because P3HT backbones are more likely to arrange themselves in a parallel fashion while maintaining similar backbone separation distances to C16-IDTBT.

Based on these results, we can surmise that fast interchain charge transfer rates are not likely to be the cause of the high hole mobility of C16-IDTBT. Rather, this property must be related to the peculiar microstructural arrangement of chains that produce a highly interconnected transport network compared to other CPs, along with other factors such as its high intrachain mobility.

Interaction energy and coupling heatmaps for pairs of P3HT chain segments may be seen in *SI Appendix*, Fig. S28 and contrasted with those shown above. Heatmaps corresponding to arrangements covering an additional degree of freedom (shifts in the y-direction i.e., perpendicular to the backbone direction of the stationary chain segment) may be seen in *SI Appendix*, Figs. S30–S32.

### 1.4. Transport Simulations.

**Fig. 4.**
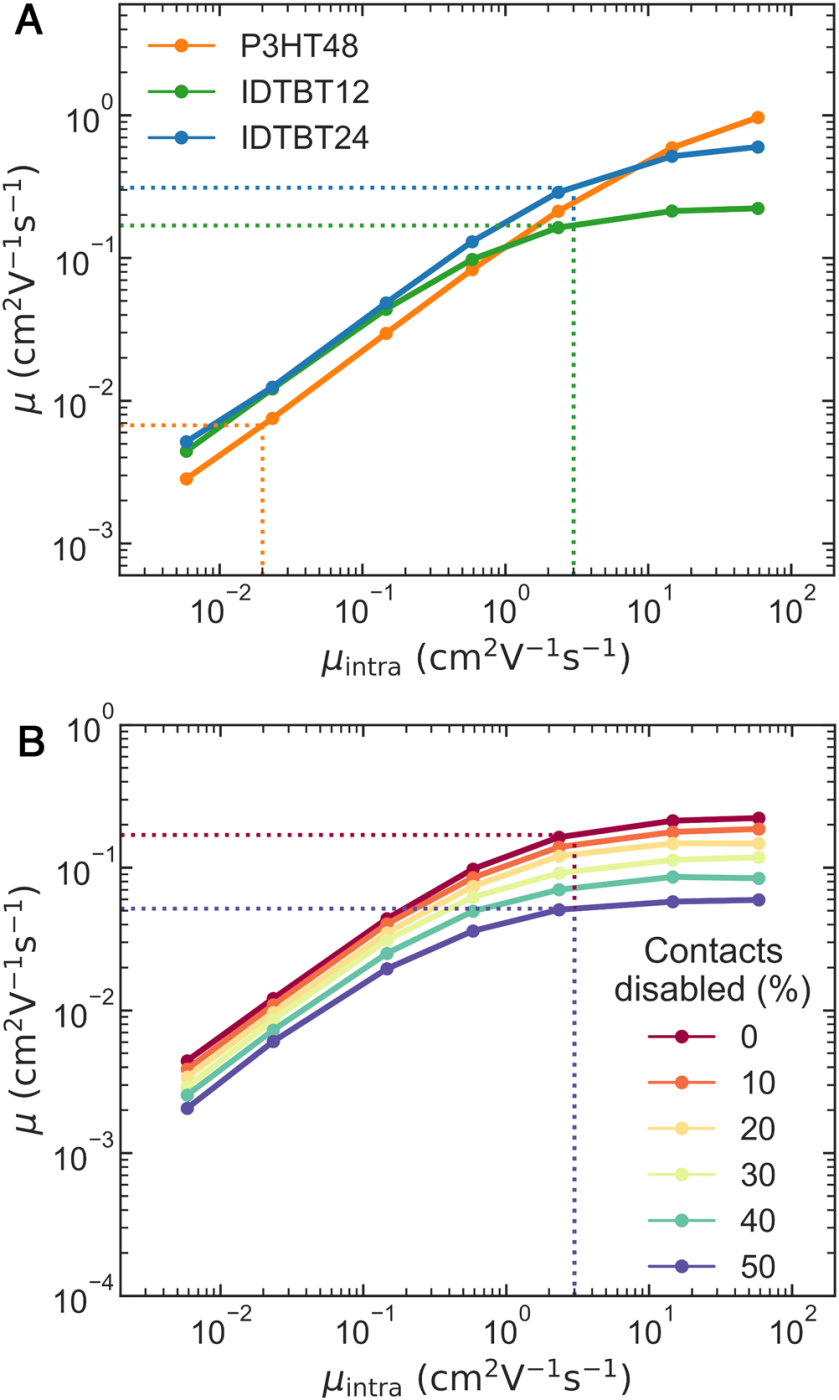
In-plane bulk mobility (*μ*) estimates from KMC simulations. *μ* is shown to vary as a function of intrachain mobility (*μ_intra_*). The dotted vertical lines indicate experimentally-informed *μ_intra_* values. (*A*) *μ* of C16-IDTBT 24mers, C16-IDTBT 12mers, and P3HT 48mers are compared. (*B*) *μ* of IDTBT 12mers. The different colored lines show how *μ* changes as increasing proportions of interchain contacts are disabled at random.

We simulated hole transport through C16-IDTBT and P3HT microstructures derived from the backmapped thin film models using an in-house KMC code (44), with charge transfer rates calculated using semiclassical Marcus theory. *J_inter_* values were calculated as described in the previous section. *J_intra_* values were varied over multiple simulations. A full description of our choice of transport-relevant electronic structure parameters [*J_intra_*, *J_inter_*, site energies (*ε*), reorganization energy (*λ*)], is provided in *SI Appendix*, section S9.2. Fig. 4*A* compares *μ* for C16-IDTBT 12mers, C16-IDTBT 24mers, and P3HT 48mers. In the limit of low external electric field, there exists an analytic relationship between *μ_intra_* and *J_intra_*, which was used to rescale the *x*-axes of the plots (*SI Appendix*, section S9.3).

Running transport simulations with a range of *μ_intra_* values allows us to nullify the main recognized advantage that C16-IDTBT has over P3HT (the high *μ_intra_* of the former) and thus isolate the influence of interconnectivity on the bulk mobility. When *μ_intra_* is low (< 1 cm^2^ V^−1^ s^−1^), both C16-IDTBT systems outperform P3HT. This indicates a situation in which the higher interconnectivity of C16-IDTBT outweighs the order-of-magnitude larger *J_inter_* values of P3HT; For transport, it is more advantageous to interact with many neighboring chains via a relatively low *J_inter_*, than a small number of chains with large *J_inter_*.

As *μ_intra_* increases beyond 1 cm^2^ V^−1^ s^−1^, the bulk mobilities of all systems begin to plateau. This is especially prominent for the C16-IDTBT systems. At this point, chain length starts to influence *μ*, with C16-IDTBT 24mers showing higher *μ* than 12mers. The higher *J_inter_* values of P3HT mean that interchain charge transfer does not act as a limiting bottleneck to transport until much higher *μ_intra_*. Thus, we observe only a slight downward bend in the trend of increasing *μ* at the highest *μ_intra_* values. *SI Appendix*, Fig. S35 shows how the mobilities of both systems could be improved by higher *J_inter_* values, with C16-IDTBT benefitting more.

The rapid increase of *μ* with *μ_intra_* across all systems, up to the point of saturation, confirms the importance of fast intrachain transport for achieving large bulk mobilities. The vertical dotted lines in Fig. 4*A* indicate likely values of *μ_intra_*. For C16-IDTBT we chose a value of 3 cm^2^ V^−1^ s^−1^. This is three times larger than the *μ_intra_* lower limit found by our PR-TRMC measurements, which follows the precedent of a previous work by Bird et al. in which they found *μ_intra_* for polyfluorene was three times larger than the mobility measured by PR-TRMC, after accounting for defects, backbone curvature, and finite chain length (45). A value of 3 cm^2^ V^−1^ s^−1^ also aligns with a theoretical prediction of *μ_intra_* for C16-IDTBT (15). For P3HT we used a *μ_intra_* value of 0.02 cm^2^ V^−1^ s^−1^ based on PR-TRMC measurements (46). With *μ_intra_* set at these experimentally-informed values, we observe values of *μ* that correspond well with bulk transport experiments. The C16-IDTBT 24mer system shows a simulated *μ* of 0.3 cm^2^ V^−1^ s^−1^, slightly lower than the 0.75 cm^2^ V^−1^ s^−1^ measured for samples with *M_w_* of 25.1 g mol^−1^ (corresponding to 19 repeat units) by FET (5). The P3HT system shows *μ* of ~0.007 cm^2^ V^−1^ s^−1^.

Fig. 4*B* makes clear the impact of interconnectivity on *μ* in the C16-IDTBT 12mer system. As increasing proportions of interchain contacts are disabled (hopping rates set to zero), *μ* decreases across all *μ_intra_*. At *μ_intra_* = 3 cm^2^ V^−1^ s^−1^, the bulk *μ* is reduced by a factor of ~3 when 50% of contacts have been disabled.

Our results reveal how a high *μ_intra_* is complemented by other factors influencing transport, including the length of chains, and the density of interchain contacts along the chain backbones.

### 1.5. Results Summary.

Our results may be summarized as follows: The C16-IDTBT backbone is rigid compared to other CPs, but chains still bend when hemmed in by other chains. Backbone contact involves chains crossing in a perpendicular fashion. Contacts predominantly involve the BT units of both chains. Side chains play a role in promoting perpendicular contacts, with longer side chains resulting in a narrower distribution of crossing angles around the perpendicular in 2D simulations. The potential decrease in *J* due to the intrinsic short length of backbone contact in a perpendicular arrangement is partially offset by the tight packing that can be achieved by chains in these configurations compared to parallel arrangements. However, coupling strengths are still modest, suggesting charge transfer rates are not unusually high. The perpendicular contact arrangement and stiff backbones allow for a large number of distinct contacts per chain in the solid state, producing a highly interconnected transport network. The large hole mobility of C16-IDTBT arises as a consequence of these microstructural features, in combination with long chains and a high intrachain mobility.

## 2. Discussion

Time-dependent fluctuations in *J* have been identified as a key influence on transport properties in numerous works (9, 47–49). Our results suggest that, in C16-IDTBT, side chains help to stabilize perpendicular contacts within a narrow range of energetically allowed molecular arrangements, which may constrain the range of such fluctuations. In Section 1.2.2, we described how this tendency to “lock” contacts into perpendicular arrangements was more pronounced with longer side chains. In Section 1.2 we also identified an unusual dovetailed pattern adopted by C16 side chains. By locking the backbones in this fashion and restricting their freedom to drift apart, slip past one another or rotate, fluctuations in *J* may be reduced. This may be quantified by considering the variability in *J* for the contact arrangements in Fig. 3*A*. When limiting to only those arrangements that are energetically favored (the bright areas in the energy heatmap), the corresponding *J*s are distributed in a relatively narrow range, with a SD of 20 meV (*SI Appendix*, Fig. S33).

In addition, the tendency to lock backbones into a perpendicular arrangement may explain why C16-IDTBT achieves larger hole mobilities than the maximally extended donor variant C16-TBIDTBT (33). When lengthening the donor unit, the spacing between side chain attachment points along the backbone increases. While this increases the space available for interchain contacts, the side chains closest to a point of contact will less tightly constrain the paired backbones, allowing for more fluctuations in the contact geometry, and therefore *J*. Furthermore, the allowance for larger rotations away from the perpendicular may limit the number of chains each chain can contact, as discussed regarding P3HT in Section 1.3.1. This suggests that there may be some optimal degree of spacing between side chain attachment points, possibly explaining the increased mobilities exhibited by the moderately extended C16-TIFBT (33).

The observation of a perpendicular contact motif appears to complement the mesoscale picture of a system of overlapping domains offered by Cendra et al. (16). It explains how, at local scales, contact may be readily achieved between chains belonging to different domains of aligned backbones. This most clearly applies to the observed peak at 90° in the distribution of domain overlap angles. However, the nanoscale view accessed by our STM images also helps to interpret microstructural trends occurring at domain boundaries with overlap angles less than perpendicular. For instance, we observed that, despite their long persistence lengths (Section 1.1), the flexibility of chains is sufficient for them to accommodate perpendicular contacts between chains in domains with nonperpendicular relative orientations (*SI Appendix*, Fig. S8). Thus, even where domains overlap at smaller angles, the microscopically stable perpendicular crossing motif appears to underpin the interchain contacts. These observations suggest that a limited degree of backbone flexibility may benefit transport. In contrast, too much backbone flexibility would reduce the end-to-end lengths of chains, making each chain less effective as a “highway” via which charges can move through the material, and will also be detrimental to intrachain mobility.

## 3. Conclusion

We have presented a combined experimental and theoretical investigation into the nature of interchain contacts in C16-IDTBT thin films, and their impact on charge transport. C16-IDTBT achieves high mobilities by combining fast intrachain transport with a highly interconnected transport network. Our results demonstrate that this latter quality arises as a consequence of an unusual interchain contact motif, in which backbones make contact while crossing-over each other at near-perpendicular angles.

We have explored the role played by side chains and backbone rigidity in the promotion of this contact motif, revealing that 1) bulky side chains are not necessarily detrimental to contact formation and can play a positive role in promoting useful contact arrangements, and 2) some degree of backbone flexibility can be beneficial in allowing chains to more easily arrange themselves into these contact motifs.

In addition to optimizing side chain length and backbone flexibility, we expect the following other areas of chemical structural modification may be worth exploring when considering the design of CPs for improved transport properties: 1) Modifying the BT unit in order to increase the π-orbital overlap at contacts, and 2) Modifying the chemistry of side chains, especially near the backbone attachment point, in order to better promote perpendicular arrangements.

This work demonstrates that understanding the impact of chemical structure on interchain contact and connectivity is of upmost importance when designing polymers for improved charge transport. Such considerations should go beyond evaluating the tendency to crystallize and related ordered packing arrangements: Understanding how chains make contact, and how this influences the geometrical properties of the transport network, is equally important in noncrystalline polymer materials.

Finally, our results demonstrate the essential role of higher-resolution techniques (STM, MD) for properly interpreting mesoscale information from other methods such as HRTEM or AFM. This is especially pertinent given the tendency for packing arrangements at the nanoscale to govern the microstructure of materials at the mesoscale and beyond, and thus control their bulk optoelectronic properties, as shown by the relationship between perpendicular contacts and the interconnectedness of the transport network in C16-IDTBT.

## 4. Methods

### 4.1. PR-TRMC.

PR-TRMC experiments were performed using a 2 MeV Van de Graaff accelerator at the Accelerator Center for Energy Research (ACER) at Brookhaven National Laboratory (BNL). Full details of the technique are given elsewhere (45, 50). Briefly, a high-energy electron pulse generates excess electrons and holes in benzene, which is contained within a microwave cavity. The benzene solutions typically contained 0.2 mM (repeat units) C16-IDTBT. Tetrafluorobenzoquinone (F4BQ) or oxygen (O2) were used as scavengers to capture the electrons, leaving the holes to be captured by C16-IDTBT. The radical anions of F4BQ or O2 make negligible contribution to the observed microwave absorption signals compared to the holes on C16-IDTBT. Extracting the real and imaginary parts of the conductivity is made possible by reconstructing the microwave cavity resonance curve as a function of time after the pulse using a transmission line model (50). Supporting pulse radiolysis experiments with UV/vis/nearIR transient absorption (TA) were performed using the 9 MeV Laser Electron Accelerator Facility (LEAF) also at BNL (51). The TA data quantified the concentration of holes on the polymer as a function of electron beam dose, enabling the microwave conductivity data to be converted to a mobility. See *SI Appendix*, section S2 for further details.

### 4.2. STM.

The sample was prepared by electrospray deposition (ESD, 4-stage Molecularspray Ltd. system) of a solution of the polymers dissolved in chlorobenzene (≈0.025 g/L) and diluted with methanol in a 4:1 volume ratio. The substrate was kept at room temperature during ESD with the deposition ion current monitored. The total deposition charges amounted to 5 pAh. A film of Au(111)/mica (Georg Albert PVD, 300 nm thickness) was used as substrate and prepared in ultrahigh vacuum (UHV) by cycles of argon sputtering and annealing to 500 °C. STM measurements were performed in UHV. C16-IDTBT was measured with a low-temperature scanning tunneling microscope (LT-STM, CreaTec Fischer & Co. GmbH), kept at −196 °C. All images were acquired in constant current feedback mode. STM images were analyzed by WSxM (52). Geometry-optimized molecular models were created with the Avogadro molecular editor using the MMFF94 force field (53) and the fitting of the images was performed using the LMAPper software (54). See *SI Appendix*, section S3 for further details.

### 4.3. MD.

All MD simulations were carried out in GROMACS 2018 (55). Parameterization details for the atomistic and CG forcefields may be found in *SI Appendix*, section S4. Full procedure details may be found in *SI Appendix*, section S5 (1D), *SI Appendix*, section S6 (2D), and *SI Appendix*, section S7 (3D). Summaries of the MD procedures are given below.

#### 4.3.1. Atomistic chains on substrate.

C16-IDTBT forcefield and Au atom type were based on the OPLS-AA package (56, 57). Two 12mer chains of C16-IDTBT were initialized above a Au(111) surface. The backbone of the first chain was placed 2 nm above the substrate, orientated along *x*-direction. The second chain was placed with its backbone 6 nm from the substrate. The upper chain was rotated around its central BT unit to point along a random direction in the *xy*-plane, and then translated in the *x*-direction by a random amount between ±3 nm. 200 repeats were run, with different rotations and *x*-shifts. All atoms were initialized with a small velocity (1 nm ns^−1^) directed toward the substrate. Production runs were performed in an NVT ensemble. System temperature was coupled to a thermal bath at 600 K for 5 ns, before being cooled, at a linear rate, to 300 K over 4 ns, after which the simulation continued at 300 K for a further 1 ns.

#### 4.3.2. CG film deposition.

C16-IDTBT, P3HT, PffBT4T-2OD, and solvent forcefields were based on the Martini 3 package (58–61). Initialization of each system involved placing CP chains randomly within a large simulation box. 300 chains were used for C16-IDTBT 24mers, 250 for C16-IDTBT 12mers, 420 for P3HT 48mers, and 280 for PffBT4T-2OD 12mers. The space between chains was then filled with solvent molecules. *x* and *y* side lengths of the box were fixed at a length larger than the contour length of the CP chains being simulated. Initial *z*-heights for each box were chosen to yield CP concentrations of 40 mg mL^−1^, which is within the range of concentrations used in the slot-die coating method of thin film manufacture.

Drying of thin films was simulated over a series of steps. At the beginning of each step, a small proportion of solvent molecules was removed at random. The structure was then equilibrated for 0.5 ns in an NVT ensemble and 4.0 ns in an NPT ensemble, followed by a production run. Each production run consisted of a 3.0 ns NPT simulation. During both NPT stages, semi-isotropic pressure coupling was enabled, applying atmospheric pressure in the *z*-direction, and causing the box to shrink in this direction. Once all solvent molecules had been removed, the dry films were subject to a final annealing step. Total simulation times were ~2 μs for each film.

### 4.4. DFT.

All DFT calculations were performed using Gaussian 16 (62) at the cam-b3lyp/cc-pVDZ level of theory, with Grimme’s D3 dispersion correction. Calculations involved pairs of chain segments i.e., dimers (2mers for C16-IDTBT, and 8mers for P3HT). To analyze contact geometries extracted from the backmapped thin film models, chains were shortened to appropriate segment lengths, centered on the monomers in closest contact, by removing all atoms belonging to more distant repeat units, and capping the remaining segments with hydrogen atoms. Side chains were replaced with methyl groups. Dimer structures were then subject to a few steps of optimization before coupling calculations were performed. The chain segments used to build interaction heatmaps were optimized individually, before being positioned as a dimer structure, with no further optimization performed after placement.

Coupling calculations were performed using the projective method described in ref. 63. This evaluates the expectation value of the electronic Hamiltonian of the dimer with respect to the highest occupied molecular orbitals of the molecules, in the frozen orbital approximation. See *SI Appendix*, section S8 for further details.

### 4.5. KMC.

Material microstructures are described by sites connected via edges, equivalent to a mathematical graph. Sites represent segments of polymer backbones, while edges represent coupling between these segments. Hole transport is modeled by the movement of a localized free charge hopping between sites. The hopping rate, $\Gamma_{\mathrm{ij}}$, between sites *i* and *j*, is computed using high-temperature Marcus theory:

[1] $$\Gamma_{\mathrm{ij}}=\frac{{\left|J_{\mathrm{ij}}\right|}^{2}}{\hbar }\sqrt{\frac{\pi }{\lambda k_{B}T}}\exp\left(-\frac{{\left(\Delta E_{\mathrm{ij}}+\lambda \right)}^{2}}{4\lambda k_{B}T}\right).$$

The influence of an external electric field on a hole is included by adjusting the energy difference between sites as follows:

[2] $$\Delta E_{\mathrm{ij}}=\varepsilon_{i}-\varepsilon_{j}+e\vec{F}\cdot {\vec{r}}_{\mathrm{ij}},$$

where *e* is the charge of an electron, $\vec{F}$ is the external field and ${\vec{r}}_{\mathrm{ij}}$ is the separation between sites. Thus, different forward and backward hopping rates are used for sites at different positions in the field direction. Field strength was set to 10^6^ V m^−1^. The temperature was set at 300 K.

A single free charge was simulated at a time. At the start of each simulation run, the charge is placed at a random site. The time-evolution of the system is propagated until a set simulation time is reached, at which point the run halts and the average velocity of the charge in the field direction, ⟨v⟩, is computed. The mobility is then calculated from

[3] $$\mu =\frac{\left\langle v\right\rangle }{F}.$$

Runs are carried out until *μ* converges. See *SI Appendix*, section S9 for further details.

## Supplementary Material

Appendix 01 (PDF)

## Data Availability

Some study data are available. Molecular dynamics forcefields and structures have been deposited in the publication "Perpendicular Crossing Chains Enable High Mobility in a Non-Crystalline Conjugated Polymer: Molecular Dynamics Forcefields and Structures" and can be accessed at https://doi.org/10.5281/zenodo.11636232 (64). Additional molecular dynamics forcefields and structures are available at https://doi.org/10.11583/DTU.c.5254236.v1 (65). The simulation software can be found at https://github.com/jfcoker/tofet (66) and https://github.com/Jenny-Nelson-Group/counterpoise-J (67). Other study data, including MD trajectories and electronic structure calculation log files, are not hosted online due to their size; however, all data are available from the authors upon request.

## References

[1] W. Zhang , Indacenodithiophene semiconducting polymers for high-performance, air-stable transistors. J. Am. Chem. Soc. **132**, 11437–11439 (2010), 10.1021/ja1049324. 20677750

[2] X. Zhang , Molecular origin of high field-effect mobility in an indacenodithiophene-benzothiadiazole copolymer. Nat. Commun. **4**, 2238 (2013), 10.1038/ncomms3238. 23900027

[3] W. Wang , Probing the intrinsic charge transport in indacenodithiophene-co-benzothiadiazole thin films. AIP Adv. **7**, 125314 (2017), 10.1063/1.5001986.

[4] Y. Zheng , An intrinsically stretchable high-performance polymer semiconductor with low crystallinity. Adv. Funct. Mater. **29**, 1905340 (2019), 10.1002/adfm.201905340.

[5] B. Zhao , Simultaneous enhancement of stretchability, strength, and mobility in ultrahigh-molecular-weight poly(indacenodithiophene-co-benzothiadiazole). Macromolecules **54**, 9896–9905 (2021), 10.1021/acs.macromol.1c01513.

[6] Y. Zheng , A molecular design approach towards elastic and multifunctional polymer electronics. Nat. Commun. **12**, 5701 (2021), 10.1038/s41467-021-25719-9. 34588448 PMC8481247

[7] J. Mun , A design strategy for high mobility stretchable polymer semiconductors. Nat. Commun. **12**, 3572 (2021), 10.1038/s41467-021-23798-2. 34117254 PMC8196107

[8] H.-C. Wu , Highly stretchable polymer semiconductor thin films with multi-modal energy dissipation and high relative stretchability. Nat. Commun. **14**, 8382 (2023), 10.1038/s41467-023-44099-w. 38104194 PMC10725446

[9] S. Fratini, M. Nikolka, A. Salleo, G. Schweicher, H. Sirringhaus, Charge transport in high-mobility conjugated polymers and molecular semiconductors. Nat. Mater. **19**, 491–502 (2020), 10.1038/s41563-020-0647-2. 32296138

[10] R. Noriega, Efficient charge transport in disordered conjugated polymer microstructures. Macromol. Rapid Commun. **39**, 1800096 (2018), 10.1002/marc.201800096. 29682841

[11] R. Noriega , A general relationship between disorder, aggregation and charge transport in conjugated polymers. Nat. Mater. **12**, 1038–1044 (2013), 10.1038/nmat3722. 23913173

[12] D. T. Duong, M. F. Toney, A. Salleo, Role of confinement and aggregation in charge transport in semicrystalline polythiophene thin films. Phys. Rev. B **86**, 205205 (2012), 10.1103/PhysRevB.86.205205.

[13] D. Venkateshvaran , Approaching disorder-free transport in high-mobility conjugated polymers. Nature **515**, 384–388 (2014), 10.1038/nature13854. 25383522

[14] J. Kirkpatrick , A systematic approach to the design optimization of light-absorbing indenofluorene polymers for organic photovoltaics. Adv. Energy Mater. **2**, 260–265 (2012), 10.1002/aenm.201100622.

[15] R. Dilmurat, S. Prodhan, L. Wang, D. Beljonne, Thermally activated intra-chain charge transport in high charge-carrier mobility copolymers. J. Chem. Phys. **156**, 084115 (2022), 10.1063/5.0082569. 35232178

[16] C. Cendra , Unraveling the unconventional order of a high-mobility indacenodithiophene-benzothiadiazole copolymer. ACS Macro Lett. **10**, 1306–1314 (2021), 10.1021/acsmacrolett.1c00547. 35549036

[17] I. Dobryden , Dynamic self-stabilization in the electronic and nanomechanical properties of an organic polymer semiconductor. Nat. Commun. **13**, 3076 (2022), 10.1038/s41467-022-30801-x. 35654891 PMC9163058

[18] H. Makki, C. A. Burke, A. Troisi, Microstructural model of indacenodithiophene-co-benzothiadiazole polymer: Π-crossing interactions and their potential impact on charge transport. J. Phys. Chem. Lett. **14**, 8867–8873 (2023), 10.1021/acs.jpclett.3c02305. 37756473 PMC10561260

[19] C. J. Takacs, M. A. Brady, N. D. Treat, E. J. Kramer, M. L. Chabinyc, Quadrites and crossed-chain crystal structures in polymer semiconductors. Nano Lett. **14**, 3096–3101 (2014), 10.1021/nl500150t. 24820648

[20] G. L. Schulz , The PCPDTBT family: Correlations between chemical structure, polymorphism, and device performance. Macromolecules **50**, 1402–1414 (2017), 10.1021/acs.macromol.6b01698.

[21] B. M. Savoie , Mesoscale molecular network formation in amorphous organic materials. Proc. Natl. Acad. Sci. U.S.A. **111**, 10055–10060 (2014), 10.1073/pnas.1409514111. 24982179 PMC4104918

[22] N. E. Jackson, B. M. Savoie, L. X. Chen, M. A. Ratner, A simple index for characterizing charge transport in molecular materials. J. Phys. Chem. Lett. **6**, 1018–1021 (2015), 10.1021/acs.jpclett.5b00135. 26262862

[23] N. E. Jackson, L. X. Chen, M. A. Ratner, Charge transport network dynamics in molecular aggregates. Proc. Natl. Acad. Sci. U.S.A. **113**, 8595–8600 (2016), 10.1073/pnas.1601915113. 27439871 PMC4978304

[24] S. Himmelberger, K. Vandewal, Z. Fei, M. Heeney, A. Salleo, Role of molecular weight distribution on charge transport in semiconducting polymers. Macromolecules **47**, 7151–7157 (2014), 10.1021/ma501508j.

[25] K. Gu , Assessing the huang-brown description of tie chains for charge transport in conjugated polymers. ACS Macro Lett. **7**, 1333–1338 (2018), 10.1021/acsmacrolett.8b00626. 35651239 PMC10478409

[26] Y.-Y. Zhou , Visualizing the multi-level assembly structures of conjugated molecular systems with chain-length dependent behavior. Nat. Commun. **14**, 3340 (2023), 10.1038/s41467-023-39133-w. 37286537 PMC10247739

[27] A. Troisi, G. Orlandi, J. E. Anthony, Electronic interactions and thermal disorder in molecular crystals containing cofacial pentacene units. Chem. Mater. **17**, 5024–5031 (2005), 10.1021/cm051150h.

[28] X. Feng , Towards high charge-carrier mobilities by rational design of the shape and periphery of discotics. Nat. Mater. **8**, 421–426 (2009), 10.1038/nmat2427. 19363476

[29] T. H. Thomas , Short contacts between chains enhancing luminescence quantum yields and carrier mobilities in conjugated copolymers. Nat. Commun. **10**, 2614 (2019), 10.1038/s41467-019-10277-y. 31197152 PMC6565747

[30] T. H. Thomas , Chain coupling and luminescence in high-mobility, low-disorder conjugated polymers. ACS Nano **13**, 13716–13727 (2019), 10.1021/acsnano.9b07147. 31738516

[31] H. N. Tsao , Ultrahigh mobility in polymer field-effect transistors by design. J. Am. Chem. Soc. **133**, 2605–2612 (2011), 10.1021/ja108861q. 21291267

[32] H. Bronstein , Indacenodithiophene-*co*-benzothiadiazole copolymers for high performance solar cells or transistors via alkyl chain optimization. Macromolecules **44**, 6649–6652 (2011), 10.1021/ma201158d.

[33] A. Wadsworth , Modification of indacenodithiophene-based polymers and its impact on charge carrier mobility in organic thin-film transistors. J. Am. Chem. Soc. **142**, 652–664 (2020), 10.1021/jacs.9b09374. 31851506

[34] H. Chen , The effect of ring expansion in thienobenzo[b]indacenodithiophene polymers for organic field-effect transistors. J. Am. Chem. Soc. **141**, 18806–18813 (2019), 10.1021/jacs.9b09367. 31613619

[35] R. Dilmurat, V. Lemaur, Y. Olivier, S. M. Gali, D. Beljonne, Tuning short contacts between polymer chains to enhance charge transport in amorphous donor-acceptor polymers. J. Phys. Chem. C **126**, 3118–3126 (2022), 10.1021/acs.jpcc.1c09711.

[36] J. F. Ponder Jr. , Low-defect, high molecular weight indacenodithiophene (IDT) polymers via a C-H activation: Evaluation of a simpler and greener approach to organic electronic materials. ACS Mater. Lett. **3**, 1503–1512 (2021), 10.1021/acsmaterialslett.1c00478.

[37] D. A. Warr , Sequencing conjugated polymers by eye. Sci. Adv. **4**, eaas9543 (2018), 10.1126/sciadv.aas9543. 29922716 PMC6003723

[38] M. Xiao , Anisotropy of charge transport in a uniaxially aligned fused electron-deficient polymer processed by solution shear coating. Adv. Mater. **32**, 2000063 (2020), 10.1002/adma.202000063. 32363687

[39] S. Moro , The effect of glycol side chains on the assembly and microstructure of conjugated polymers. ACS Nano **16**, 21303–21314 (2022), 10.1021/acsnano.2c09464. 36516000 PMC9798861

[40] S. Moro , Molecular-scale imaging enables direct visualization of molecular defects and chain structure of conjugated polymers. ACS Nano **18**, 11655–11664 (2024), 10.1021/acsnano.3c10842. 38652866 PMC11080458

[41] R. Alessandri, J. J. Uusitalo, A. H. de Vries, R. W. A. Havenith, S. J. Marrink, Bulk heterojunction morphologies with atomistic resolution from coarse-grain solvent evaporation simulations. J. Am. Chem. Soc. **139**, 3697–3705 (2017), 10.1021/jacs.6b11717. 28209056 PMC5355903

[42] A. S. Gertsen, M. K. Sørensen, J. W. Andreasen, Nanostructure of organic semiconductor thin films: Molecular dynamics modeling with solvent evaporation. Phys. Rev. Mater. **4**, 075405 (2020), 10.1103/PhysRevMaterials.4.075405.

[43] T. A. Wassenaar, K. Pluhackova, R. A. Böckmann, S. J. Marrink, D. P. Tieleman, Going backward: A flexible geometric approach to reverse transformation from coarse grained to atomistic models. J. Chem. Theory Comput. **10**, 676–690 (2014), 10.1021/ct400617g. 26580045

[44] J. J. Kwiatkowski, J. M. Frost, J. Nelson, The effect of morphology on electron field-effect mobility in disordered C60 thin films. Nano Lett. **9**, 1085–1090 (2009), 10.1021/nl803504q. 19275245

[45] M. J. Bird , Mobility of holes in oligo- and polyfluorenes of defined lengths. J. Phys. Chem. C **118**, 6100–6109 (2014), 10.1021/jp5010874.

[46] F. C. Grozema , Hole conduction along molecular wires: σ-bonded silicon versus π-bond-conjugated carbon. Adv. Mater. **14**, 228–231 (2002), 10.1002/1521-4095(20020205)14:3<228::AID-ADMA228>3.0.CO;2-3.

[47] H. Sirringhaus, T. Sakanoue, J.-F. Chang, Charge-transport physics of high-mobility molecular semiconductors. Phys. Status Solidi (b) **249**, 1655–1676 (2012), 10.1002/pssb.201248143.

[48] S. Fratini, D. Mayou, S. Ciuchi, The transient localization scenario for charge transport in crystalline organic materials. Adv. Funct. Mater. **26**, 2292–2315 (2016), 10.1002/adfm.201502386.

[49] T. Nematiaram, A. Troisi, Strategies to reduce the dynamic disorder in molecular semiconductors. Mater. Horiz. **7**, 2922–2928 (2020), 10.1039/D0MH01159B.

[50] M. J. Bird, J. Bakalis, S. Asaoka, H. Sirringhaus, J. R. Miller, Fast holes, slow electrons, and medium control of polaron size and mobility in the DA polymer F8BT. J. Phys. Chem. C **121**, 15597–15609 (2017), 10.1021/acs.jpcc.7b04602.

[51] J. F. Wishart, A. R. Cook, J. R. Miller, The LEAF picosecond pulse radiolysis facility at Brookhaven national laboratory. Rev. Sci. Instrum. **75**, 4359–4366 (2004), 10.1063/1.1807004.

[52] I. Horcas , WSXM: A software for scanning probe microscopy and a tool for nanotechnology. Rev. Sci. Instrum. **78**, 013705 (2007), 10.1063/1.2432410. 17503926

[53] M. D. Hanwell , Avogadro: An advanced semantic chemical editor, visualization, and analysis platform. J. Cheminform. **4**, 17 (2012), 10.1186/1758-2946-4-17. 22889332 PMC3542060

[54] L. M. A. Perdigão, LMAPper–The SPM and mol viewer. SourceForge. https://sourceforge.net/projects/spm-and-mol-viewer/. Accessed 19 December 2023.

[55] M. J. Abraham , GROMACS: High performance molecular simulations through multi-level parallelism from laptops to supercomputers. SoftwareX **1–2**, 19–25 (2015), 10.1016/j.softx.2015.06.001.

[56] W. L. Jorgensen, D. S. Maxwell, J. Tirado-Rives, Development and testing of the OPLS all-atom force field on conformational energetics and properties of organic liquids. J. Am. Chem. Soc. **118**, 11225–11236 (1996), 10.1021/ja9621760.

[57] G. A. Kaminski, R. A. Friesner, J. Tirado-Rives, W. L. Jorgensen, Evaluation and reparametrization of the OPLS-AA force field for proteins via comparison with accurate quantum chemical calculations on peptides. J. Phys. Chem. B **105**, 6474–6487 (2001), 10.1021/jp003919d.

[58] R. Alessandri , Martini 3 coarse-grained force field: Small molecules. Adv. Theory Simul. **5**, 2100391 (2022), 10.1002/adts.202100391.

[59] R. Alessandri, F. Grünewald, S. J. Marrink, The Martini model in materials science. Adv. Mater. **33**, 2008635 (2021), 10.1002/adma.202008635. PMC11468591 33956373

[60] G. Rossi, L. Monticelli, S. R. Puisto, I. Vattulainen, T. Ala-Nissila, Coarse-graining polymers with the MARTINI force-field: Polystyrene as a benchmark case. Soft Matter **7**, 698–708 (2011), 10.1039/C0SM00481B.

[61] P. C. T. Souza , Martini 3: A general purpose force field for coarse-grained molecular dynamics. Nat. Methods **18**, 382–388 (2021), 10.1038/s41592-021-01098-3. 33782607 PMC12554258

[62] M. J. Frisch , Gaussian 16 Revision C.01 (Gaussian Inc., Wallingford, CT, 2016).

[63] B. Baumeier, J. Kirkpatrick, D. Andrienko, Density-functional based determination of intermolecular charge transfer properties for large-scale morphologies. Phys. Chem. Chem. Phys. **12**, 11103 (2010), 10.1039/c002337j. 20689881

[64] A. S. Gertsen, J. F. Coker, J. Nelson, Perpendicular Crossing Chains Enable High Mobility in a Non-Crystalline Conjugated Polymer: Molecular Dynamics Forcefields and Structures. Zenodo. 10.5281/zenodo.11636232. Deposited 13 June 2024.

[65] A. S. Gertsen, Coarse-grained MARTINI force fields. Technical University of Denmark. 10.11583/DTU.13603631.v1. Deposited 19 January 2021.

[66] J. J. Kwiatkowski, J. F. Coker, ToFeT. Github. https://github.com/jfcoker/tofet. Accessed 14 August 2024.

[67] J. Kirkpatrick, Counterpoise-J. Github. https://github.com/Jenny-Nelson-Group/counterpoise-J. Accessed 14 August 2024.

