# Supplementary material for: Perpendicular crossing chains enable high mobility in a noncrystalline conjugated polymer
Source: Proc Natl Acad Sci U S A. 2024 Sep 3;121(37):e2403879121. doi: 10.1073/pnas.2403879121 (PMC11406284; doi:10.1073/pnas.2403879121)
Supplement: Supplementary file 1 — Appendix 01 (PDF) [file pnas.2403879121.sapp.pdf]

Supporting Information:

Perpendicular Crossing Chains Enable High Mobility in a Noncrystalline Conjugated Polymer

*Jack F. Coker, Stefania Moro, Anders S. Gertsen, Xingyuan Shi, Drew Pearce, Martin P. van der Schelling, Yucheng Xu, Weimin Zhang, Jens W. Andreasen, Chad R. Snyder, Lee J. Richter, Matthew J. Bird, Iain McCulloch, Giovanni Costantini, Jarvist M. Frost and Jenny Nelson**

*Corresponding Author: jenny.nelson@imperial.ac.uk

Table of Contents

Supporting Figures 3
Supporting Tables 24
Section S1: Small Angle Neutron Scattering 25
Section S2: Pulse Radiolysis Time-Resolved Microwave Conductivity 27
Section S3: Scanning Tunnelling Microscopy 27
3.1. Imaging and Fitting Procedure 27
3.2. Backbones at Crossing Points 28
Section S4: Forcefield Parameterization 29
4.1. Atomistic Forcefield 29
4.2. Coarse-Grained Forcefield 29
Section S5: Persistence Length Calculation from Molecular Dynamics 31
Section S6: Molecular Dynamics Simulation of Chains on Surfaces 32
6.1. Procedure Details 32
6.2. Crossing Angle Definition 32
6.3. S and T Stacked Backbones 32
Section S7: Molecular Dynamics Simulation of Solid-State Microstructure 33
7.1. Procedure Details 33
7.2. Backmapping 33
7.3. Identifying Interchain Contacts 34
Section S8: Contact Interactions 35
8.1. Coupling Calculations 35
Section S9: Transport Simulations 35
9.1. Assigning Sites and Edges 35
9.2. Electronic Structure Parameters 36
9.3. Relationship between μ_{intra} and J_{intra} 36
9.4. Calculation of λ 37
References 38

Supporting Figures

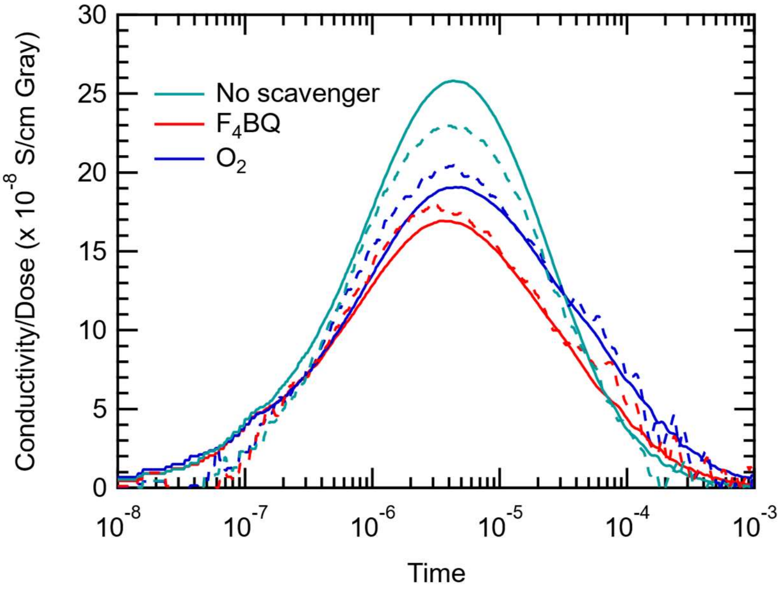

Figure S1: *Time evolution of microwave conductivity. The real (solid) and imaginary (dashed) microwave conductivity (8.73 GHz) per unit dose from pulse radiolysis are shown as a function of time after the electron pulse. Conductivity data is shown for solutions of 0.2 mM (PRU) IDTBT in benzene with no scavenger, argon-saturated (teal), 0.2 mM F_4BQ, argon-saturated (red) and O_2-saturated (blue). The signal grows as solvent radical cations transfer holes to the polymer and then decays as the IDTBT radical cations recombine with the excess negative charges that could be IDTBT, F_4BQ or O_2 radical anions depending on the solution. The additional conductivity in the absence of electron-scavengers is due to electrons on the IDTBT. There are likely to be less IDTBT radical anions than cations due to solvent impurities. The exact concentration of IDTBT anions is difficult to determine spectroscopically due to challenges in finding scavengers that act only on IDTBT holes and triplet excited states, which have overlapping absorptions with the radical anion.*

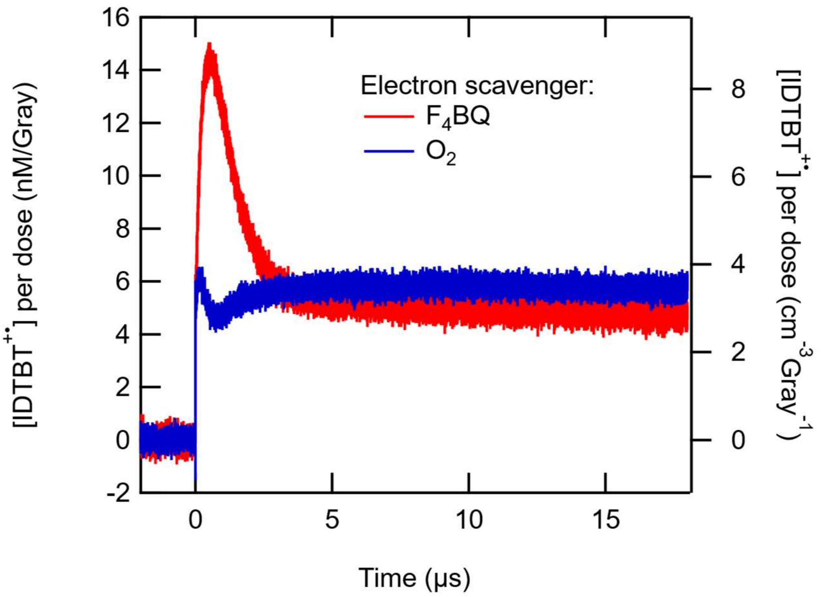

Figure S2: *Transient absorption data from pulse radiolysis. Converted to concentration of IDTBT radical cations per unit dose using the same solutions from **Figure S1**. Note that overlapping absorptions from triplet excited states contribute at this wavelength, giving a false concentration estimate in the first 5 microseconds. The absorption was measured at 1050 nm, where the IDTBT radical cation peaks, and an extinction coefficient of 96,100 $M^{-1}c^{-1}$ was used to convert to concentration. This value is based on pulse radiolysis hole transfer experiments in 1,2-dichloroethane using tritolylamine radical cation as a reference.*

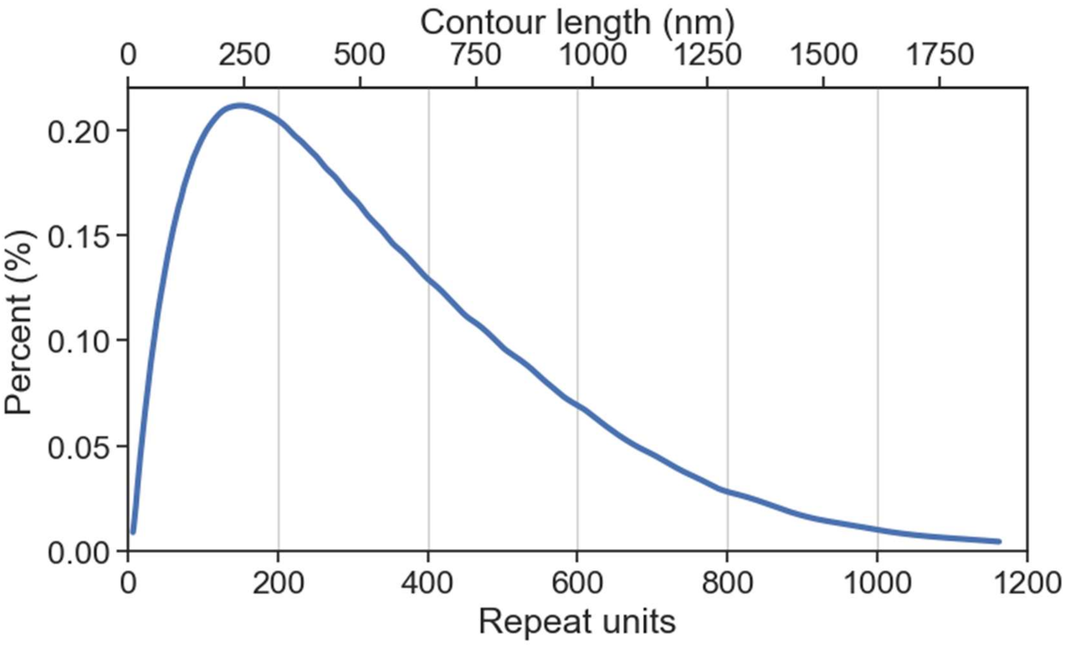

Figure S3: *Length distribution of C16-IDTBT chains in sample probed by pulse radiolysis time-resolved microwave conductivity. Molecular weight distribution was found using gel permeation chromatography (GPC) and converted to repeat units and contour length based on the molecular weight (1296.22 g mol^{-1}) and length of a monomer (1.6 nm). Note that no correction was applied to the GPC data, although there is evidence that GPC can overestimate molecular weights, as briefly discussed in **Section S1**.*

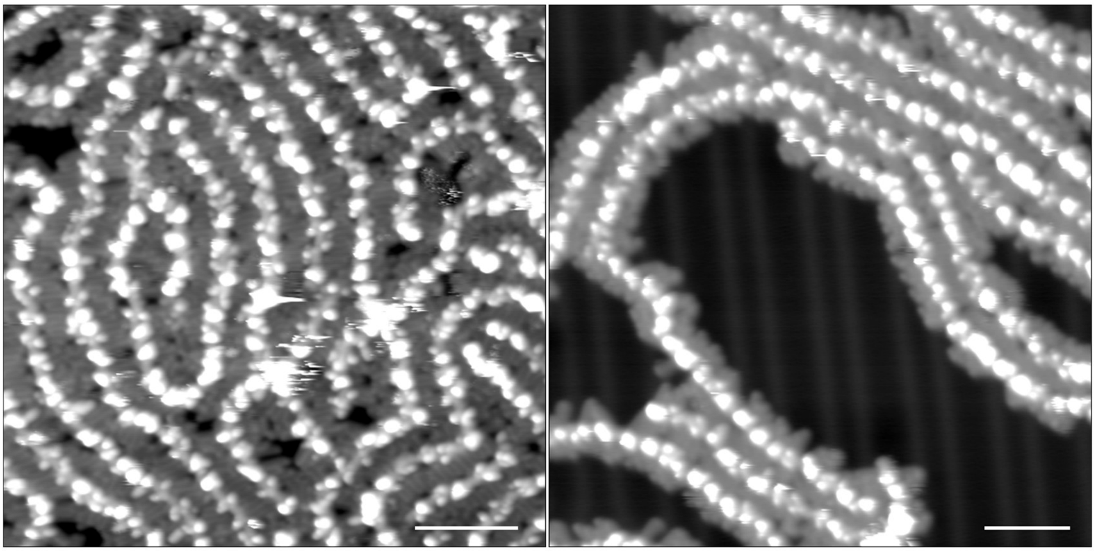

Figure S4: *Examples of self-assembled C16-IDTBT polymers, as captured in scanning tunnelling microscopy images. Polymer backbones are seen as a sequence of bright dots, caused by the two nonplanar sp*^{3} *bridging quaternary carbons in the IDT units. The sidechains appear as darker features interdigitating in the space between the backbones. Scale bars correspond to 8 nm. The scanning parameters were 80 pA, 0.70 V and 70 pA, 0.6 V for the left and right images respectively.*

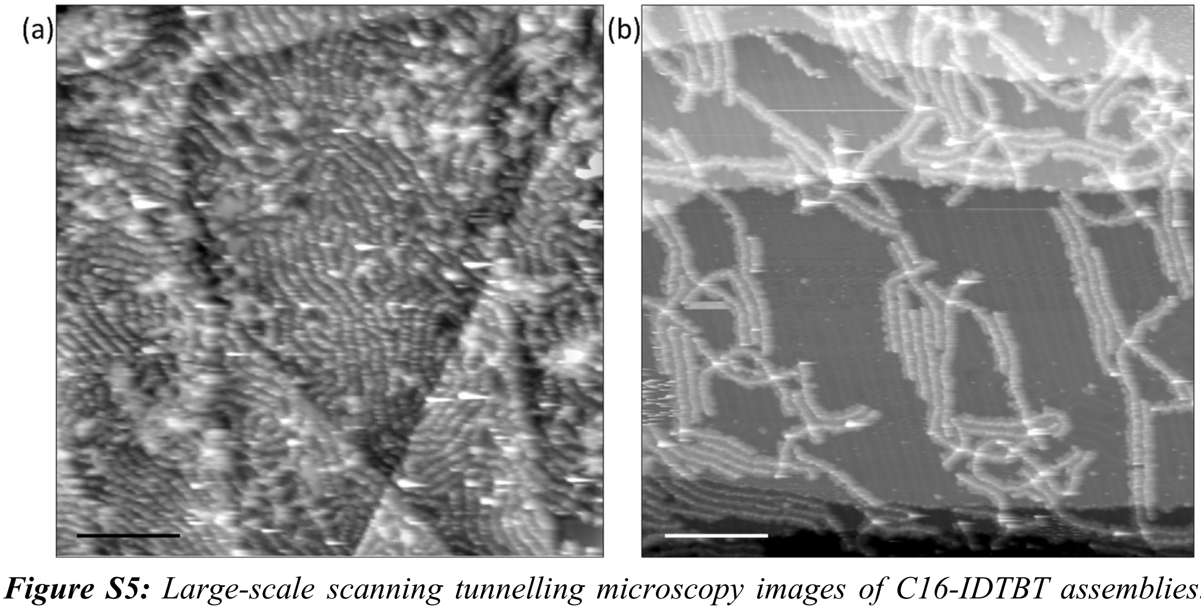

Figure S5: *Large-scale scanning tunnelling microscopy images of C16-IDTBT assemblies. Subfigures show regions of (a) high-density and (b) low-density coverage. Even at low coverages, the polymers are seen to interact with each other, either laterally by interdigitation of their side chains or through crossings of the backbones, creating extended low-density polymer networks. Scale bars correspond to 25 nm in (a) and 30 nm in (b). Scanning parameters were a) 100 pA, 0.80 V, and b) 65 pA, 1.4 V.*

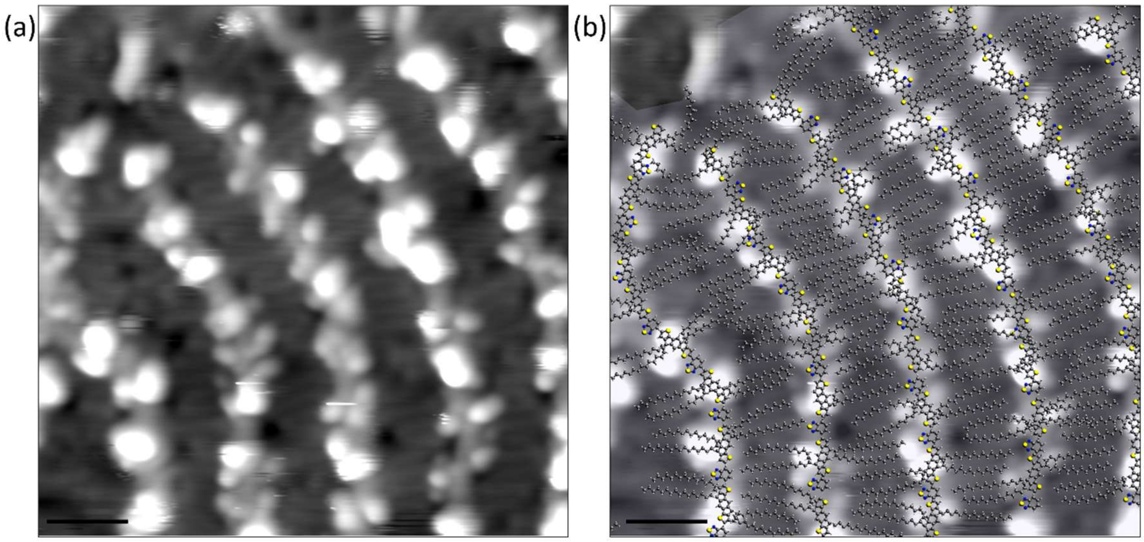

Figure S6: *High-resolution scanning tunnelling microscopy (STM) images of C16-IDTBT adsorbed on Au(111). (a) The polymer backbones appear as periodic sequences of bright features that are associated with the sp^3-hybridised carbon atoms of the IDT units. Side chains can be distinguished protruding from the backbones, and are seen to interdigitate with the side chains of neighbouring polymers. (b) Geometry-optimized molecular models are overlayed on the STM image in (a). While the orientation of the IDT units is unequivocally determined by the position of the side chains, the orientation of the BT units cannot be gleaned directly from the STM images. BT units have been added to the fit for completeness, but their orientation has been randomly assigned. Scale bars correspond to 3 nm. Scanning parameters were 80 pA, 0.70 V.*

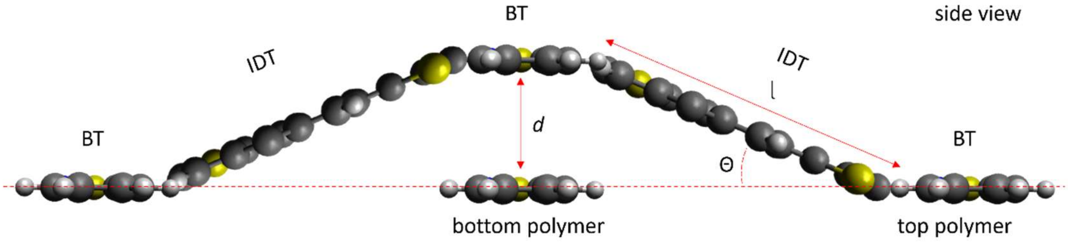

Figure S7: *Side view of the molecular models created to fit the 3D crossing points. To better represent the scanning tunnelling microscopy (STM) images, the 3D nature of the crossing between polymers needs to be considered. The fitting procedure introduced for planar backbones leads to insufficient space for the BT units. Since STM images display the normal projection of molecules onto the surface plane, IDT units have been tilted out of the surface by an angle Θ that takes into consideration the π-stacking distance d of IDTBT and the length l of the IDT comonomer. Normal projections of these models have been used to fit the STM images, resulting in a much better match.*

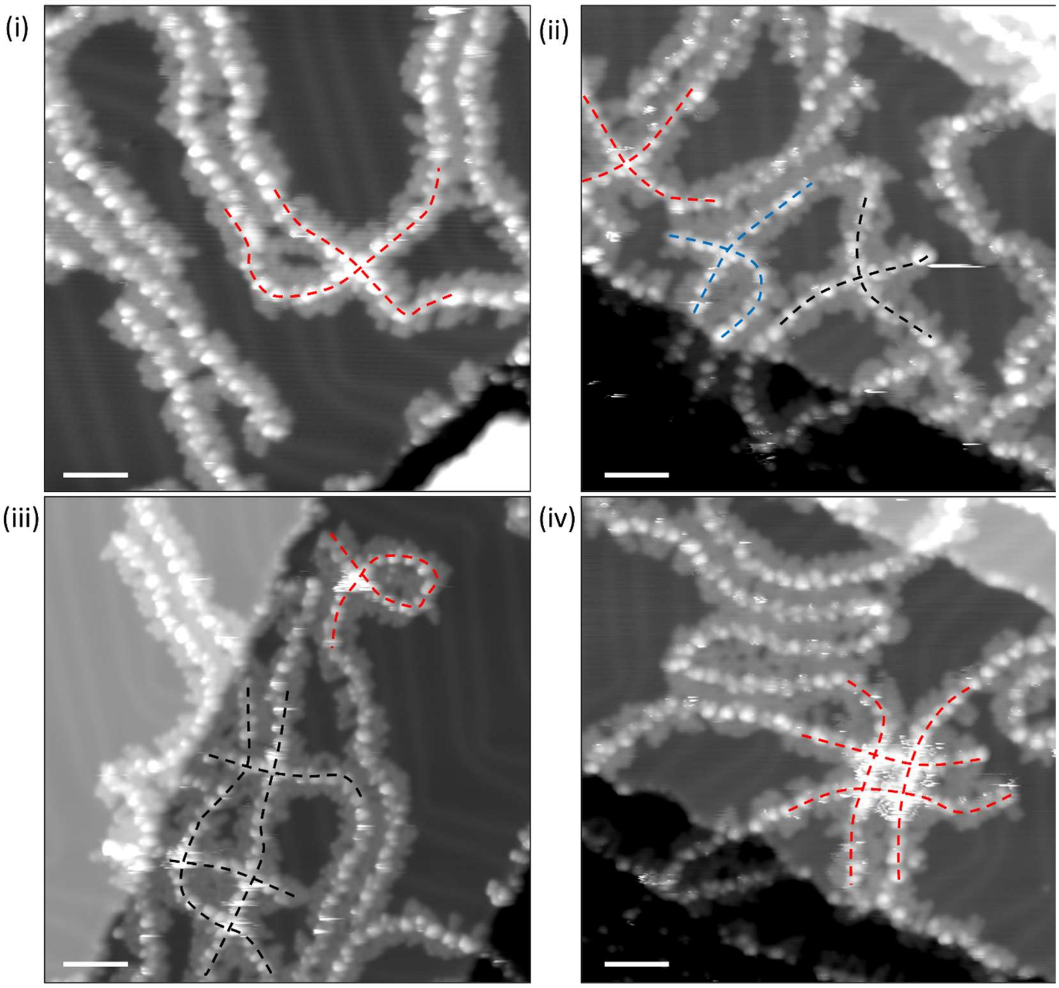

Figure S8: *Crossing points in scanning tunnelling microscopy (STM) images. Backbones are seen to form a ~90° angle at the point of contact. However, away from the point of contact, they can quickly diverge into different orientations. This explains how 90° crossing points at local scales can still be compatible with different relative orientations between domains seen in transmission electron microscopy analysis*[1]*. Scale bars correspond to 7 nm in (i), 9 nm in (iii) and 8 nm in (ii) and (iv). Scanning parameters were i) 70 pA, 0.90 V, ii) 150 pA, 1.1 V, iii) 90 pA, 0.60 V, and iv) 150 pA, 1.1 V.*

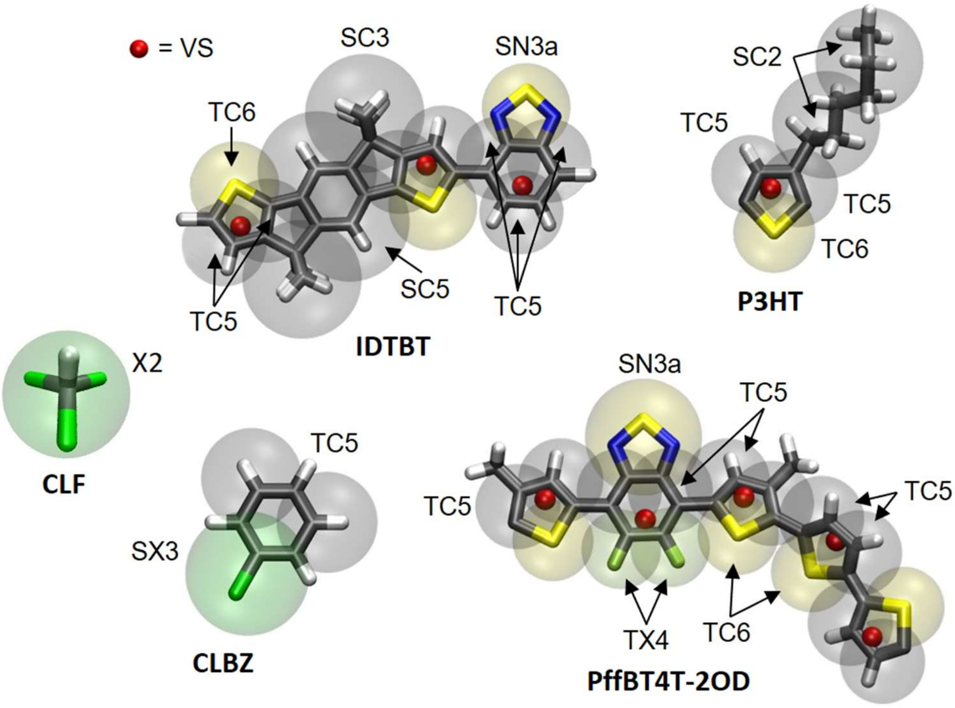

Figure S9: *Mapping between coarse-grained beads and atomistic structures for the molecular systems simulated by molecular dynamics in this work. Martini bead types are labelled. Longer sidechains are replaced with methyl groups for clarity. Virtual sites (VS) are marked with opaque red beads. Image adapted, with permission, from* [2].

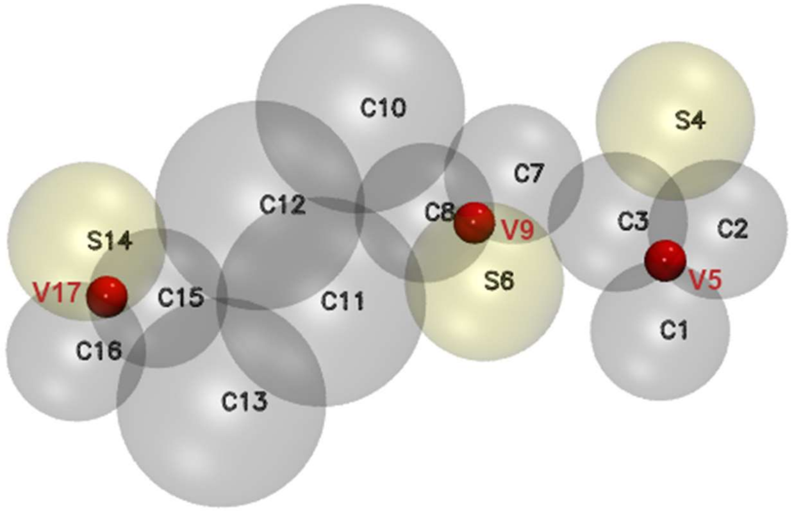

Figure S10*: Coarse-grained structure of IDTBT monomer. Beads have been labelled in black. Virtual sites are marked by opaque red beads and have been labelled in red.*

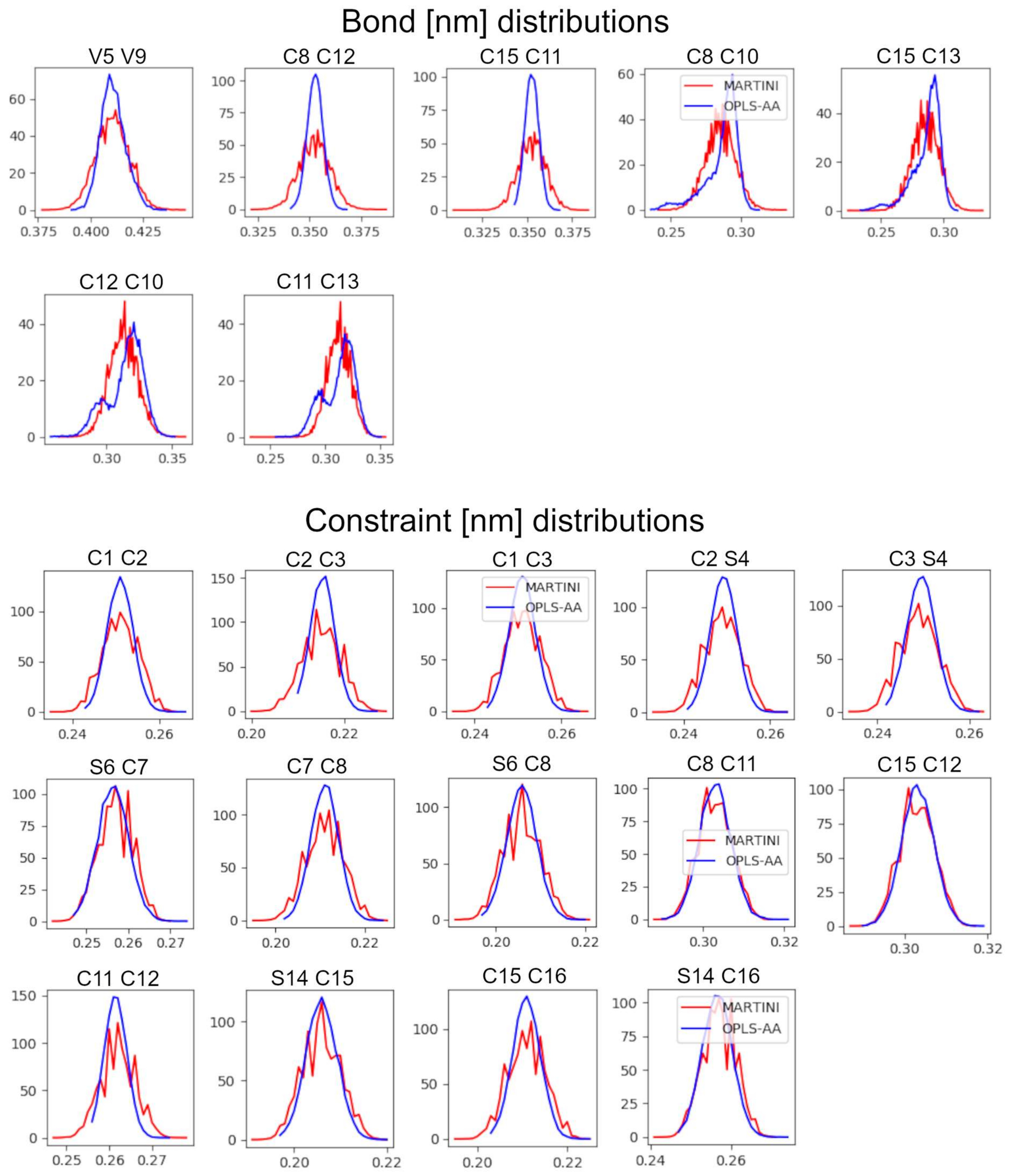

Figure S11: *Distributions of bond and constraint lengths, compared between CG (red) and atomistic (blue) forcefields. Labelled Martini and OPLS-AA, as these are the packages from which our forcefield were adapted. Data are from snapshots of a 100 ns simulation of C16-IDTBT 12mer chain in chloroform. Beads involved in each parameter are shown above distributions.*

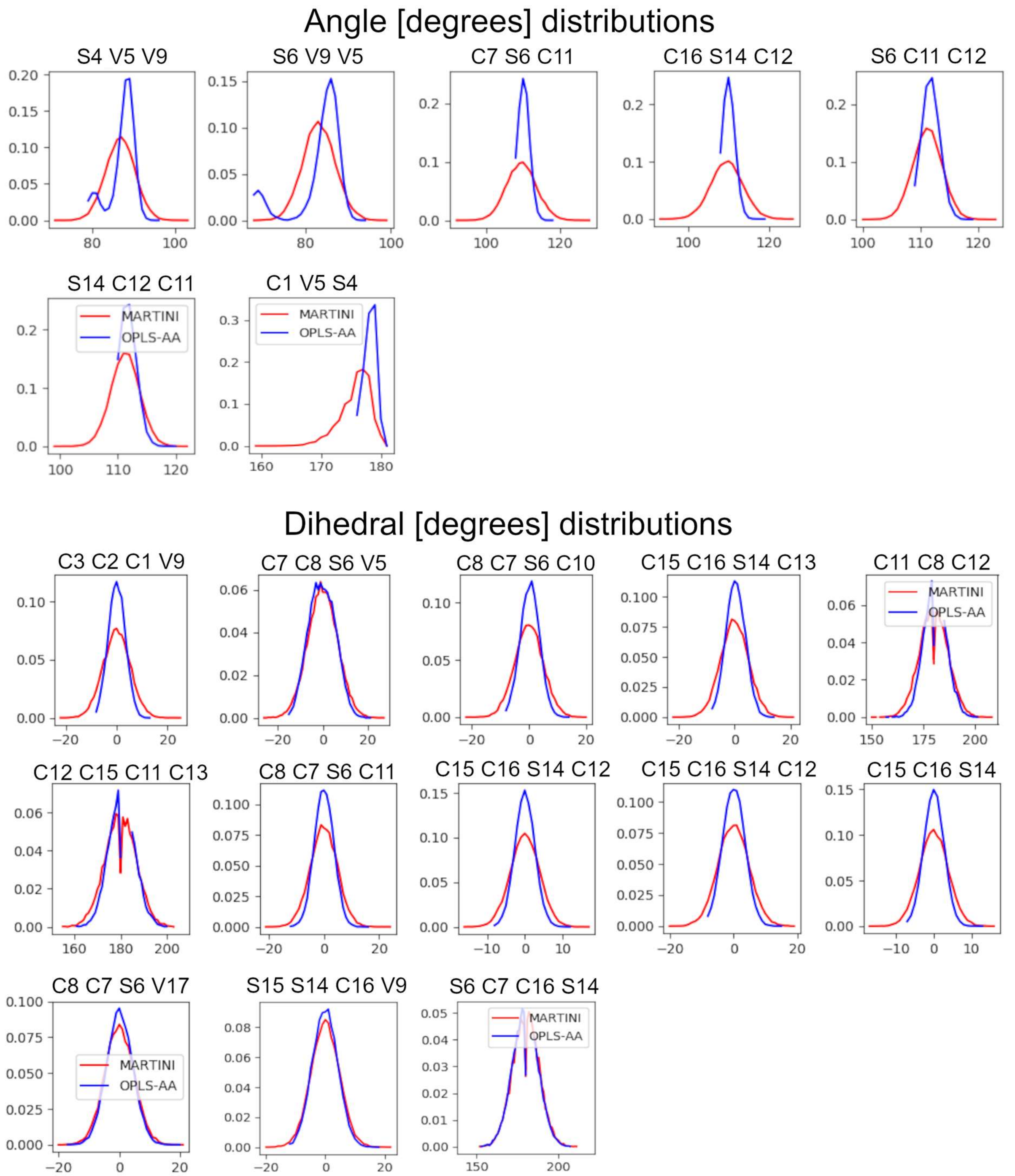

Figure S12: *Distributions of angles and improper dihedrals, compared between CG (blue) and atomistic (red) forcefields. Labelled Martini and OPLS-AA, as these are the packages from which our forcefield were adapted. Data are from snapshots of a 100 ns simulation of C16-IDTBT 12mer chain in chloroform. All dihedrals shown are improper dihedrals, put in place to hold the fused ring structures flat. The CG IDT-BT dihedral was parameterised independently from the atomistic forcefield, and is shown in* ***Figure S13***. *Beads involved in each parameter are shown above distributions.*

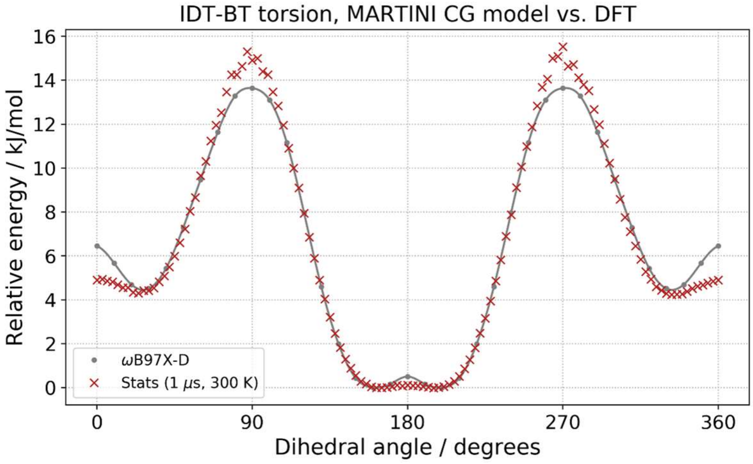

Figure S13: *Parameterisation of the IDT-BT dihedral angle for the CG forcefield, by comparison with torsional potential calculated by DFT. Red crosses are from average of all torsions during a 1 μs run of a 12mer in chloroform. DFT calculations were performed in vacuum, so small deviations are expected.*

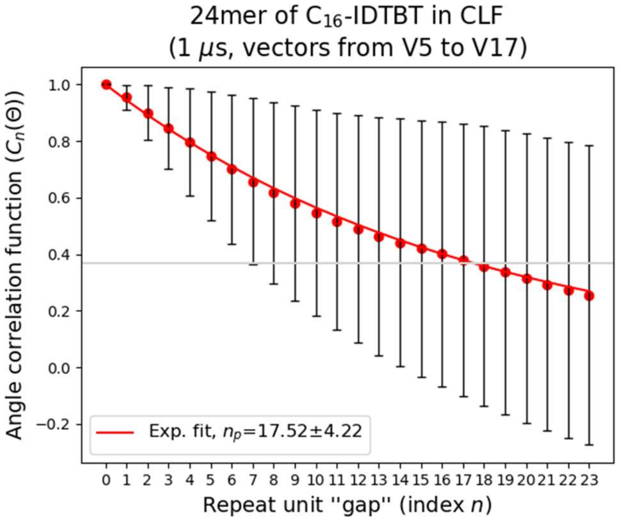

Figure S14: *Calculation of persistence length,* P*, from a 1 μs CG simulation of a C16-IDTBT 24mer chain immersed in chloroform. The angles* Θ_{ij} *between tangent vectors* $\hat{\boldsymbol{v}}_i$ *to an oligomer can be used to extrapolate a value for* P*. The plot shows* P *in units of monomers. It is equivalent to* P = 28.0 ± 6.7 nm.

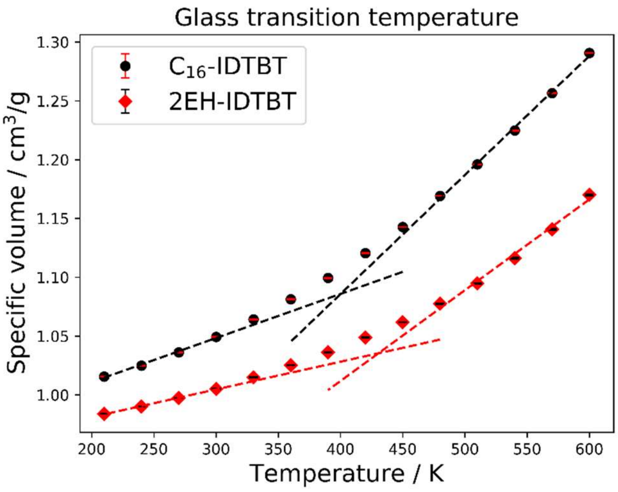

Figure S15*: Calculation of glass transition temperature for an atomistic C16-IDTBT 12mer thin film model and equivalent model of the polymer with ethyl-hexyl side chains (2EH-IDTBT).*

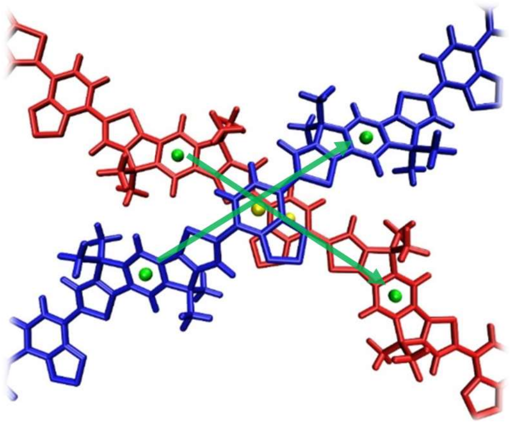

Figure S16: *Calculation of crossing angle. The diagram illustrates the origin of vectors (green arrows) used to evaluate crossing angle between two polymer backbones, in modelled systems.*

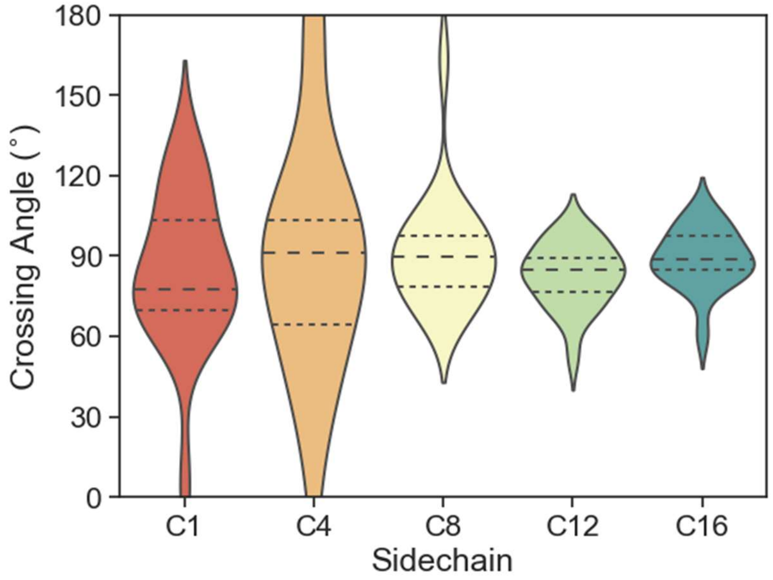

Figure S17: *Distribution of crossing angles for different sidechain lengths. 20 repeat simulations of two 12mer IDTBT chains deposited on substrate were performed for each sidechain length. Dashed lines represent the median and interquartile range.*

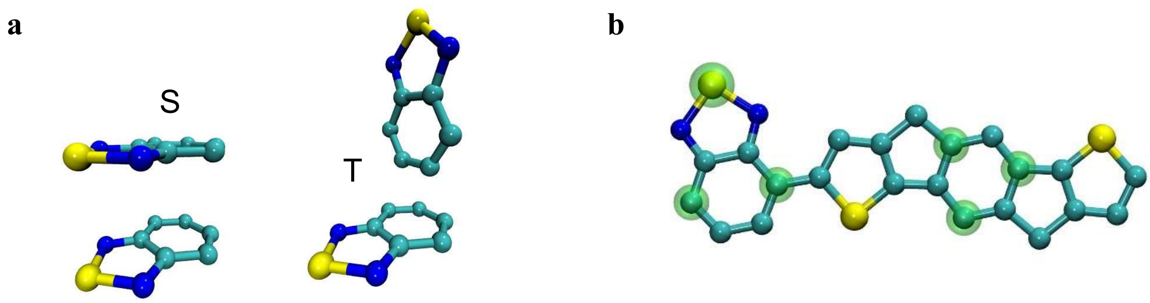

Figure S18: *Relative rotation of backbones in contact. (a) Diagrams illustrating S and T orientations of BT units. (b) An IDTBT monomer, with sidechains and hydrogen atoms hidden. To determine the degree of relative rotation, the orientation of the BT and IDT units are defined using the vector orthogonal to the plane of these units, where the plane is chosen to intersect through the three atoms highlighted in green.*

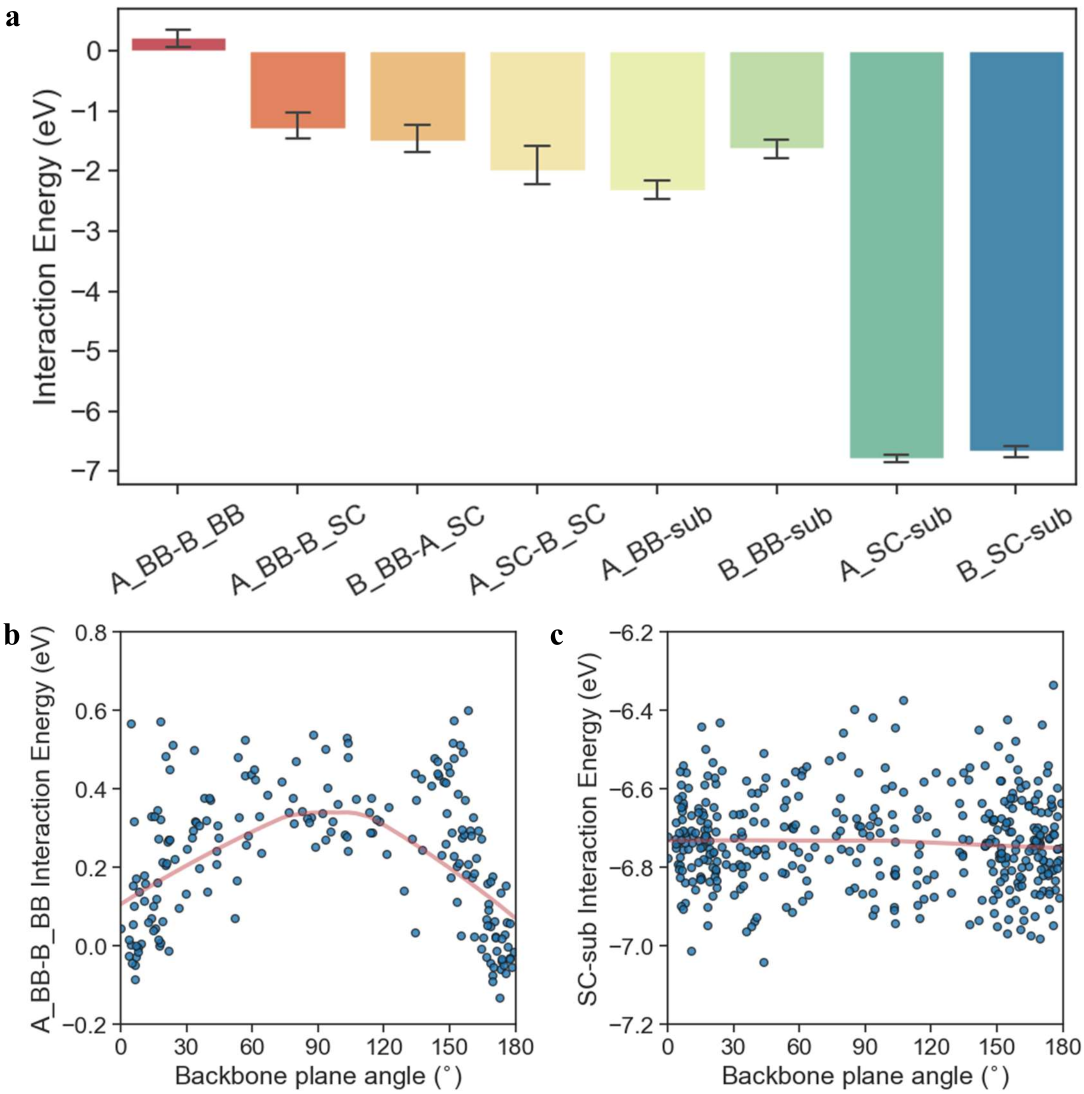

Figure S19: *Interaction energy trends for pairs of C16-IDTBT chains on substrate. (a) contributions from different components of the system. A and B refer to the two polymer chains, BB to backbone atoms, SC to sidechain atoms, and sub to the substrate. Error bars indicate the interquartile range. (b) and (c) show the relationship of interaction energy between different components and the angle between the planes of the backbone units in contact (i.e. their relative rotation). Interaction energies involving the substrate are divided by 12, the number of monomers in each chain. The red lines are LOWESS functions which serve as a guide to the eye. All interaction energies are calculated using empirical forcefields (the same as used in molecular dynamics simulations).*

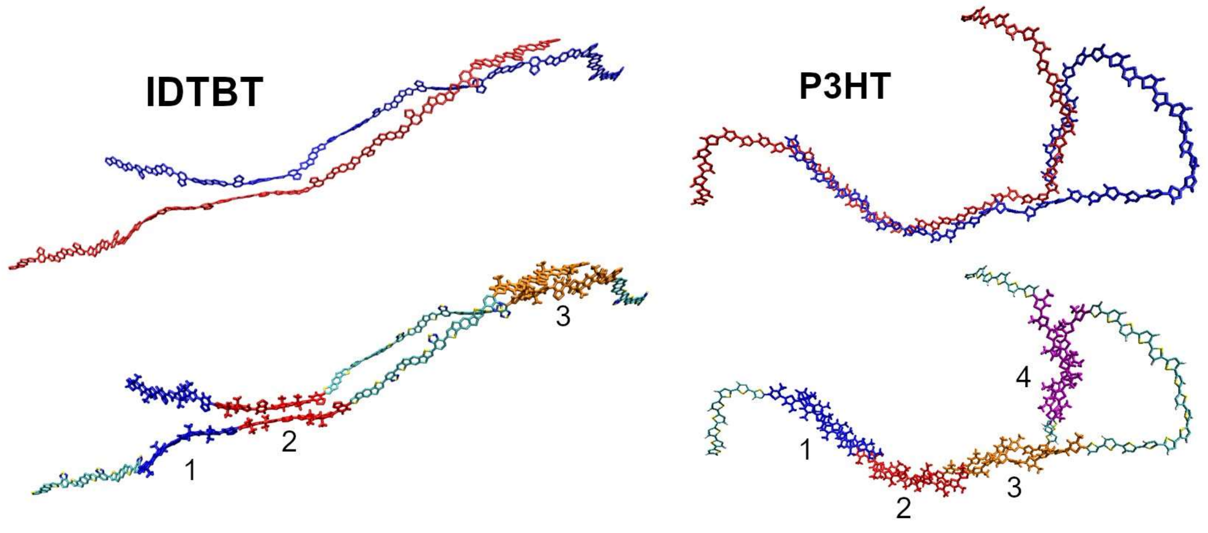

Figure S20: *Identification of interchain contacts. Diagram shows how multiple contact points are located between pairs of chains. In the top two images, the two chains are coloured red and blue respectively. In the bottom two images, the unused sections of the two chains are coloured according to their atom types (e.g. carbon atoms are cyan). The solid-coloured segments are those associated with contact structures. The contact structures have been numbered.*

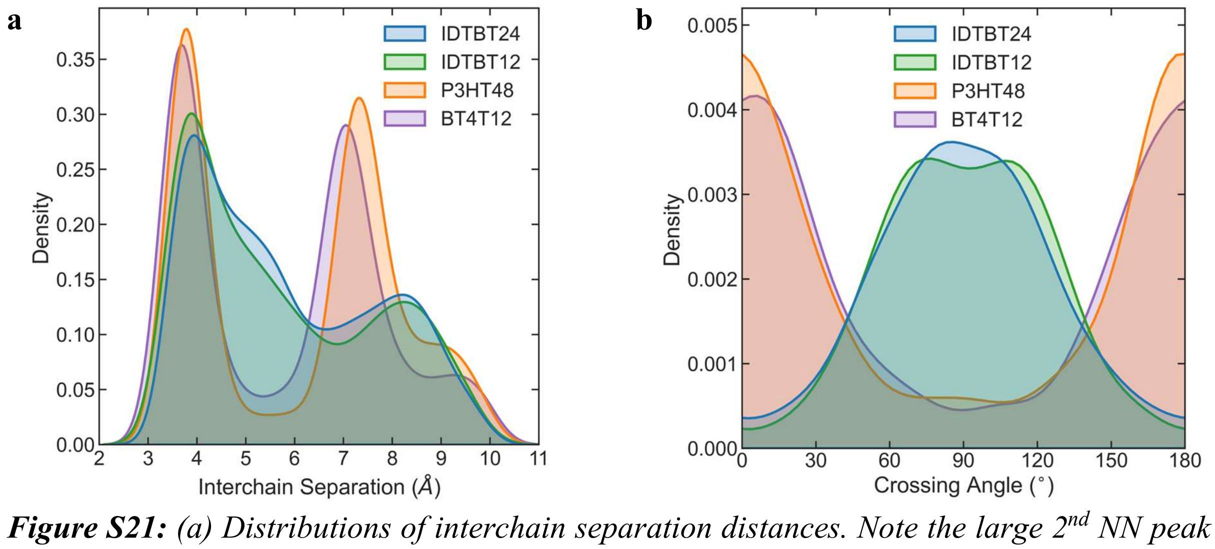

Figure S21: *(a) Distributions of interchain separation distances. Note the large 2nd NN peak for P3HT and PffBT4T-1OD, due to the presence of more repeated packing structures in these more ordered polymer systems. (b) Distribution of crossing angles at close contacts (ring-to-ring distance < 6 Å) in backmapped systems of C16-IDTBT 24mers, C16-IDTBT 12mers, P3HT 48mers and PffBT4T-1OD 12mers.*

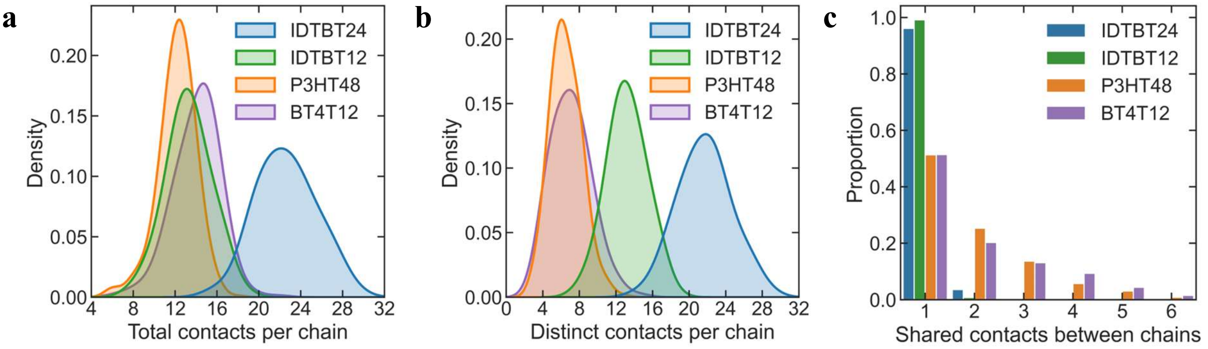

Figure S22: *(a) Distributions of the total number of close contacts made by each chain to any other chain. If multiple contacts are shared between a pair of chains, they are all counted. (b) Distributions of the number contacts to distinct chains made by each chain. If multiple contacts are shared between a pair of chains only one count is recorded. (c) The number of contacts shared between paired chains (those with at least one contact between them). Contact statistics are gathered for backmapped systems of C16-IDTBT 24mers, C16-IDTBT 12mers, P3HT 48mers and PffBT4T-1OD 12mers. For all of the above results, only close contacts (separation distance between centre-of-geometry of rings < 6 Å) were counted since these are the most relevant for transport.*

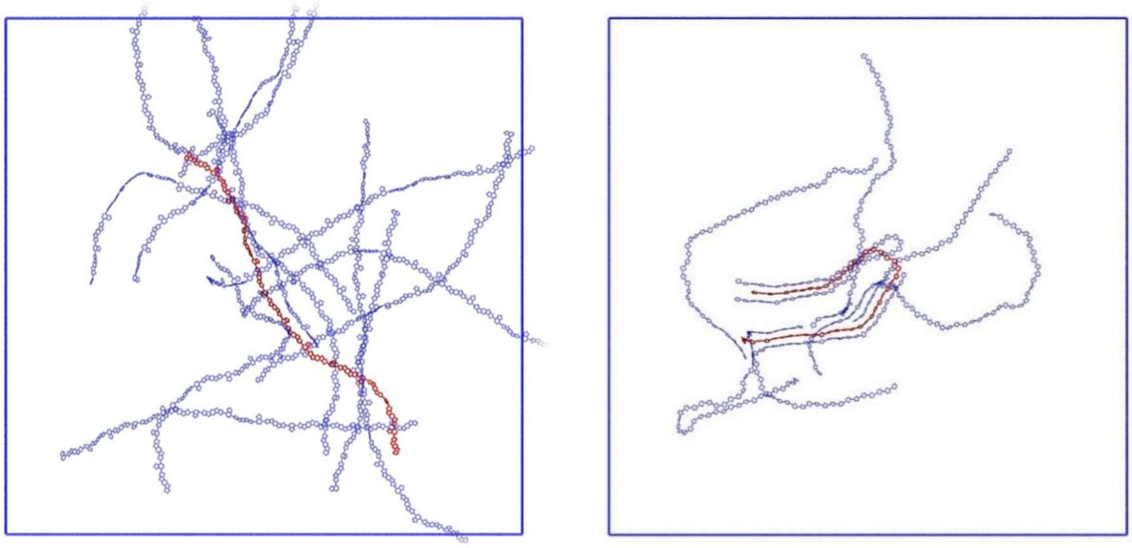

Figure S23: *Visual depiction of interconnectivity of films. Images show backmapped films, where most chains have been hidden. One chain is shown (red) plus all neighbouring chains (semi-transparent blue) in close contact with that primary chain. Left image shows C16-IDTBT 12mer system, right image shows P3HT 48mers. Simulation bounding boxes are also shown as blue squares. Box sizes are 250×250×94 nm (C16-IDTBT) and 250×250×78 nm (P3HT).*

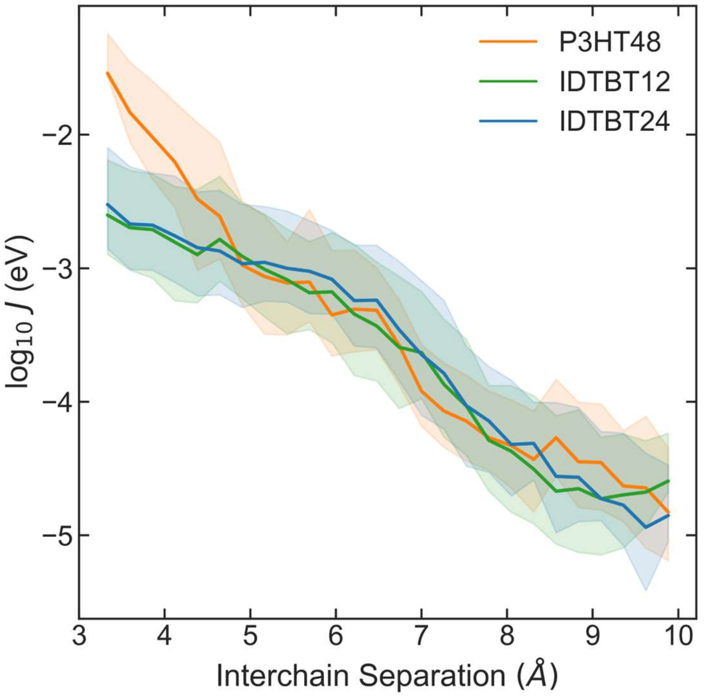

Figure S24: *Distance dependence of coupling strength at contact locations. Shaded area represents interquartile range (i.e. middle 50% of values lie within shaded area). Interchain separation corresponds to the closest distance between the centres-of-geometry of 5 or 6 member rings belonging to chain segments in contact. Contact geometries are taken from backmapped systems of C16-IDTBT 24mers, C16-IDTBT 12mers and P3HT 48mers.*

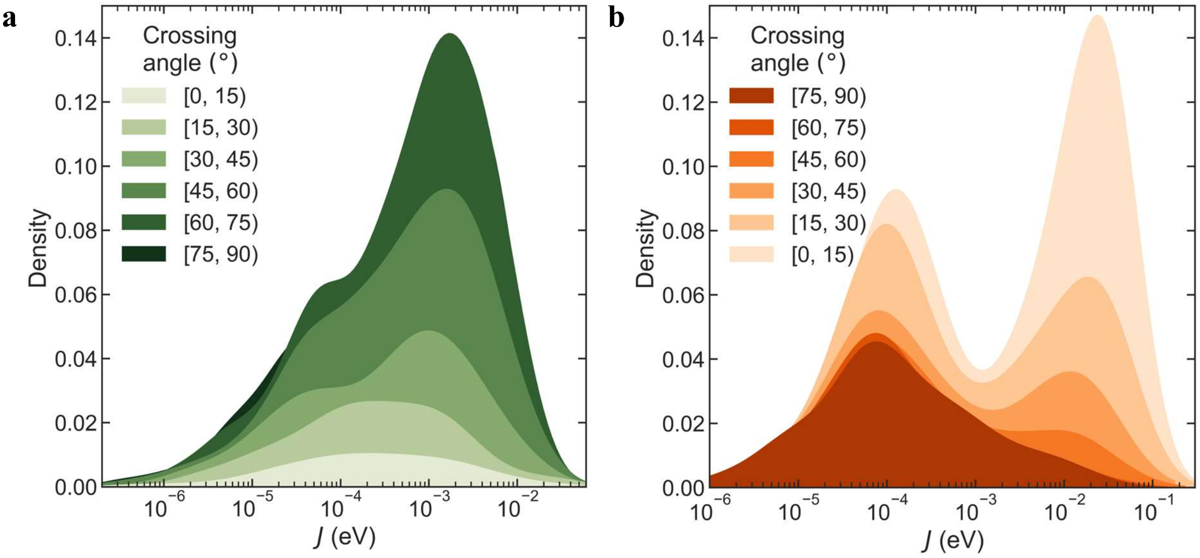

Figure S25: *Distribution of* J *for contacts extracted from the thin film models of (a) C16-IDTBT 12mers, and (b) P3HT 48mers. Distributions are binned according to the crossing angle between backbones. Darker shaded distributions represent more perpendicular crossing angles. Note that the distributions corresponding to larger crossing angles are drawn at the back for (a) but in front for (b). The highest coupling values tend to be associated with more perpendicular crossing angles in C16-IDTBT, and more parallel crossing angles in P3HT.*

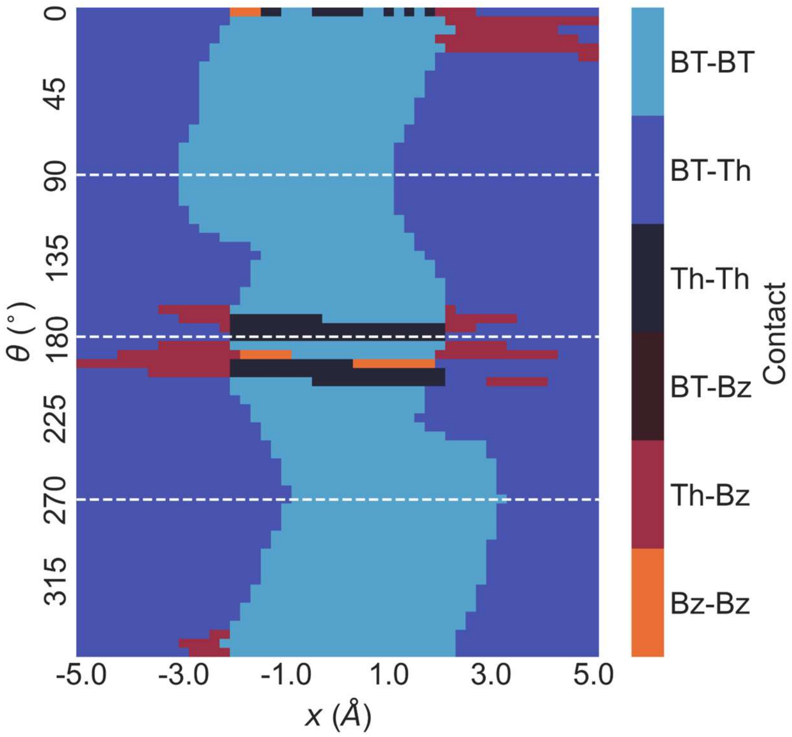

Figure S26: *Map of moieties in closest contact between a pair of IDTBT 2mers shifted and rotated, at fixed z-separation of 4 Å.*

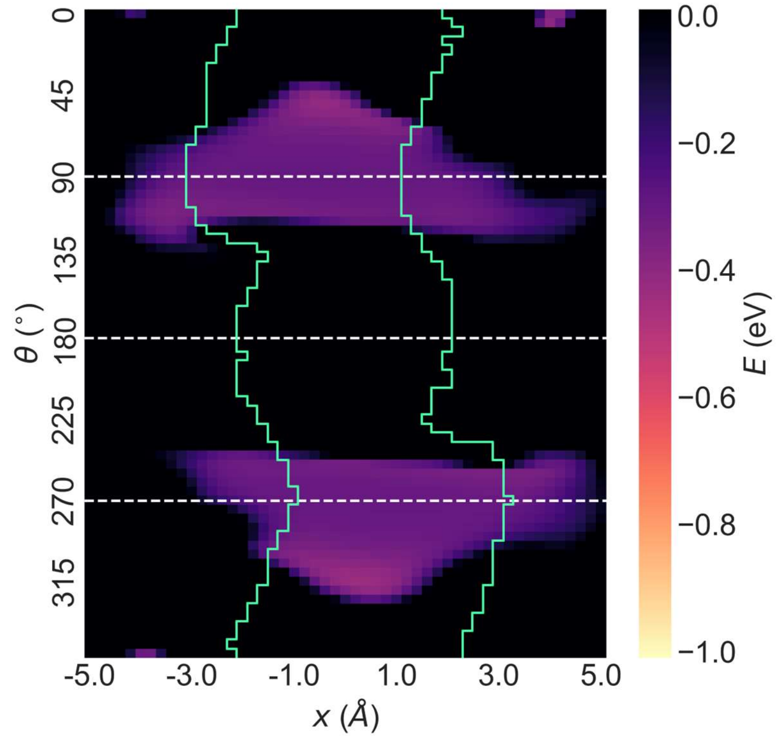

Figure S27: *Heatmap displaying the interaction energy for different arrangements of a pair of IDTBT 2mers. The green lines mark the boundary between BT-BT contact (inside the green lines) and BT-Th contact (outside the green lines). Note that small areas where other moieties are in closest contact have been neglected. Energies are calculated using empirical forcefields (same as molecular dynamics simulations).*

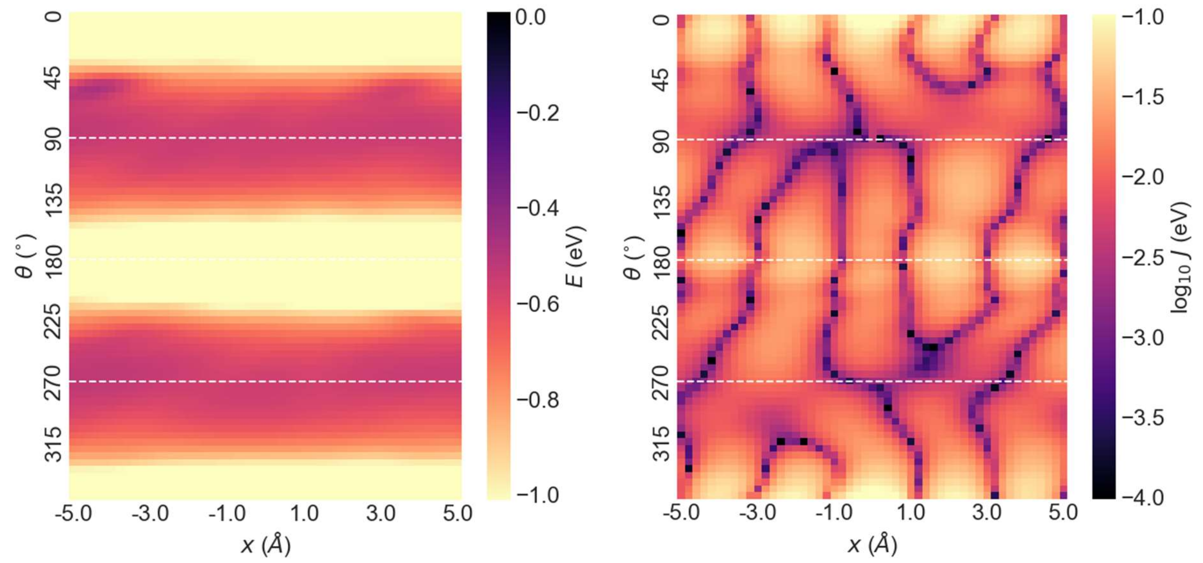

Figure S28: *Heatmaps describing the relationship between arrangements of P3HT chain segments and interaction energy (left) or HOMO-HOMO coupling (right). Segments are placed at fixed z-separation of 4 Å. Calculations are performed using DFT, at the cam-b3lyp/cc-pVDZ level of theory, with Grimme's D3 dispersion correction.*

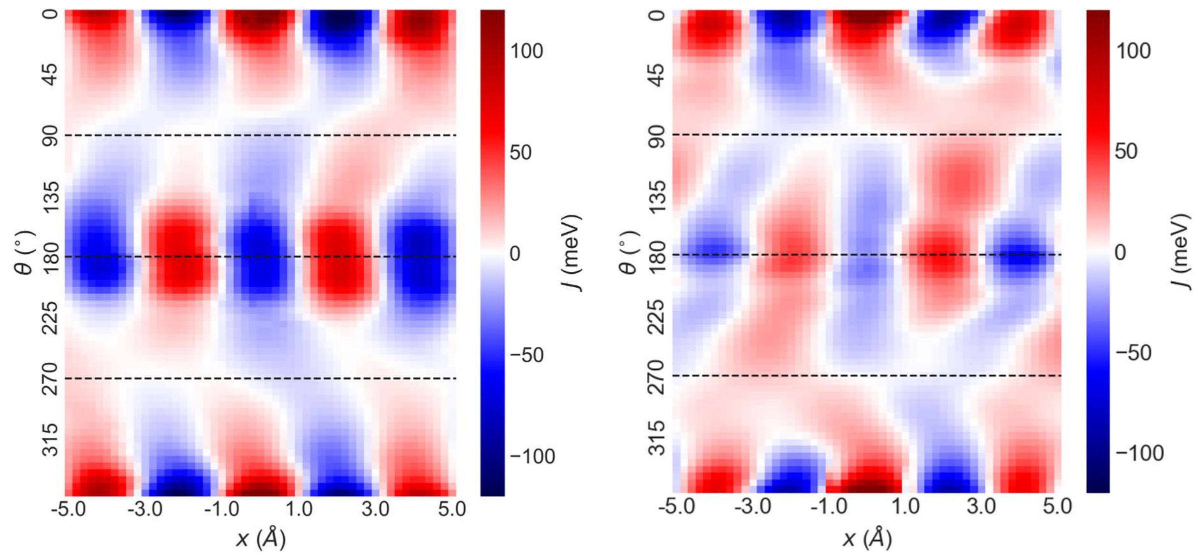

Figure S29: *Heatmaps describing the relationship between arrangements of IDTBT (left) or P3HT (right) chain segments and HOMO-HOMO coupling, shown on a linear scale. Calculations are performed using DFT, at the cam-b3lyp/cc-pVDZ level of theory with Grimme's D3 dispersion correction.*

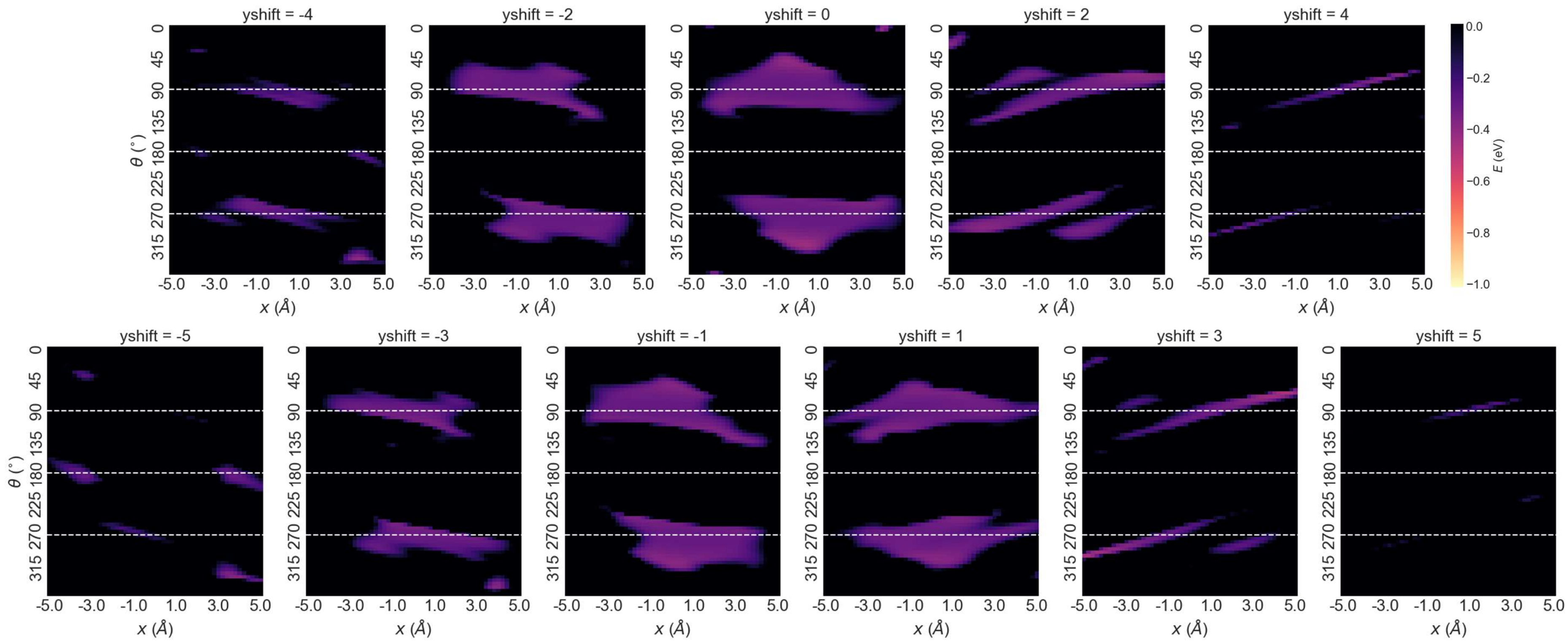

Figure S30: *Interaction energy heatmaps for IDTBT 2mer dimer. Each heatmap represents a different translation in the y-position of the shifted backbone segment. To reduce computational cost compared to DFT, to calculate the interaction energies shown here, we performed single point calculations using an empirical forcefield approach (using the same forcefield as in the atomistic MD simulations.)*

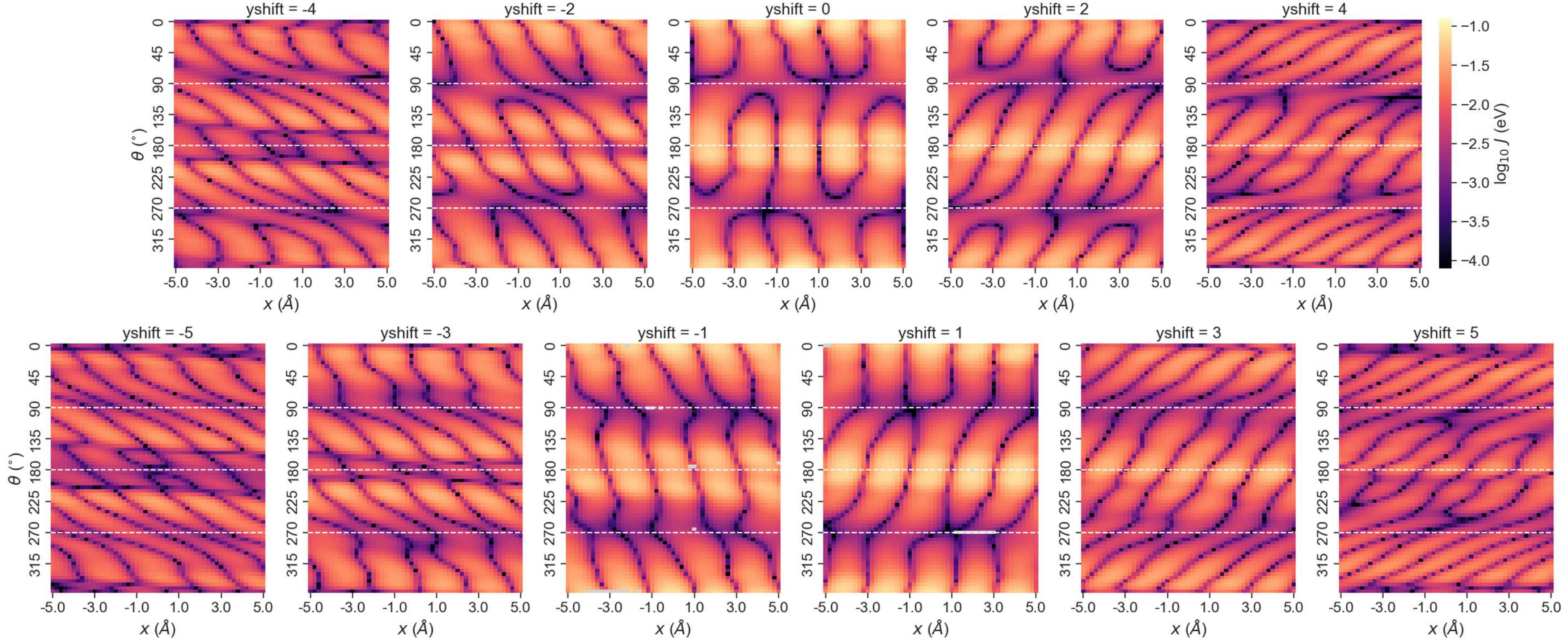

Figure S31: *Coupling heatmaps for IDTBT 2mer dimer. Each heatmap represents a different translation in the y-position of the shifted backbone segment. To reduce computational cost compared to DFT, for the coupling data shown here we adopted the molecular orbital overlap (MOO) method described in* [3]*. Making use of the ZINDO Hamiltonian, this method requires only a single self-consistent field calculation, performed on an isolated molecule, to estimate the electronic coupling between a pair of identical molecules.*

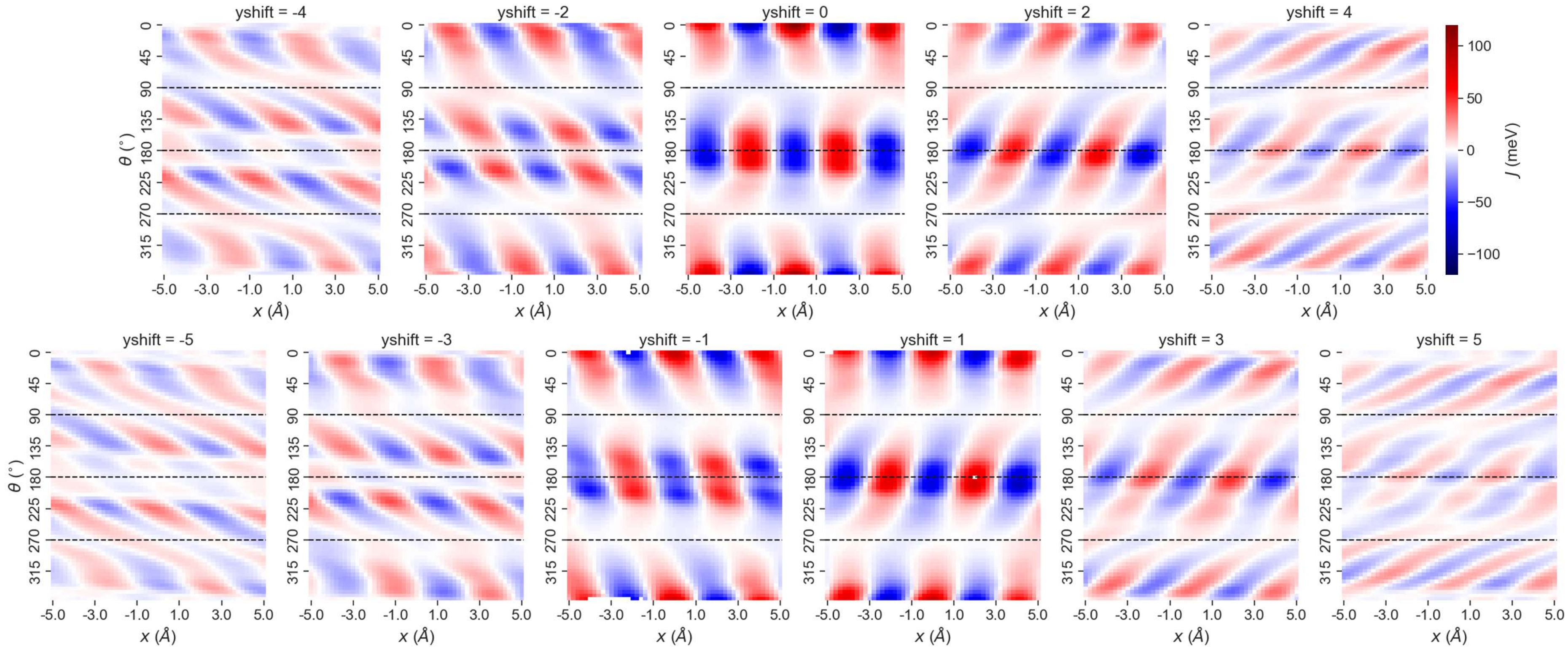

Figure S32: *Coupling heatmaps for IDTBT 2mer dimer, shown with linear scale. Each heatmap represents a different translation in the y-position of the shifted backbone segment. To reduce computational cost compared to DFT, for the coupling data shown here we adopted the molecular orbital overlap (MOO) method described in [3]. Making use of the ZINDO Hamiltonian, this method requires only a single self-consistent field calculation, performed on an isolated molecule, to estimate the electronic coupling between a pair of identical molecules.*

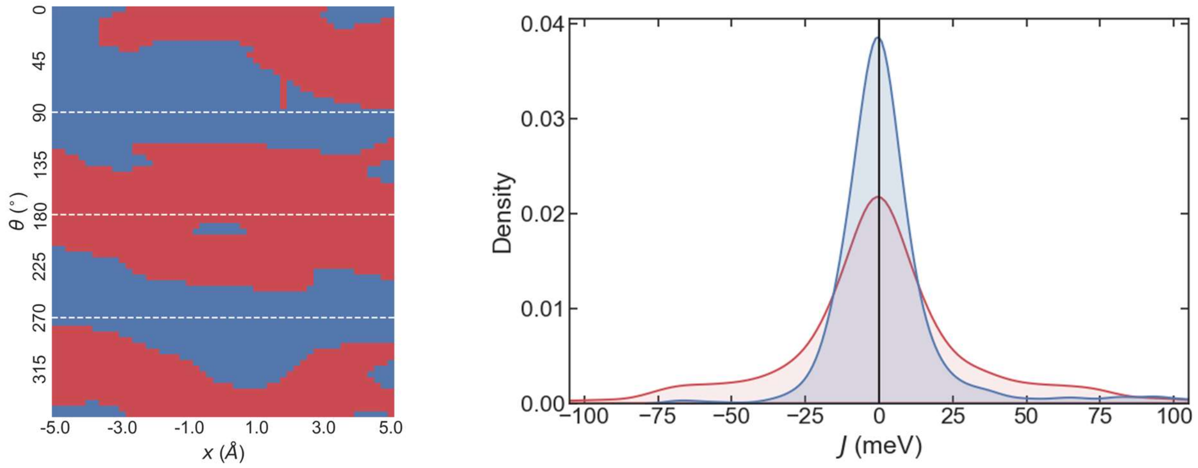

Figure S33: *Impact on coupling of restricting molecular arrangement of contacts. (Left) Map of enthalpically favoured (interaction energy < 0) contact geometries in blue, versus disallowed geometries in red. (Right) Distribution of coupling strength while restricting to only allowed geometries is shown in blue. Distribution of coupling strength across full range of tested geometries is shown in red. The distribution of coupling values narrows when we limit molecular arrangements to those that are enthalpically favoured.*

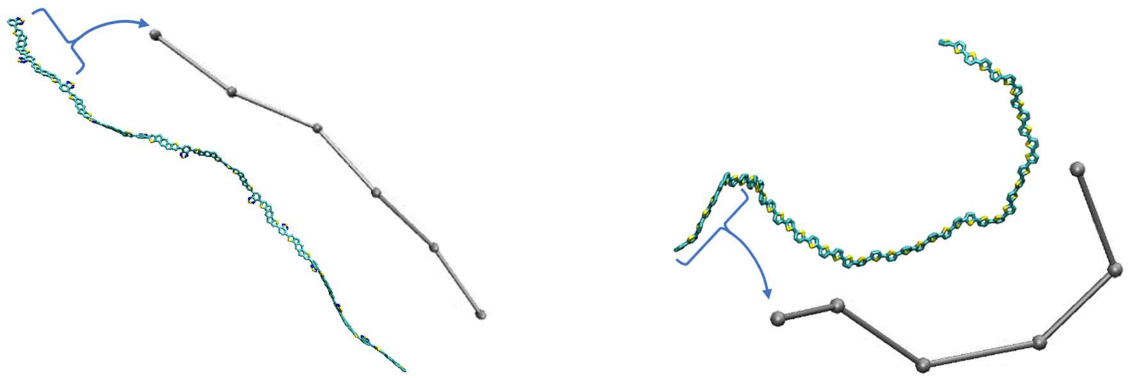

Figure S34: *Conversion from atomistic representation to site representation based on coordinates of π-system atoms. (Left) C16-IDTBT, each 2mer segment is converted into a site. (Right) P3HT, each 8mer segment is converted into a site. Note that the segments described here may not line up exactly with the segments chosen when extracting and calculating coupling between contacts (which may be shifted along the backbone, in monomer increments, to maximise the degree of contact). When using the coupling to weight edges in the transport network, the chain segments from which coupling was calculated are associated with their closest site (on the same chain).*

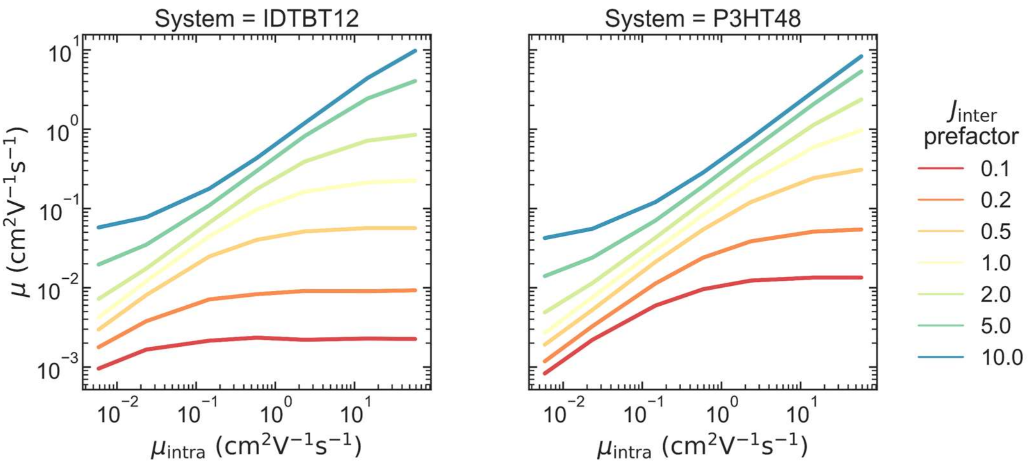

Figure S35: *Mobilities predicted by kinetic Monte Carlo simulations after multiplying all (calculated) interchain coupling strengths by a constant prefactor. (Left) C16-IDTBT 12mer system. (Right) P3HT 48mer system.*

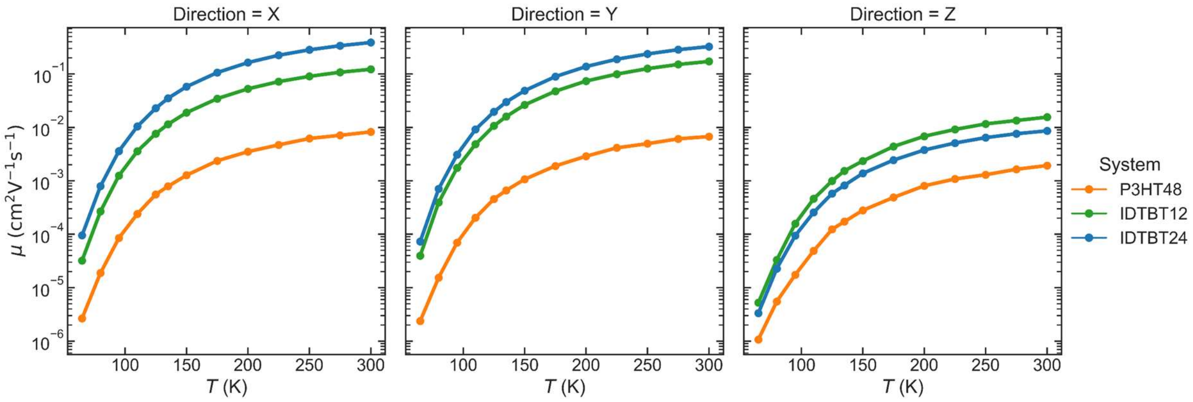

Figure S36: *Temperature dependence of mobility as predicted by kinetic Monte Carlo simulations. Different lines represent C16-IDTBT 12mer, C16-IDTBT 24mer, and P3HT 48mer systems.* μ_{intra} *values for C16-IDTBT and P3HT are set to the experimentally informed values discussed in Section 2.4 of the main paper (3* $cm^2V^{-1}s^{-1}$ *and 0.02* $cm^2V^{-1}s^{-1}$ *respectively).*

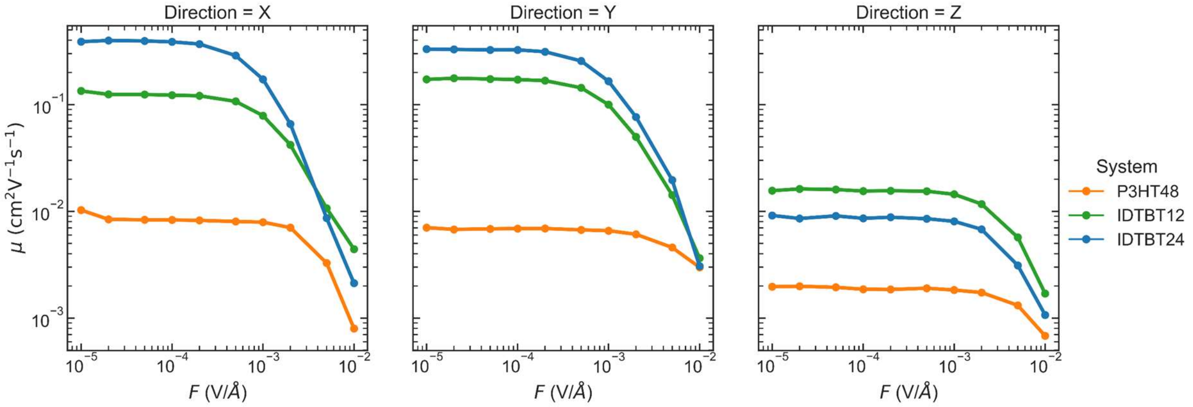

Figure S37: *Field strength dependence of mobility as predicted by kinetic Monte Carlo simulations. Lines represent C16-IDTBT 12mer, C16-IDTBT 24mer, and P3HT 48mer systems.* μ_{intra} *values for C16-IDTBT and P3HT are set to the experimentally informed values discussed in Section 2.4 of the main paper (3* $cm^2V^{-1}s^{-1}$ *and 0.02* $cm^2V^{-1}s^{-1}$ *respectively).*

Supporting Tables

Table S1: *Polystyrene equivalent molar masses and model fitting results for samples measured by SANS. Number average <Mn> and mass average <Mw> molar masses are given in kg/mol. Contour length (*L*), Kuhn length (*b*), radius (*s*), and persistence length (*P*) limits are given in Å. Uncertainties are based on an analysis of the parameter distributions covering 68% of the distribution.*

				**Flexible cylinder**			**Rigid cylinder**		
**Sample**	**<M_n>**	**<M_w>**	**Đ**	***L***	***b***	***s***	***L***	***s***	***P* limit**
I	32.5	44.7	1.38	136.9 ± 8.2	134 ± 18.6	18.7 ± 0.7	134.2 ± 8.7	19.3 ± 0.6	> 68.4
II	64.5	195	3.02	1080 ± 290	510 ± 270	13.1 ± 4.5	/	/	< 390

Table S2: *Summary of details of coarse-grained molecular dynamics simulations.*

**Polymer**	**Chain length (no. monomers)**	**No. chains**	**Solvent**	**Initial box dimensions, x × y × z (nm^3)**	**Final box dimensions, x × y × z (nm^3)**	**Final density (g cm^{-3})**	**Repeats**
C16-IDTBT	24	300	CLF	45×45×115.2	45×45×7.2	1.06	1
C16-IDTBT	12	250	CLF	25×25×155.5	25×25×9.4	1.09	3
P3HT	48	420	CLBZ	25×25×80.8	25×25×7.4	1.14	3
PffBT4T-2OD	12	280	CLBZ	25×25×142.4	25×25×7.7	1.23	3

Supporting Notes

Section S1: Small Angle Neutron Scattering

Small angle neutron scattering (SANS) measurements were performed on two samples of C16-IDTBT polymer chains immersed in solution, with different molar mass distributions, which we label I and II. Sample I was measured in deuterated chloroform (CLF) while sample II was measured in perdeuterated chlorobenzene (CLBZ). All measurements were performed at 25 °C with active temperature control. All samples were measured at the NIST Center for Neutron Research (NCNR) in Gaithersburg, Maryland. Solutions of Sample I (with concentrations of ~4 mg/mL, 2 mg/mL, and 1 mg/mL) were measured on the NGB 10 m SANS instrument and solutions of Sample II (with concentrations of ~1 mg/mL, ~0.6 mg/mL, and ~0.3 mg/mL) were measured on the NG-7 30 m SANS instrument[4]. Data were reduced and converted to absolute intensity dΣ/dΩ(q)=I(q) using the NCNR's Igor[†] Macros.[5], where q is the modulus of the scattering vector, $q = \frac{4\pi}{\lambda}\sin\left(\frac{\theta}{2}\right)$ (with λ the wavelength of the neutrons and θ the angle between the incident and scattered beam).

Attempts were made to fit the scattering data using Bumps[6] and sasmodels[7] for each sample to flexible cylinder and rigid cylinder models. Sample I was simultaneously fit to all 3 concentrations and could be fit with both models. Fits to the flexible cylinder model yielded Kuhn lengths, b, equal to contour lengths, L. L was statistically equivalent between the two models for Sample I. Using the contour length L=13.4 nm and repeat unit length 1.6 nm indicates that the correct <Mw> is ≈11 kg mol^{-1} and that GPC overestimated <Mw> by a factor of 4.1. It should be noted that typical values for the overestimate of polystyrene equivalent molar mass for semiconducting polymers range from 1.3 to ~3[8,9], further reinforcing the high stiffness of C16-IDTBT. These results indicate that, for true molar masses less than 11 kg mol^{-1}, C16-IDTBT polymer chains behave like rigid rods. Thus, halving the values of L determined from these models gives us a lower limit for the persistence length, P.

The higher molar mass sample, II, could not be fitted to a rigid cylinder model, indicating that P must be shorter than the chain length. Due to an upturn at low q, Sample II was fit to a linear combination of the flexible cylinder model and the Debye-Anderson-Brumberger model[10]. The fit was constrained via the following relationship[11]:

$$\frac{d\Sigma}{d\Omega}(q=0) = \Delta\rho_{SLD}^2 c\left(\frac{M_w}{N_A\rho_p^2}\right), \quad (1)$$

where $\Delta\rho_{SLD}^2$ is the scattering length density difference, M_w is the mass average molar mass, N_A is Avogadro's number, and ρ_p is the polymer density. For a flexible cylinder model, at q=0, using sasmodels, the intensity is

[†] Certain commercial equipment, instruments, or materials are identified in this paper in order to specify the experimental procedure adequately. Such identification is not intended to imply recommendation or endorsement by the National Institute of Standards and Technology, nor is it intended to imply that the materials or equipment identified are necessarily the best available for the purpose.

$$\frac{d\Sigma}{d\Omega}(q=0) = G\Delta\rho_{SLD}^2(\pi L R^2), \quad (2)$$

where G is a "scale" parameter, L is the contour length of the cylinder and R is the radius. (Note that in SasView and sasmodels L and R are input in Å and the ρ_{SLD}'s are in 10^{-6} Å^{-2}, so there is an additional factor of 10^{-4} in eq. 2 necessary to yield dΣ/dΩ in the standard units of cm^{-1}.) To account for sample polydispersity, a lognormal distribution with logarithmic spacing was used in sasmodels. The parameters of the molar mass distribution are related to the sasmodels polydispersity PD=σ via

$$M_w = \bar{M}\exp\left(\frac{\sigma^2}{2}\right), \quad (3)$$

where $\bar{M}$ is the median of the distribution[12]. By using the lognormal distribution function in sasmodels, L corresponds to the median value and therefore we can equate eq. 1 and 2 and substitute the following

$$M_w = \frac{\exp\left(\frac{\sigma^2}{2}\right) m_p L}{l_u}, \quad (4)$$

where l_u is the effective length of the monomer with molar mass m_p, and

$$\Delta\rho_{SLD} = \rho_{SLD,p} - \rho_{SLD,s} = \frac{b_p\rho_p}{m_p} - \frac{b_s\rho_s}{m_s}, \quad (1)$$

where b_p and b_s are the calculable scattering lengths of the polymer and solvent, respectively, m_s is the molar mass of the solvent, and ρ_s is the density of the solvent (assumed to be the same as the bulk). Using a density of 1.07 g cm^{-3} calculated from the crystal structure of C16-IDTBT[13] and a density of 1.157 g cm^{-3} for d5-chlorobenzene, the NCNR's neutron activation and scattering calculator[14] enabled determination of $\rho_{SLD,p} = 0.412 \times 10^{-6}$ Å^{-2} and $\rho_{SLD,s} = 4.909 \times 10^{-6}$ Å^{-2}. Combining the above equations yields

$$G = \frac{\exp\left(\frac{\sigma^2}{2}\right) c b_p^2}{\pi N_A \rho_{SLD,p}^2 m_p R^2 l_u}. \quad (6)$$

Uncertainties from the fits were estimated via the DREAM optimizer that combines a Markov Chain Monte Carlo sampler with a differential evolution step generator in Bumps (because of uncertainties in the true molar mass distribution, σ was allowed to float. For Sample II, σ_{fit}=0.79±0.19.) Based on the uncertainties, and assuming a single standard deviation in the fit uncertainties, an upper bound of 39 nm for the persistence length of Sample II was determined. It should be noted that solvent quality will affect the measured persistence length, so there is an additional uncertainty introduced by the different solvents. (Note that for Sample II, for the lower concentrations chosen so that the sample was in the dilute regime, it was necessary to change to d5-chlorobenzene for added contrast to increase the scattering intensity.) The results of the fits are summarized in **Table S1**.

Section S2: Pulse Radiolysis Time-Resolved Microwave Conductivity

Mobility is determined by $\mu = \sigma/Ne$, where σ is the measured conductivity, N is the number density of charges, and e is the charge of an electron. Pulse radiolysis time-resolved microwave conductivity (PR-TRMC) measurements determine the conductivity per unit dose of ionizing radiation and PR with transient absorption determines the concentration of charges, also per unit dose. The mobility can therefore be calculated from the ratio of curves in **Figure S1** and **Figure S2**, divided by the charge on an electron. The right-hand axis scaling of **Figure S2** is used to give the standard mobility units, and an average over data from 5 – 10 microseconds was used. Due to the random orientation of polymer chains with respect to the microwave field, the isotropic mobility that we measure directly is multiplied by 3 to give a value of 1.0 ± 0.1 $cm^2V^{-1}s^{-1}$ for the (real) mobility of holes along the chain. This value was independent of the electron scavenger used. This is a lower limit due to effects from finite chain length, chain curvature and defects. The imaginary mobility, which relates to the displacement (rather than velocity) of charge, occurring in phase with the microwave field, was also determined to be 1.0 ± 0.1 $cm^2V^{-1}s^{-1}$. An imaginary component of mobility, which means an increased polarizability, results in an increased dielectric constant of the solution which shifts the resonance frequency of the cavity.

Section S3: Scanning Tunnelling Microscopy

3.1. Imaging and Fitting Procedure

Individual polymer chains are seen as strands marked by a succession of bright dots in scanning tunnelling microscopy (STM) images, as shown in **Figure S4**. The sidechains are darker and are highly interdigitating in the areas between the backbones.

In high molecular coverage areas, the polymers tend to form a rather compact assembly through the interdigitation of their side chains. This assembly is characterized by small (10-20 nm wide) ordered areas where the backbones are locally straight, equally spaced, and parallel to each other, as can be seen in **Figure S5** (a). Neighbouring aggregates are typically connected via longer polymers, that bend to accommodate the change in orientation of the domains. The overall effect is that randomly oriented nanoaggregates are bridged by longer polymers.

Even at lower coverages, the polymers can be seen to interdigitate with neighbouring molecules, forming small compact islands, with a few polymers occasionally bridging between islands, as shown in **Figure S5** (b).

The internal structures of the polymers can be distinguished in high-resolution images, as shown in **Figure S6** (a). The backbones appear as sequences of bright features, caused by the presence of sp^3-hybridised carbon atoms on both sides of the IDT groups, at periodic intervals of (1.6 ± 0.1) nm. Protruding perpendicularly from the backbones, alkyl side chains interact with the side chains of neighbouring molecules, forming densely packed structures.

This information was used to fit the STM images with geometry-optimized molecular models, as shown in **Figure S6** (b). For each repeat unit, the two pairs of side chains were first identified and used to precisely locate the two sp^3-hybridised carbon atoms. From these, the position and orientation of the IDT units was determined. For completeness, molecular models for the BT

units were also inserted in the space remaining between IDT subunits. However, their orientation was assigned randomly as it cannot be determined from the STM images. Further details on the imaging of IDTBT by STM have been discussed in previous work [15].

Using the same fitting procedure described above, we were able to determine the molecular-scale structure of crossing polymer backbones at points of interchain contact, as shown in **Figure 1** (c) in the main paper. Interestingly, this fitting procedure invariably leads to only one of the two crossing backbones being well represented, with the other seemingly shorter than expected (contracted along the backbone main axis). The two flanking IDT comonomers do not appear to leave enough space for the central BT unit at the point of contact. The reason for this apparent error is to be sought in the 3D nature of the crossing between the two chains. In the STM images, one of the two polymers always appears locally brighter in proximity to the crossing point and can thus be identified as the molecule crossing over the other one (blue in **Figure 1** (c)). It is always this higher-lying polymer that is poorly represented in the fitting procedure because its backbone is not completely parallel to the substrate (see **Figure S7**). In fact, what is measured in the STM images is not the actual structure of the backbone but its projection onto the surface plane. Thus, if the polymer is not coplanar with the substrate, its structure appears to be shorter. If the 3D nature of the crossing is accounted for in the fitting of the STM images, the agreement can be re-established quantitatively. The modified fitting model assumes that: i) The two crossing BT units are parallel to each other and separated by a distance d = 3.8 Å, corresponding to the π-π stacking distance typically measured for IDTBT in GIWAXS[16], and ii) besides the BT group, only the two IDT units flanking the crossing point are at a higher position with respect to the rest of the polymer, which is directly absorbed on the substrate. Based on these assumptions, geometry-optimized molecular models for the projection of the IDT units onto the surface plane were constructed, tilting them by an angle ϴ defined as

$$\Theta = \tan^{-1}\left(\frac{d}{l}\right), \quad (7)$$

with l = 11.8 Å being the length of an IDT unit as measured in Avogadro[17]. When the rescaled backbone is overlayed onto the STM images, all the units can be properly allocated, as shown in **Figure 1** (c).

3.2. Backbones at Crossing Points

In the high-resolution transmission electron microscopy (HRTEM) results reported by Cendra *et al.*[1], different domains of locally parallel oriented molecules partially overlap at relative angles that span a large range of values, with a preference for angles of 20° (followed by 90°). This only apparently contradicts the strict 90° relative orientation of chains in contact found in the STM images because it must be remembered that the lines in the reconstructed HRTEM image do not represent individual polymer backbones but the predominant local polymer orientation. As already discussed in the main paper, a first reason for this discrepancy is that adjacent domains observed in the HRTEM analysis may sometimes comprise the same groups of chains bending as they pass from one domain into another. Another reason for the HRTEM analysis reporting crossing angles different from ~90°, is that the line crossings in HRTEM describe the relative average orientation of the domains, and not the actual orientation of the individual polymer backbones at the crossing points. The higher resolution of the STM images reveals that, while the polymers are always perpendicular to each other at the crossing point, they often bend just before and after it, so that the average local orientation of the domains can be quite different (see **Figure S8**), producing a wide variety of relative angles in excellent agreement to what was reported by Cendra *et al.* [1].

Section S4: Forcefield Parameterization

4.1. Atomistic Forcefield

The atomistic C16-IDTBT forcefield was adapted from a previously published forcefield constructed for (5Z,5'Z)-5,5'-((7,7'-(4,4,9,9-tetraoctyl-4,9-dihydro-s-indaceno[1,2-b:5,6-b']dithiophene-2,7-diyl)bis(benzo[c][1,2,5]thiadiazole-7,4-diyl))bis(methanylylidene))bis(3-ethyl-2-thioxothiazolidin-4-one) (O-IDTBR). Parameterization details for the O-IDTBR forcefield may be found in the supporting information of [18]. The atom types, bond types, bond angles and torsional angles were reused, without modification, from the O-IDTBR forcefield. However, the partial charges were modified to capture the polymeric nature of C16-IDTBT. The process of assigning partial charges is described in [2] but is also given here for convenience.

A methyl-substituted trimer of IDTBT was optimized using density functional theory (DFT) at the same level of theory (B3LYP/6-311++G(d,p)) as the O-IDTBR acceptor. The bond lengths, angles, and dihedrals of the internal monomer were then used to construct the seven internal monomers in a 9mer, whereas the terminal monomers in the 9mer were kept as the corresponding terminal monomers in the trimer. This 9mer was then subject to a single point energy calculation at the same level of theory, after which the resulting ESP charges of the seven internal monomers were symmetrized and scaled to yield a net charge of zero. This resulted in a three-residue model with different charges on each terminal monomer and on the internal monomer, allowing one to build oligomers of arbitrary length. The final charges are listed in the topology files available at [19].

Validation of the O-IDTBR forcefield is described in [18]. To summarise here, validation came from two areas. Firstly, free energies of solvation values were calculated and compared across different solvents. The differences between solvents aligned qualitatively with experiments. Secondly, crystal lattice parameters, intermolecular separations and π-stacking orientations were shown to correspond well with values extracted from GIWAXS experiments.

To provide independent support for the resulting C16-IDTBT forcefield, we used it to calculate two different characteristics of the polymer to compare with experimental values. Firstly, the persistence length of chains in solution was calculated and compared with SANS experiments. The persistence length for the atomistic model was found to be 65 ± 22 nm. Although errors are large on both experimental and computational values, the values are significantly larger than both experimental and calculated values in the literature for more flexible polymers. The SANS experiments are introduced in Section 2.1 of the main paper, with further details provided in **Sections S1**. The calculation of persistence length from MD simulations is described further in **Section S5**. Secondly, we calculated the glass transition temperature, T_g, of C16-IDTBT thin film models. As shown in **Figure S15**, a value of ~400 K was found from our analysis. As expected, the value is lower than that for the same backbone functionalised with shorter, ethyl hexyl, side chains rather than hexadecyl. The value can be compared with an experimental value $T_g \approx 370$K obtained using dynamic mechanical analysis experiments, described in [20].

4.2. Coarse-Grained Forcefield

Although the Martini forcefield was not originally designed with fused ring systems in mind, previous studies have demonstrated its capability for modelling materials with these structures. An overview of the use of Martini in material science, including the modelling of conjugated

polymers and other organic semiconductors may be found at [21]. For an overview of its use for modelling small molecule organic semiconductors, see [22]. For an example of its use for modelling the bulk heterojunction morphology of an organic photovoltaic device, as well as a demonstration of backmapping to recover atomistic detail, see [23].

Figure S9 shows a visual depiction of the mapping between Martini beads and atomistic structures. Parameterization of the forcefields for solvent molecules, CLBZ and CLF, are described in [22]. Descriptions of the parameterization of coarse-grained (CG) forcefields for P3HT, and PffBT4T-2OD may be found in Section 4.2 of [2]. This thesis section also contains details of the parameterization of a CG forcefield for O-IDTBR, from which the CG forcefield for C16-IDTBT was adapted by removing the 3-ethylrhodanine terminal groups. These forcefields may be found at [19]. For convenience, a summary of the construction of the C16-IDTBT forcefield is given here.

The CG builder tool[24] was used to map between atomistic and CG forcefields. For the IDT unit, the central six-membered ring was mapped to two small beads to preserve symmetry. We used the same construction for fused thiophenes as those isolated in P3HT for consistency; A thiophene is mapped in a CoG basis with three tiny beads. We constrained the entire IDT unit in a triangular grid to maintain planarity. The side-chain linking carbons and the first carbon of each side chain were mapped to a small bead. The rest of the sidechains were mapped in the typical manner of alkane chains in the Martini model, with every four consecutive carbon atoms (and attached hydrogens) being modelled by a regular-sized bead. The BT unit was mapped as three tiny beads (benzene part) and one small bead (thiadiazole part). Constraints were used between beads in the rigid, aromatic backbone units, which were connected through harmonic bonds via virtual sites placed at the ring CoG. Except for the IDT-BT dihedral, bonded parameters were tuned by comparing the CG model with the atomistic model. Comparison between degrees-of-freedom associated with bonded parameters during simulations performed using the two forcefields are shown in **Figure S11** and **Figure S12**. The IDT-BT dihedral was independently parameterised against DFT calculations, at the wB97X-D/6-311++G(d,p) level of theory, as shown in **Figure S13**.

Several points of reference were used to validate the CG model. Firstly, bead types were chosen to reproduce the experimental free energies of transfer between water and 1-octanol (See Table 4.1 in [2]). In addition, the packing structure of O-IDTBR in its crystalline phase was compared to experiments. The unit cell parameters were shown to deviate from experimental values by 2.5% and -7.3% for the atomistic and CG models respectively. As introduced in Section 2.1 of the main paper, we also used the CG model to calculate the persistence length of C16-IDTBT chains in solution and compared this to experiments, finding value of 28 ± 7 nm and 6.8 nm < P < 39 nm respectively. This result is discussed further in **Section S5.**

Finally, we validated our forcefields by calculating the densities of thin films models produced by the solvent evaporation procedure. These densities can be found in **Table S2**. Although experimental measurements of thin film densities are rare, we consider the densities for the modelled films to be realistic.

Section S5: Persistence Length Calculation from Molecular Dynamics

The employed method for calculation of persistence length (P) from an MD trajectory of a single polymer chain immersed in solution may be found in Section 3.2.3 of [2]. A summary of the method is given below.

The P of a polymer can be computed as an angle correlation function quantifying the mean angle between segment tangents (see **Figure S14**). If we represent these tangents by unit vectors, $\hat{\boldsymbol{v}}_i$ across the ith segment, the angle Θ_{ij} between segments i and j averaged over an MD trajectory is given as

$$\langle \cos \Theta_{ij} \rangle = \langle \hat{\boldsymbol{v}}_i \cdot \hat{\boldsymbol{v}}_j \rangle. \tag{8}$$

Keeping i constant as the first segment and letting j run across the remaining $N-1$ segments gives an estimate of the relative bending of the polymer, i.e. its P, with respect to its first repeat unit when the orientations of segment i and j lose correlation.

To minimize potential edge effects, an additional averaging step is introduced. Defining the index n = 0, 1, …, $N-1$ describing a pairwise "gap" between the segments, the arithmetic "gap"-means of the trajectory averages are given as

$$C(\Theta, n) = \frac{1}{N-n} \sum_{i=1}^{N-n} \langle \hat{\boldsymbol{v}}_i \cdot \hat{\boldsymbol{v}}_{i+1} \rangle, \tag{9}$$

where we introduce the angle correlation function $C(\Theta, n)$. Under assumption that fluctuations of the chain are Brownian, the angle correlation function will decay exponentially[25]. The persistence length, $P = n_p l$ (in units of distance, where l is the segment length) is then related to the characteristic scale of this decay:

$$C(\Theta, n) \approx e^{-n/n_p}. \tag{10}$$

This approximation allows the angle correlation function to be extrapolated and thus gives access to an approximate P when the chains used in simulation are shorter than their persistence length, as was found for C16-IDTBT.

Figure S14 reveals the large errors that can apply to data points when performing this method. A full description of the error analysis process may be found in Appendix 3.6 of [2].

To collect data from which to compute P, a CG MD simulation of a single C16-IDTBT 24mer was performed. The polymer chain was immersed in a box of chloroform and simulated for 1 μs. From this, we calculated $P = 28.0 \pm 6.7$ nm.

P was also calculated using the atomistic C16-IDTBT model. Data was collected from a 100 ns simulation of a 12mer in chloroform. Two alternative schemes were used to relate the segment vectors to the backbone geometry. With vectors extending across the BT units parallel to the intermonomer bonds, we found $P = 64.7 \pm 21.6$ nm. With vectors going from the CoG of the IDT unit of one monomer to the next, we found $P = 57.8 \pm 19.9$ nm.

There are several of possible reasons for why the values of P calculated from the CG and atomistic models are different. Although the majority of parameter values in the CG forcefield

were based on comparisons with the atomistic forcefield (See **Section S4.2**, **Figure S11**, and **Figure S12**), a key exception is the parameters controlling the IDT-BT dihedral. The behaviour of this dihedral has a significant effect on the persistence length of the polymer. There are also two further reasons related to the simulation setup (rather than the specifics of the forcefield).

Firstly, the timescales of the CG simulation were 10 times longer than those of atomistic simulations (1 μs vs 100 ns). This is important when assessing persistence lengths, because shorter simulations times may exclude conformational fluctuations involving the slowest vibrational modes. This can reduce the accuracy of persistence length calculations because such slow modes can have a large influence on persistence length. Secondly, the length scales of the CG simulation were longer than those of the atomistic simulation. We simulated a 24mer chain in the former, and a 12mer in the latter. Shorter chain lengths can reduce the accuracy of the calculations, particularly when the persistence length is predicted to be longer than the chains simulated, as was the case for our atomistic simulations. Because of these two simulation-related reasons, we consider the P value calculated using the CG model to be the most reliable. Hence, this value was quoted in Section 2.1 of the main paper.

Section S6: Molecular Dynamics Simulation of Chains on Surfaces

6.1. Procedure Details

The number density of gold atoms in the substrate was set to 60 nm^{-3} (corresponding to the standard mass density of gold). Simulations were performed in an NVT ensemble (velocity rescaling thermostat, τ = 0.1 ps). A 1 fs time step was used for the leap-frog integrator. Non-bonded interactions were treated with the potential-shift Verlet scheme, with a cut-off of 2.7 nm. Periodic boundary conditions (PBCs) were in place in the x- and y-directions.

6.2. Crossing Angle Definition

Crossing angles, θ, were found by first identifying centres-of-geometry (CoG) of all 6-membered rings along the C16-IDTBT backbones (or all thiophene rings in the case of P3HT). The two closest ring-centres between the backbones were then located (yellow beads in **Figure S16**). The CoG rings on either side (green beads) of these contact rings were then used to define vectors expressing the backbone directions in the local vicinity of the contact (green arrows). If a contact ring was also the final ring in the backbone, the backbone vector was found using the contact ring CoG and the CoG of the adjacent ring (yellow bead to green bead). The crossing angle is the angle between these backbone vectors.

6.3. S and T Stacked Backbones

To determine whether the chain backbones are in and S or T arrangement (see **Figure S18** (a)) at a contact, we find the angle between the backbone plane vectors of the closest units on the two chains. Units may be BT or IDT. The results given in **Figure S19** are based on 200 simulations of pairs of C16-IDTBT 12mer chains deposited onto substrate.

Figure S19 (a) shows the contributions to interaction energy are dominated by interactions between sidechains and the substrate. **Figure S19** (b) shows that, if only interactions between the backbones of the two polymer chains are considered, we recover the expected relationship with the angle between the planes of the backbones; it is energetically favourable for backbones

to be in and 'S' or π-stacked arrangement. However, if we now consider **Figure S19** (c), which shows the relationship between the interaction energy between sidechains and the substrate, and the angle between the planes of the backbones, we see that there is a large degree of noise in the interaction energies, and that there is no obvious trend. Because the polymer backbones are much less influenced by entropic effects than sidechains, it may be that, at long timescales, the preference for an S-stacked arrangement between the backbones will eventually lead to most contact points adopting this configuration, thus explaining why this is the case in the physical contacts probed by STM. However, on the shorter timescales accessible by MD, contact arrangements will be dominated by the larger interaction energy between sidechains and substrate, which show no preference for S-stacked backbones, and the random entropic motion of the sidechains. Thus, we see a wider variety in the angles between the planes of the backbones at the conclusion of the MD simulations. In addition, the discrepancy between simulated and experimental observations may be related to the inability of MD forcefields to fully capture π-π interactions that would occur at an S-stacked contact.

Section S7: Molecular Dynamics Simulation of Solid-State Microstructure

7.1. Procedure Details

Details of the systems for which film formation was simulated using CG MD are summarised in **Table S2** Most drying steps involved the removal of 1.25% of the remaining solvent molecules. It was required that a minimum of 0.0125% of the original number of solvent molecules be removed at each step. Therefore, the decrease in solvent was exponential for most of the procedure but switched to linear once only a small amount of solvent remained.

During each drying step, the system was equilibrated for 0.5 ns in an NVT ensemble (Berendsen thermostat, τ = 2 ps) and 4.0 ns in an NPT ensemble (Berendsen thermostat, τ = 2 ps, and Berendsen barostat, τ = 4 ps), followed by a 3.0 ns NPT production run (Berendsen thermostat, τ = 2 ps, Parrinello-Rahman barostat, τ = 15 ps). The annealing step consisted of a 100 ns NPT simulation. Annealing temperatures were 373K (C16-IDTBT 24mers), 333K (C16-IDTBT 12mers and P3HT 48mers), 363K (PffBT4T-2OD 12mers). During all NPT stages, semi-isotropic pressure coupling was enabled, applying atmospheric pressure in the z-direction. A 20 fs time step was used for the leap-frog integrator. Non-bonded interactions were treated with the potential-shift Verlet scheme, with a cut-off of 1.1 nm, as recommended with Martini[2].

This procedure was used to produce one C16-IDTBT 24mer structure, and three structures each for C16-IDTBT 12mers, P3HT 48mers and PffBT4T-2OD 12mers. Unless otherwise specified, results are displayed only for one structure from each of these sets of three. The other two structures in each case were used to validate observed microstructural trends in e.g., the distribution of crossing angles and interchain separations. Coupling calculations were performed on only one structure for each material case (except for PffBT4T-2OD, for which none were performed), due to the high number of contacts in each system, and the high computational expense of DFT.

7.2. Backmapping

Backmapping from Martini to OPLS-AA forcefields was performed using the script *backward-v5.py* and wrapper script *initram-v5.sh* (version 0.1) detailed in [26]. Briefly, this

method works by using a user-made mapping file to perform a geometric projection between the CG beads and atoms. This projection acts as a ‘first guess’ for the correct position of the atoms and may be rough. Subsequently, the atom positions are improved by performing 1) an energy minimization run, where non-bonded interactions are excluded (to fix bond lengths and angles), 2) an energy minimization run including non-bonded interactions, and 3) a series of extremely short NVT simulations.

Backmapping was performed on the full thin film structures. The following parameters were modified from their default values: -kick 0.1 -em 100 -nb 5000.

7.3. Identifying Interchain Contacts

Two chains were considered to be in contact if, between the two backbones, there was at least one pair of π-system atoms separated by ≤ 8 Å. Once a chain pair was identified in this way, the pair was retested to locate the single or multiple points of contact between the two chains. An algorithm was created to analyse chain pairs and decide how best to divide them into segments (2mers for IDTBT, 8mers for P3HT), and then to pair segments into interchain contact structures. The algorithm was designed to find and pair up segments between the two chains in a manner that would maximise the degree of contact summed across all contact structure while ensuring that no segment is used in multiple contact structures (to avoid ‘double counting’ of coupling between the chains). The algorithm carried out the following steps:

1. Create a ‘value matrix’ with dimensions equal to the number of monomers in each of the two chains (which we label A and B). This value matrix is populated using a ‘profit function’ to estimate the potential coupling between each monomer on A, with each monomer on B. The profit function drops off exponentially with separation distance and returns zero beyond a cutoff distance of 1 nm.
2. The value matrix is divided into submatrices by slicing along any rows or columns that are filled with zeros. Any submatrices containing only zeros are discarded. Each of the remaining submatrices represents a location where there are monomers in contact between the two chains, which are from here referred to as ‘islands’. Different islands represent different positions along the two chains that are separated by consecutive non-interacting monomers (i.e. contact 3 in the IDTBT images in **Figure S20**, which is separated from contacts 1 and 2).
3. For each island, several different possible segment pair choices are compared, by summing up the total value each choice would return. This is equivalent to sliding the choice of segments back and forth on each chain backbone in increments of one monomer, and in each position calculating the total degree of contact between the segments on each chain.
4. The choice of segment pairs that maximise the degree of contact between chains is selected and outputted. Examples of the results of this selection process may be seen in **Figure S20**.

With this method, 5787 contacts were identified in the C16-IDTBT 24mer model, 2821 in the C16-IDTBT 12mer model, 6062 in the P3HT 48mer model, and 4512 in the PffBT4T-2OD model. These contacts were then analysed further to understand microstructural trends and were also used when performing interchain coupling calculations (see **Section S8**).

In some cases, we limited our analysis to only *close contacts*, which we defined as a separation between the CoG of 5- or 6-membered rings ≤ 6 Å. These may also be considered as 1st nearest neighbour (NN) contacts (see **Figure S21** (a)). 3365 close contacts were present in the

C16-IDTBT 24mer model, 1661 in the C16-IDTBT 12mer model, 2551 in the P3HT 48mer model, and 1967 in the PffBT4T-2OD model.

Section S8: Contact Interactions

8.1. Coupling Calculations

Contact structures were extracted from the thin film models by removing all atoms not belonging to the paired segments associated with each contact location and capping these segments with terminating hydrogen atoms. Sidechains were shortened to methyl groups. As described previously, segments comprised 2mers for C16-IDTBT and 8mers for P3HT, which are of approximately equal contour length. These lengths were chosen as the maximum length for which thousands of DFT calculations could be performed within limits of reasonable computational expense. In addition, we considered it desirable to use a C16-IDTBT segment length greater than one monomer, as there is evidence that holes delocalize across multiple monomers in the C16-IDTBT backbone [13,27].

The contact structures extracted from the thin film models were briefly optimized before the electronic coupling between the segments was computed. Optimization was performed for 3 steps, at the CAM-B3LYP/cc-pVDZ level of theory, implemented in Gaussian 16-c01-avx[28]. This was followed by three single point calculations, performed to find the molecular orbital structure of the dimer and the two isolated segments. These calculations were performed using the same functional and basis set as the optimization. Coupling between the HOMOs of the two segments was then computed using the projective method described in [29], the code of which may be found at [30].

Section S9: Transport Simulations

All kinetic Monte Carl (KMC) simulations were carried out using the program ToFeT, the code of which may be found at [31].

9.1. Assigning Sites and Edges

Structures used in kinetic Monte Carlo (KMC) transport simulations were derived from backmapped (atomistic) thin film models. Polymer chains were represented by a sequence of sites. Site coordinates were calculated from CoG of π-system atoms belonging to backbone segments. As diagrammed in **Figure S34**, segments comprised 2 repeat units in C16-IDTBT systems, and 8 repeat units in P3HT system. These segments lengths were chosen to match the segment lengths used in interchain coupling calculations (and thus represent approximately equal backbone lengths between the two CPs).

Edges are included between site pairs that are electronically coupled. Intrachain edges are included between adjacent sites within the same chain. Interchain edges are included between any pair of segments when a J_{inter} calculation was performed between them. The interchain edge is placed between the sites closest to the point of contact previously selected for the J_{inter} calculation.

9.2. Electronic Structure Parameters

Most parameters were kept equal between C16-IDTBT and P3HT for transport simulations. This was done to isolate microstructural effects as the only variable between the different CP systems. Since J_{inter} is heavily dependent on backbone packing geometry, we considered the sets of calculated J_{inter} values to be aspects of the microstructure, and therefore allowed these to be different between systems (along with e.g., chain conformations and contact locations). All other electronic structure parameters (site energies (ε), J_{intra}, reorganization energy (λ)) and simulation parameters (field strength (F), and temperature (T)) were kept equal between the polymer systems or were varied across the same range of values. Values for the electronic structure parameters were chosen to represent C16-IDTBT, as this was the focus of our study, but were also applied when simulating transport through P3HT.

ε was set to zero across all sites, representing a situation of negligible energetic disorder. This was done on the basis that C16-IDTBT has been found to display '[energetic] disorder-free transport' in experiments conducted by Venkateshvaran *et al.*[13].

J_{intra} was varied across multiple transport simulations. A single value was used for all intrachain edges. Values between 10^{-3} eV and 10^{-1} eV were chosen to represent a realistic range of intrachain mobilities (μ_{intra}), including those of both C16-IDTBT and P3HT (see **Section S9.3** for details on relationship between J_{intra} and μ_{intra})

J_{inter} was calculated for each interchain contact, as detailed in **Section S8**.

λ was set to 0.3 eV for all edges. This value was chosen as a compromise to account for 1) calculations of the energy difference between an IDTBT monomer in its neutral versus positively charged state and 2) temperature dependent mobility experiments reported in the literature (see **Section S9.4** for further details).

9.3. Relationship between μ_{intra} and J_{intra}

Consider a system comprising a linear chain of sites separated by a, all with equal ε, where each site is coupled to its immediate neighbours with strength J_{intra}. Under the influence of an applied electric field, F, a hole placed on one of these sites can hop forward (+) or backwards (-) along the chain, with respect to the field direction. The forward and backwards hopping rates are given by

$$\Gamma_{\pm} = \frac{|J_{\text{intra}}|^2}{\hbar}\sqrt{\frac{\pi}{\lambda kT}}\exp\left(-\frac{(\lambda \pm eaF)^2}{4\lambda k_B T}\right). \tag{11}$$

In the limit of low field strength, this may be approximated by

$$\Gamma_{\pm} = \frac{|J_{\text{intra}}|^2}{\hbar}\sqrt{\frac{\pi}{\lambda kT}}\exp\left(\frac{-\lambda}{4k_B T}\right)\left(1 \pm \frac{eaF}{2k_B T}\right). \tag{12}$$

The net velocity of holes, v, is related to the forward and backwards hopping rates by

$$v = a(\Gamma_{+} - \Gamma_{-}) = \frac{|J_{\text{intra}}|^2}{\hbar}\sqrt{\frac{\pi}{\lambda k_B T}}\exp\left(\frac{-\lambda}{4k_B T}\right)\frac{ea^2 F}{k_B T}, \tag{13}$$

From which we can find the intrachain mobility, by dividing by the field strength:

$$\mu_{\text{intra}} = \frac{|J_{\text{intra}}|^2}{\hbar}\sqrt{\frac{\pi}{\lambda k_B T}}\exp\left(\frac{-\lambda}{4k_B T}\right)\frac{ea^2}{k_B T} \tag{14}$$

For our systems, a is set by the length of segments used when dividing a chain into sites. We use a value of 3 nm, approximately the length of 2 IDTBT repeat units, or 8 P3HT repeat units.

9.4. Calculation of λ

We calculated the single molecule λ of IDTBT (with sidechains reduced to hydrogen atoms) for different conformations and chain lengths (1mer to 6mer) based on the optimized ground state and single positively charged state geometries using the CAM-B3LYP functional at Dunning's cc-pVDZ level as implemented in Gaussian 16-c01-avx[28]. A comparison after adding Grimme's D3BJ dispersion correction shows minor differences. We found that λ plateaus in the range 370 – 380 meV at the lowest energy conformation as we increase the length of the polymer chain. The λ from normal mode decomposition analysis are in good agreement with the four-point method.

For our KMC simulations, a λ of 300 meV was used, slightly lower than the λ calculated via the approach outlined above. This was chosen as a compromise to account for 1) our calculated value, and 2) the activation energy (E_A) values typically extracted from temperature dependent transport experiments. An E_A of 61 meV was reported by Zhang *et al.*[16], while E_As of 70 meV and 80 meV were reported by Ponder *et al.*[15]. These correspond to λ values of 244 meV, 280 meV and 320 meV respectively, when energetic disorder is neglected.

References

(1) Cendra, C.; Balhorn, L.; Zhang, W.; O'Hara, K.; Bruening, K.; Tassone, C. J.; Steinrück, H.-G.; Liang, M.; Toney, M. F.; McCulloch, I.; Chabinyc, M. L.; Salleo, A.; Takacs, C. J. Unraveling the Unconventional Order of a High-Mobility Indacenodithiophene–Benzothiadiazole Copolymer. *ACS Macro Lett.* **2021**, *10* (10), 1306–1314. https://doi.org/10.1021/acsmacrolett.1c00547.

(2) Gertsen, A. S. Multiscale Modelling of Organic Solar Cell Materials, Technical University of Denmark, DTU, Department of Energy Conversion and Storage, Fysikvej, Building 310, 2800 Kgs. Lyngby Denmark, 2020. www.energy.dtu.dk.

(3) Kwiatkowski, J. J.; Frost, J. M.; Nelson, J. The Effect of Morphology on Electron Field-Effect Mobility in Disordered C60 Thin Films. *Nano Lett.* **2009**, *9* (3), 1085–1090. https://doi.org/10.1021/nl803504q.

(4) Glinka, C. J.; Barker, J. G.; Hammouda, B.; Krueger, S.; Moyer, J. J.; Orts, W. J. The 30 m Small-Angle Neutron Scattering Instruments at the National Institute of Standards and Technology. *J. Appl. Crystallogr.* **1998**, *31* (3), 430–445. https://doi.org/10.1107/S0021889897017020.

(5) Kline, S. R. Reduction and Analysis of SANS and USANS Data Using IGOR Pro. *J. Appl. Crystallogr.* **2006**, *39* (6), 895–900. https://doi.org/10.1107/S0021889806035059.

(6) Kienzle, P. A.; Krycka, J.; Patel, N.; Sahin, I. Bumps (Version 0.9.1), 2023.

(7) SasView/Sasmodels, 2024. https://github.com/SasView/sasmodels (accessed 2024-02-29).

(8) Grell, M.; Bradley, D. d. c.; Long, X.; Chamberlain, T.; Inbasekaran, M.; Woo, E. p.; Soliman, M. Chain Geometry, Solution Aggregation and Enhanced Dichroism in the Liquidcrystalline Conjugated Polymer Poly(9,9-Dioctylfluorene). *Acta Polym.* **1998**, *49* (8), 439–444. https://doi.org/10.1002/(SICI)1521-4044(199808)49:8<439::AID-APOL439>3.0.CO;2-A.

(9) Wong, M.; Hollinger, J.; Kozycz, L. M.; McCormick, T. M.; Lu, Y.; Burns, D. C.; Seferos, D. S. An Apparent Size-Exclusion Quantification Limit Reveals a Molecular Weight Limit in the Synthesis of Externally Initiated Polythiophenes. *ACS Macro Lett.* **2012**, *1* (11), 1266–1269. https://doi.org/10.1021/mz300333f.

(10) Debye, P.; Anderson, H. R., Jr.; Brumberger, H. Scattering by an Inhomogeneous Solid. II. The Correlation Function and Its Application. *J. Appl. Phys.* **1957**, *28* (6), 679–683. https://doi.org/10.1063/1.1722830.

(11) Higgins, J. S.; Benoit, H. C. *Polymers and Neutron Scattering*; Oxford Series on Neutron Scattering in Condensed Matter; Oxford University Press: Oxford, New York, 1997.

(12) Monteiro, M. J. Fitting Molecular Weight Distributions Using a Log-Normal Distribution Model. *Eur. Polym. J.* **2015**, *65*, 197–201. https://doi.org/10.1016/j.eurpolymj.2015.01.009.

(13) Venkateshvaran, D.; Nikolka, M.; Sadhanala, A.; Lemaur, V.; Zelazny, M.; Kepa, M.; Hurhangee, M.; Kronemeijer, A. J.; Pecunia, V.; Nasrallah, I.; Romanov, I.; Broch, K.; McCulloch, I.; Emin, D.; Olivier, Y.; Cornil, J.; Beljonne, D.; Sirringhaus, H. Approaching Disorder-Free Transport in High-Mobility Conjugated Polymers. *Nature* **2014**, *515* (7527), 384–388. https://doi.org/10.1038/nature13854.

(14) Kienzle, P. Neutron Activation and Scattering Calculator. https://www.ncnr.nist.gov/resources/activation/ (accessed 2024-02-29).

(15) Ponder Jr, J. F.; Chen, H.; Luci, A. M. T.; Moro, S.; Turano, M.; Hobson, A. L.; Collier, G. S.; Perdigão, L. M. A.; Moser, M.; Zhang, W.; Costantini, G.; Reynolds, J. R.; McCulloch, I. Low-Defect, High Molecular Weight Indacenodithiophene (IDT) Polymers Via a C–H Activation: Evaluation of a Simpler and Greener Approach to Organic

Electronic Materials. *ACS Mater. Lett.* **2021**, *3* (10), 1503–1512. https://doi.org/10.1021/acsmaterialslett.1c00478.
(16) Zhang, X.; Bronstein, H.; Kronemeijer, A. J.; Smith, J.; Kim, Y.; Kline, R. J.; Richter, L. J.; Anthopoulos, T. D.; Sirringhaus, H.; Song, K.; Heeney, M.; Zhang, W.; McCulloch, I.; DeLongchamp, D. M. Molecular Origin of High Field-Effect Mobility in an Indacenodithiophene–Benzothiadiazole Copolymer. *Nat. Commun.* **2013**, *4* (1), 2238. https://doi.org/10.1038/ncomms3238.
(17) Hanwell, M. D.; Curtis, D. E.; Lonie, D. C.; Vandermeersch, T.; Zurek, E.; Hutchison, G. R. Avogadro: An Advanced Semantic Chemical Editor, Visualization, and Analysis Platform. *J. Cheminformatics* **2012**, *4* (1), 17. https://doi.org/10.1186/1758-2946-4-17.
(18) Gertsen, A. S.; Sørensen, M. K.; Andreasen, J. W. Nanostructure of Organic Semiconductor Thin Films: Molecular Dynamics Modeling with Solvent Evaporation. *Phys. Rev. Mater.* **2020**, *4* (7), 075405. https://doi.org/10.1103/PhysRevMaterials.4.075405.
(19) Gertsen, A. S. Molecular Dynamics Force Fields for Organic Solar Cell Materials. **2021**. https://doi.org/10.11583/DTU.c.5254236.v1.
(20) Zheng, Y.; Wang, G.-J. N.; Kang, J.; Nikolka, M.; Wu, H.-C.; Tran, H.; Zhang, S.; Yan, H.; Chen, H.; Yuen, P. Y.; Mun, J.; Dauskardt, R. H.; McCulloch, I.; Tok, J. B.-H.; Gu, X.; Bao, Z. An Intrinsically Stretchable High-Performance Polymer Semiconductor with Low Crystallinity. *Adv. Funct. Mater.* **2019**, *29* (46), 1905340. https://doi.org/10.1002/adfm.201905340.
(21) Alessandri, R.; Grünewald, F.; Marrink, S. J. The Martini Model in Materials Science. *Adv. Mater.* **2021**, *33* (24), 2008635. https://doi.org/10.1002/adma.202008635.
(22) Alessandri, R.; Barnoud, J.; Gertsen, A. S.; Patmanidis, I.; de Vries, A. H.; Souza, P. C. T.; Marrink, S. J. Martini 3 Coarse-Grained Force Field: Small Molecules. *Adv. Theory Simul.* **2022**, *5* (1), 2100391. https://doi.org/10.1002/adts.202100391.
(23) Alessandri, R.; Uusitalo, J. J.; de Vries, A. H.; Havenith, R. W. A.; Marrink, S. J. Bulk Heterojunction Morphologies with Atomistic Resolution from Coarse-Grain Solvent Evaporation Simulations. *J. Am. Chem. Soc.* **2017**, *139* (10), 3697–3705. https://doi.org/10.1021/jacs.6b11717.
(24) Barnoud, J. *CG Builder*. https://jbarnoud.github.io/cgbuilder/ (accessed 2024-05-29).
(25) Doi, M. *Introduction to Polymer Physics*, 5th ed.; Oxford University Press, 2004.
(26) Wassenaar, T. A.; Pluhackova, K.; Böckmann, R. A.; Marrink, S. J.; Tieleman, D. P. Going Backward: A Flexible Geometric Approach to Reverse Transformation from Coarse Grained to Atomistic Models. *J. Chem. Theory Comput.* **2014**, *10* (2), 676–690. https://doi.org/10.1021/ct400617g.
(27) Zhang, W.; Smith, J.; Watkins, S. E.; Gysel, R.; McGehee, M.; Salleo, A.; Kirkpatrick, J.; Ashraf, S.; Anthopoulos, T.; Heeney, M.; McCulloch, I. Indacenodithiophene Semiconducting Polymers for High-Performance, Air-Stable Transistors. *J. Am. Chem. Soc.* **2010**, *132* (33), 11437–11439. https://doi.org/10.1021/ja1049324.
(28) Frisch, M. J.; Trucks, G. W.; Schlegel, H. B.; Scuseria, G. E.; Robb, M. A.; Cheeseman, J. R.; Scalmani, G.; Barone, V.; Petersson, G. A.; Nakatsuji, H.; Li, X.; Caricato, M.; Marenich, A. V.; Bloino, J.; Janesko, B. G.; Gomperts, R.; Mennucci, B.; Hratchian, H. P.; Ortiz, J. V.; Izmaylov, A. F.; Sonnenberg, J. L.; Williams-Young, D.; Ding, F.; Lipparini, F.; Egidi, F.; Goings, J.; Peng, B.; Petrone, A.; Henderson, T.; Ranasinghe, D.; Zakrzewski, V. G.; Gao, J.; Rega, N.; Zheng, G.; Liang, W.; Hada, M.; Ehara, M.; Toyota, K.; Fukuda, R.; Hasegawa, J.; Ishida, M.; Nakajima, T.; Honda, Y.; Kitao, O.; Nakai, H.; Vreven, T.; Throssell, K.; Montgomery, J. A., Jr.; Peralta, J. E.; Ogliaro, F.; Bearpark, M. J.; Heyd, J. J.; Brothers, E. N.; Kudin, K. N.; Staroverov, V. N.; Keith, T. A.; Kobayashi, R.; Normand, J.; Raghavachari, K.; Rendell, A. P.; Burant, J. C.; Iyengar, S. S.; Tomasi,

J.; Cossi, M.; Millam, J. M.; Klene, M.; Adamo, C.; Cammi, R.; Ochterski, J. W.; Martin, R. L.; Morokuma, K.; Farkas, O.; Foresman, J. B.; Fox, D. J. Gaussian 16 Revision C.01, 2016.

(29) Baumeier, B.; Kirkpatrick, J.; Andrienko, D. Density-Functional Based Determination of Intermolecular Charge Transfer Properties for Large-Scale Morphologies. *Phys. Chem. Chem. Phys.* **2010**, *12* (36), 11103. https://doi.org/10.1039/c002337j.

(30) Kirkpatrick, J. Jenny-Nelson-Group/Counterpoise-J, 2021. https://github.com/Jenny-Nelson-Group/counterpoise-J (accessed 2023-12-20).

(31) Kwiatkowski, J. J.; Coker, J. F. Jfcoker/Tofet, 2021. https://github.com/jfcoker/tofet (accessed 2023-12-20).
